# Supplementary material for: Transcriptomic and Proteomic Profiles for Elucidating Cisplatin Resistance in Head-and-Neck Squamous Cell Carcinoma
Source: Cancers (Basel). 2022 Nov 9;14(22):5511. doi: 10.3390/cancers14225511 (PMC9688094; doi:10.3390/cancers14225511)
Supplement: Supplementary file 1 [file cancers-14-05511-s001.zip › cancers-2015248-supplementary.pdf]

Supplementary Figure legends

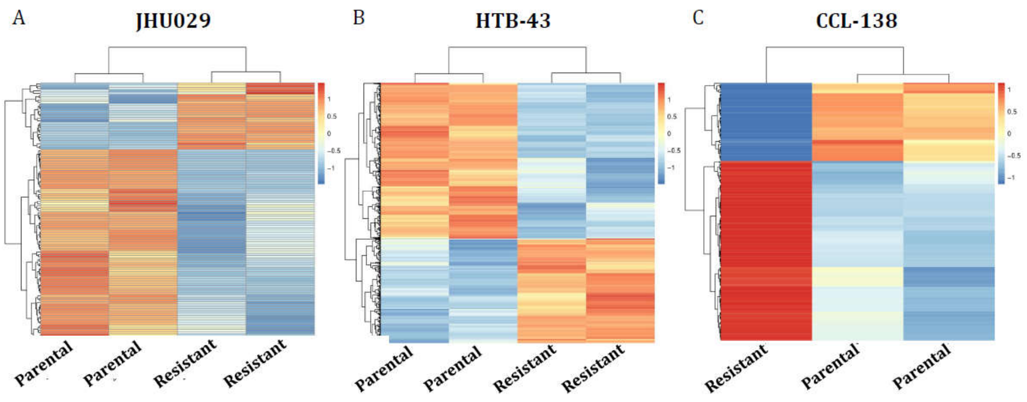

Supplementary Figure S1. Heatmap of the RNA-seq data: (**A**) Heatmap of the JHU029 cells. Note the difference between resistant and parental cells; (**B**) Heatmap of HTB-43 cells. Note the difference between resistant and parental cells; (**C**) Heatmap of the CCL-138 cells. Note the difference between resistant and parental.

A

RNA-seq				
Cells	Row names	Gene name	Symbol	qRT-PCR
JHU029	ENSG00000163347	claudin 1	CLDN1	CLDN1
	ENSG00000099399	MAGE family member B2	MAGEB2	MAGEB2
HTB-43	ENSG00000272398	CD24 molecule	CD24	CD24
	ENSG00000086548	CEA cell adhesion molecule 6	CEACAM6	CEACAM6
CCL-138	ENSG00000125538	interleukin 1 beta	ILB1	ILB1
	ENSG00000187608	ISG15 ubiquitin like modifier	ISG15	ISG15

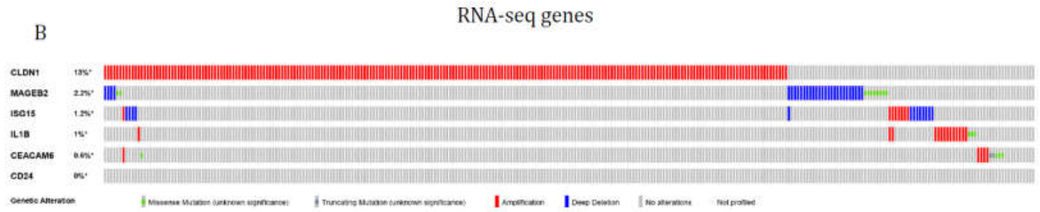

Supplementary Figure S2. *In silico* study of gene status: (**A**) Summary of selected genes for qRT–PCR; (**B**) Genomic alteration of the indicated genes according to the Oncoprint dataset.

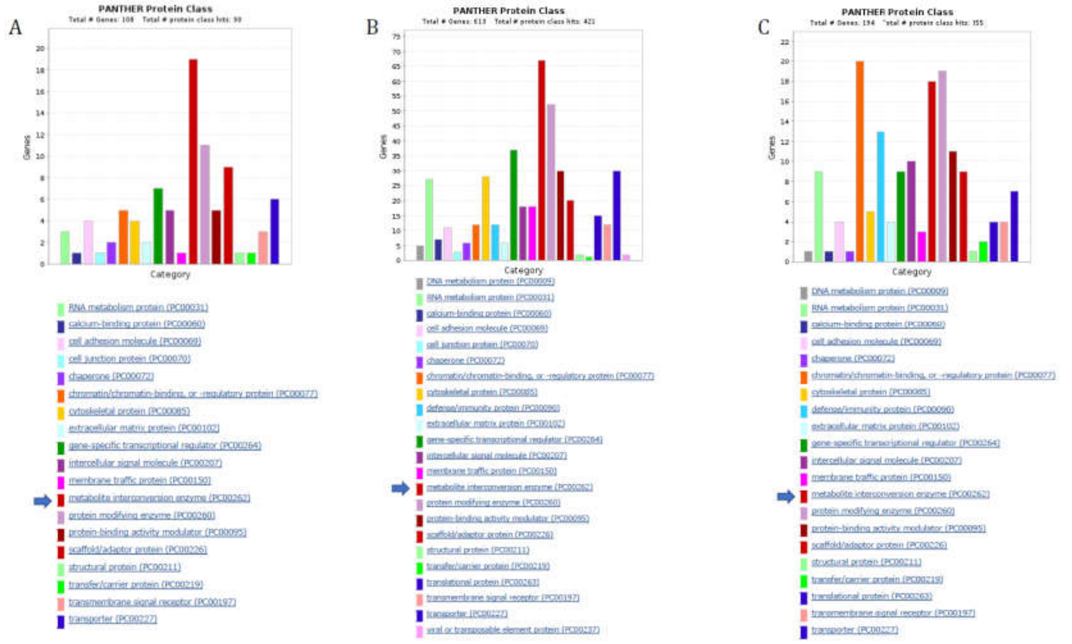

Supplementary Figure S3. Protein classification of proteins encoded by deregulated genes identified by RNA-seq in JHU020 cells (**A**), HTB-43 cells (**B**) and CCL-138 cells (**C**).

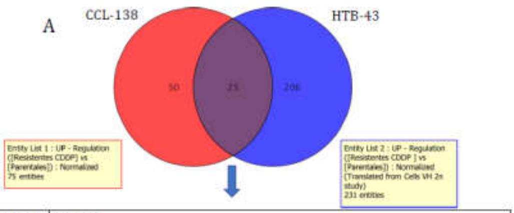

Swiss-Prot ID	Compound
Q9Y6K5	2'-5'-oligoadenylate synthase 3 OS=Homo sapiens GN=OAS3 PE=1 SV=3 - [OAS3_HUMAN]
Q99798	Aconitate hydratase, mitochondrial OS=Homo sapiens GN=ACO2 PE=1 SV=2 - [ACON_HUMAN]
O75608	Acyl-protein thioesterase 1 OS=Homo sapiens GN=LYPLA1 PE=1 SV=1 - [LYPA1_HUMAN]
P51572	B-cell receptor-associated protein 31 OS=Homo sapiens GN=BCAP31 PE=1 SV=3 - [BAP31_HUMAN]
P00918	Carbonic anhydrase 2 OS=Homo sapiens GN=CA2 PE=1 SV=2 - [CAH2_HUMAN]
Q00610	Clathrin heavy chain 1 OS=Homo sapiens GN=CLTC PE=1 SV=5 - [CLH1_HUMAN]
P15311	Ezrin OS=Homo sapiens GN=EZR PE=1 SV=4 - [EZRI_HUMAN]
Q08380	Galectin-3-binding protein OS=Homo sapiens GN=LGALS3BP PE=1 SV=1 - [LG3BP_HUMAN]
Q9Y2T3	Guanine deaminase OS=Homo sapiens GN=GDA PE=1 SV=1 - [GUAD_HUMAN]
Q96AG4	Leucine-rich repeat-containing protein 59 OS=Homo sapiens GN=LRRC59 PE=1 SV=1 - [LRC59_HUMAN]
Q7Z406	Myosin-14 OS=Homo sapiens GN=MYH14 PE=1 SV=2 - [MYH14_HUMAN]
P80188	Neutrophil gelatinase-associated lipocalin OS=Homo sapiens GN=LCN2 PE=1 SV=2 - [NGAL_HUMAN]
Q96TA1	Niban-like protein 1 OS=Homo sapiens GN=FAM129B PE=1 SV=3 - [NIBL1_HUMAN]
Q56VL3	OCIA domain-containing protein 2 OS=Homo sapiens GN=OCIAD2 PE=1 SV=1 - [OCAD2_HUMAN]
P62942	Peptidyl-prolyl cis-trans isomerase FKBP1A OS=Homo sapiens GN=FKBP1A PE=1 SV=2 - [FKB1A_HUMAN]
P55786	Puromycin-sensitive aminopeptidase OS=Homo sapiens GN=NPEPPS PE=1 SV=2 - [PSA_HUMAN]
Q15907	Ras-related protein Rab-11B OS=Homo sapiens GN=RAB11B PE=1 SV=4 - [RB11B_HUMAN]
P61019	Ras-related protein Rab-2A OS=Homo sapiens GN=RAB2A PE=1 SV=1 - [RAB2A_HUMAN]
P10114	Ras-related protein Rap-2a OS=Homo sapiens GN=RAP2A PE=1 SV=1 - [RAP2A_HUMAN]
P52566	Rho GDP-dissociation inhibitor 2 OS=Homo sapiens GN=ARHGDIB PE=1 SV=3 - [GDIR2_HUMAN]
Q9Y6N5	Sulfide:quinone oxidoreductase, mitochondrial OS=Homo sapiens GN=SQRDL PE=1 SV=1 - [SQRD_HUMAN]
Q9UDY2	Tight junction protein ZO-2 OS=Homo sapiens GN=TJP2 PE=1 SV=2 - [ZO2_HUMAN]
P61586	Transforming protein RhoA OS=Homo sapiens GN=RHOA PE=1 SV=1 - [RHOA_HUMAN]
Q9BVK6	Transmembrane emp24 domain-containing protein 9 OS=Homo sapiens GN=TMED9 PE=1 SV=2 - [TMED9_HUN
P23381	Tryptophan--tRNA ligase, cytoplasmic OS=Homo sapiens GN=WARS PE=1 SV=2 - [SYWC_HUMAN]

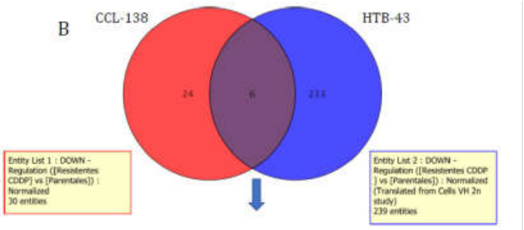

Swiss-Prot ID	Compound
P50895	Basal cell adhesion molecule OS=Homo sapiens GN=BCAM PE=1 SV=2 - [BCAM_HUMAN]
O43175	D-3-phosphoglycerate dehydrogenase OS=Homo sapiens GN=PHGDH PE=1 SV=4 - [SERA_HUMAN]
P33993	DNA replication licensing factor MCM7 OS=Homo sapiens GN=MCM7 PE=1 SV=4 - [MCM7_HUMAN]
Q06830	Peroxiredoxin-1 OS=Homo sapiens GN=PRDX1 PE=1 SV=1 - [PRDX1_HUMAN]
Q13247	Serine/arginine-rich splicing factor 6 OS=Homo sapiens GN=SRSF6 PE=1 SV=2 - [SRSF6_HUMAN]
P50454	Serpin H1 OS=Homo sapiens GN=SERPINH1 PE=1 SV=2 - [SERPH_HUMAN]

Supplementary Figure S4. Proteins commonly deregulated when HTB-43 and CCL-138 resistant cells *versus* parental cells were compared: (**A**) A total of 25 proteins

were commonly found to be upregulated; (**B**) A total of 6 proteins were commonly found to be deregulated.

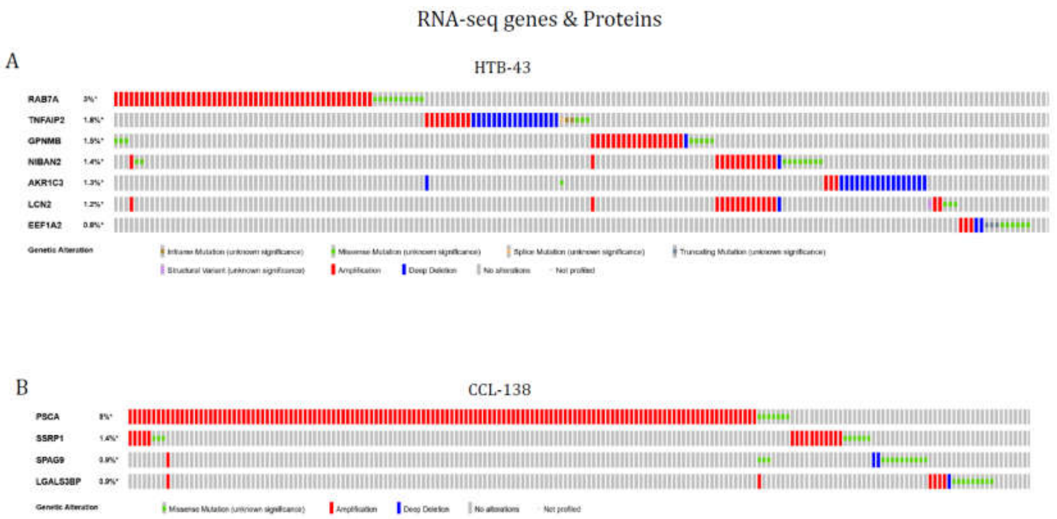

Supplementary Figure S5. *In silico* study of gene status: (**A**) Genomic alteration of the indicated genes according to the Oncoprint dataset found to be deregulated in HTB-43 cells; (**B**) Genomic alteration of the indicated genes according to the Oncoprint dataset found to be deregulated in CCL-138 cells.

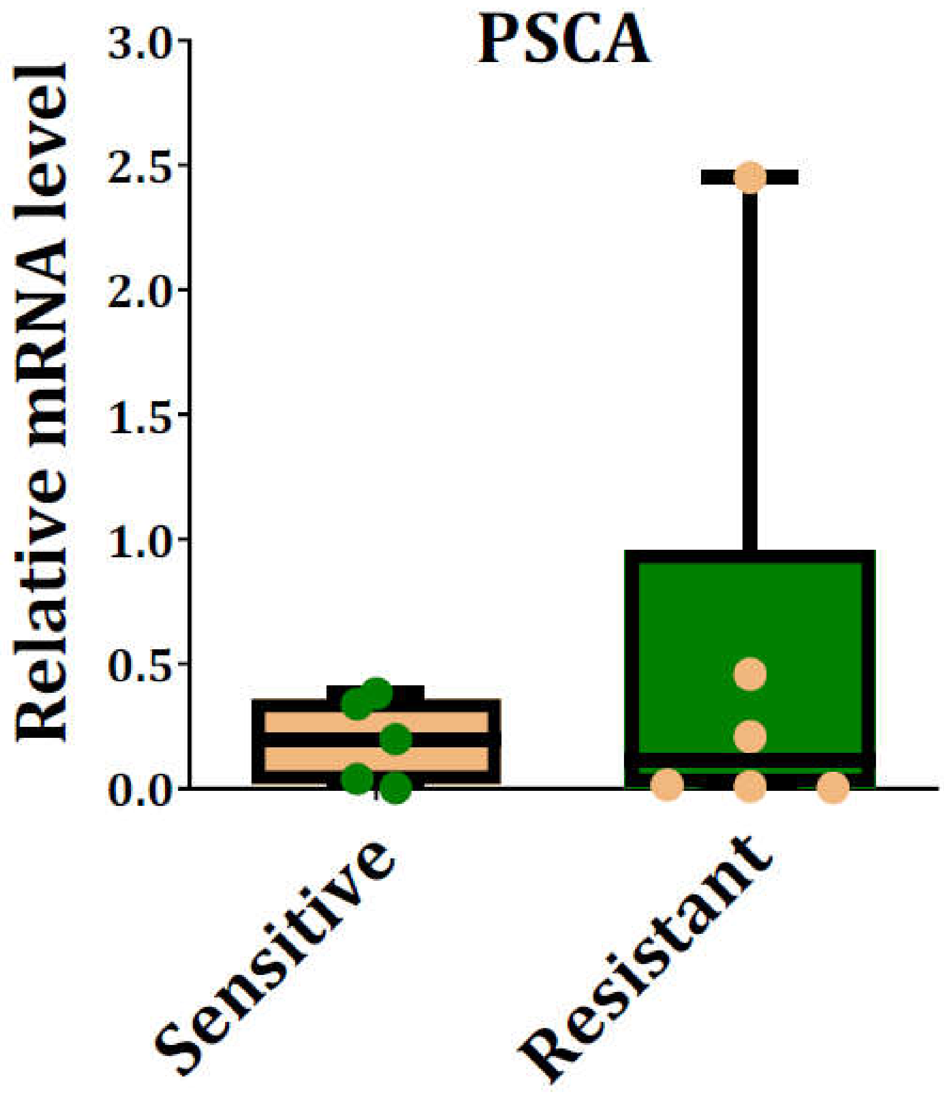

Supplementary Figure S6. *PSCA* gene expression shows a trend to significance when resistant *versus* sensitive patients were compared.

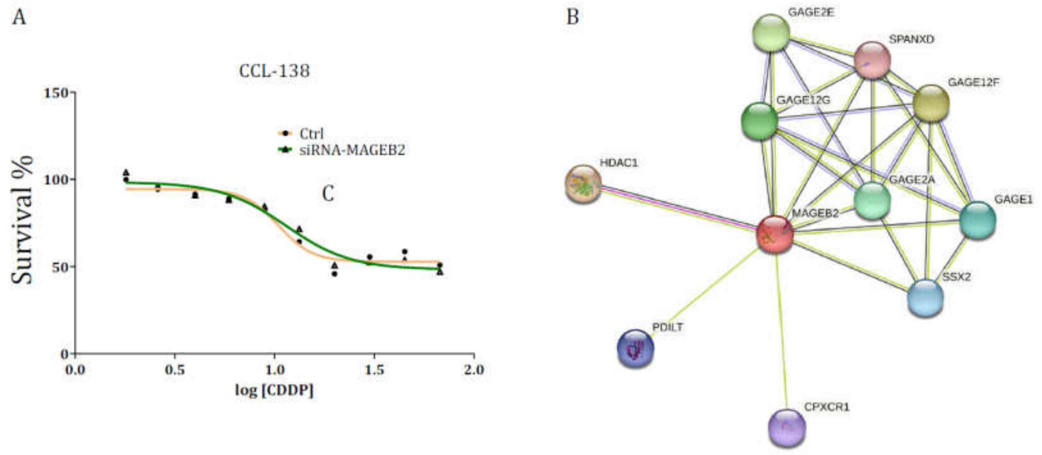

Supplementary Figure S7. Characterization of *MAGEB2*: (A) Lack of sensitization in *MAGEB2* depletion conditions in CCL-138; (**B**) MAGEB2 protein interactions.

Table S1. Patients in whom the RNA study of the 12 selected genes was carried out. Both patients who received chemotherapy and radiotherapy, these treatments were after surgery. QT, chemotherapy; RT, radiotherapy.

Patient	Surgery	Cancer type	Resistant	QT	RT
P2	YES	larynx	YES	NO	YES
P7	YES	larynx	NO	YES	YES
P8	YES	larynx	NO	YES	YES
P15	YES	larynx	YES	YES	YES
P17	YES	larynx	NO	NO	YES
P22	YES	larynx	NO	NO	YES
P29	YES	larynx	NO	NO	YES
P32	YES	larynx	YES	NO	YES
P33	YES	larynx	YES	NO	YES
P42	YES	larynx	YES	YES	YES
P70	YES	larynx	YES	NO	YES

Table S2. Data counts before and after some processing steps. The raw data is available in the NCBI SRA database under the following accession numbers and the BioProject accession code PRJNA893841.

Cell Line	Replicates	Number of raw reads	Number of reads after trimming	Mapping percent after filtering	Accession number in NCBI SRA database
HTB-43 (FaDu) Pharynx primary parental	HTB-43P-1	23755496	9205260	99.97%	SRR22028463
HTB-43 (FaDu) Pharynx primary parental	HTB-43P-2	23662886	9036143	99.96%	SRR22028462
HTB-43 (FaDu) Pharynx primary resistant	HTB-43R-1	30351858	10865117	99.92%	SRR22028461
HTB-43 (FaDu) Pharynx primary resistant	HTB-43R-2	24438226	9050392	99.96%	SRR22028460
CCL-138 (Detroit 562) Pharynx metastatic parental	CCL-138P-1	27025306	10198441	99.96%	SRR22028467
CCL-138 (Detroit 562) Pharynx metastatic parental	CCL-138P-2	26819396	10136993	99.96%	SRR22028466
CCL-138 (Detroit 562) Pharynx metastatic resistant	CCL-138R-1	25446870	9258014	99.95%	SRR22028464
JHU029 Larynx primary parental	JHU029P-1	32029104	9921133	99.96%	SRR22028457
JHU029 Larynx primary parental	JHU029P-2	25886458	12091413	99.95%	SRR22028458
JHU029 Larynx primary resistant	JHU029R-1	21267200	8397834	99.87%	SRR22028459
JHU029 Larynx primary resistant	JHU029R-2	25049098	10108380	99.96%	SRR22028465

Table S3. Deregulated genes resulting from RNA-seq in the JHU029-R cell line compared to the parental line (JHU029). A total of 85 downregulated genes and 30 upregulated genes were found. Green indicates downregulated genes and orange indicates upregulated genes.

Sample No.	Gene Code	Gene name	Chromosome
22	ENSG00000135074	ADAM metallopeptidase domain 19	5
27	ENSG00000122644	ADP ribosylation factor like GTPase 4A	7
64	ENSG00000184254	aldehyde dehydrogenase 1 family member A3	15
13	ENSG00000187134	aldo-keto reductase family 1 member C1	10
14	ENSG00000151632	aldo-keto reductase family 1 member C2	10
15	ENSG00000196139	aldo-keto reductase family 1 member C3	10
65	ENSG00000109107	aldolase, fructose-bisphosphate C	17
9	ENSG00000071073	alpha-1,3-mannosyl-glycoprotein 4-beta-N-acetylglucosaminyltransferase A	2
46	ENSG00000152766	ankyrin repeat domain 22	10
40	ENSG00000165272	aquaporin 3 (Gill blood group)	9
29	ENSG00000106546	aryl hydrocarbon receptor	7
21	ENSG00000183876	arylsulfatase family member I	5
41	ENSG00000165029	ATP binding cassette subfamily A member 1	9
52	ENSG00000186318	beta-secretase 1	11
60	ENSG00000074410	carbonic anhydrase 12	15
12	ENSG00000128510	carboxypeptidase A4	7
62	ENSG00000103888	cell migration inducing hyaluronidase 1	15
5	ENSG00000143320	cellular retinoic acid binding protein 2	1
61	ENSG00000173546	chondroitin sulfate proteoglycan 4	15

26	ENSG00000166446	chromodomain Y like 2	16
21	ENSG00000235162	chromosome 12 open reading frame 75	12
71	ENSG00000176659	chromosome 20 putative open reading frame 197	20
5	ENSG00000163347	claudin 1	3
10	ENSG00000204262	collagen type V alpha 2 chain	2
18	ENSG00000182326	complement C1s	12
16	ENSG00000107562	C-X-C motif chemokine ligand 12	10
8	ENSG00000145824	C-X-C motif chemokine ligand 14	5
56	ENSG00000118971	cyclin D2	12
32	ENSG00000173852	dpy-19 like C-mannosyltransferase 1	7
9	ENSG00000151914	dystonin	6
14	ENSG00000182585	epithelial mitogen	4
69	ENSG00000161671	ER membrane protein complex subunit 10	19
74	ENSG00000179083	family with sequence similarity 133 member A	X
48	ENSG00000149485	fatty acid desaturase 1	11
2	ENSG00000115414	fibronectin 1	2
33	ENSG00000132436	fidgetin like 1	7
3	ENSG00000168386	filamin A interacting protein 1 like	3
31	ENSG00000106080	FKBP prolyl isomerase 14	7
45	ENSG00000151025	G protein-coupled receptor 158	10
7	ENSG00000143891	galactose mutarotase	2
	ENSEMBL	GENENAME	CHR
	ENSEMBL	GENENAME	CHR
24	ENSG00000176153	glutathione peroxidase 2	14
30	ENSG00000136235	glycoprotein nmb	7
72	ENSG00000189060	H1.0 linker histone	22
23	ENSG00000276410	H2B clustered histone 3	6
25	ENSG00000274290	H2B clustered histone 6	6
24	ENSG00000287080	H3 clustered histone 3	6
59	ENSG00000126803	heat shock protein family A (Hsp70) member 2	14
6	ENSG00000143341	hemicentin 1	1
13	ENSG00000170801	HtrA serine peptidase 3	4
39	ENSG00000170961	hyaluronan synthase 2	8
12	ENSG00000115457	insulin like growth factor binding protein 2	2
58	ENSG00000167779	insulin like growth factor binding protein 6	12
66	ENSG00000171401	keratin 13	17
25	ENSG00000178573	MAF bZIP transcription factor	16
83	ENSG00000198681	MAGE family member A1	X
81	ENSG00000213401	MAGE family member A12	X
80	ENSG00000221867	MAGE family member A3	X
79	ENSG00000147381	MAGE family member A4	X
29	ENSG00000099399	MAGE family member B2	X
73	ENSG00000179222	MAGE family member D1	X
38	ENSG00000147676	mal, T cell differentiation protein 2	8
50	ENSG00000196611	matrix metallopeptidase 1	11
54	ENSG00000076706	melanoma cell adhesion molecule	11
78	ENSG00000076770	muscleblind like splicing regulator 3	X
53	ENSG00000149573	myelin protein zero like 2	11
35	ENSG00000135272	MyoD family inhibitor domain containing	7
11	ENSG00000228509	NA	NA
67	ENSG00000279207	NA	NA
4	ENSG00000177551	nescient helix-loop-helix 2	1
84	ENSG00000165246	neuroligin 4 Y-linked	Y
20	ENSG00000112992	nicotinamide nucleotide transhydrogenase	5
7	ENSG00000151014	nocturnin	4
22	ENSG00000074590	NUAK family kinase 1	12
19	ENSG00000173391	oxidized low density lipoprotein receptor 1	12
70	ENSG00000101417	peroxisomal membrane protein 4	20
2	ENSG00000162407	phospholipid phosphatase 3	1
4	ENSG00000188313	phospholipid scramblase 1	3
37	ENSG00000076641	phosphoprotein membrane anchor with glycosphingolipid microdomains 1	8
57	ENSG00000139629	polypeptide N-acetylgalactosaminyltransferase 6	12
44	ENSG00000148344	prostaglandin E synthase	9

19	ENSG00000132356	protein kinase AMP-activated catalytic subunit alpha 1	5
3	ENSG00000142875	protein kinase cAMP-activated catalytic subunit beta	1
51	ENSG00000178202	protein O-glucosyltransferase 3	11
49	ENSG00000123892	RAB38, member RAS oncogene family	11
17	ENSG00000039560	retinoic acid induced 14	5
77	ENSG00000133135	ring finger protein 128	X
23	ENSG00000278771	RNA component of signal recognition particle 7SL3	14
1	ENSG00000143546	S100 calcium binding protein A8	1
16	ENSG00000112902	semaphorin 5A	5
17	ENSG00000150687	serine protease 23	11
18	ENSG00000079215	solute carrier family 1 member 3	5
47	ENSG00000134802	solute carrier family 43 member 3	11
6	ENSG00000151012	solute carrier family 7 member 11	4
55	ENSG00000137642	sortilin related receptor 1	11
34	ENSG00000157214	STEAP2 metalloreductase	7
15	ENSG00000145545	steroid 5 alpha-reductase 1	5
63	ENSG00000182253	synemin	15
28	ENSG00000106537	tetraspanin 13	7
75	ENSG00000000003	tetraspanin 6	X
8	ENSG00000144115	threonine synthase like 2	2
1	ENSG00000186891	TNF receptor superfamily member 18	1
42	ENSG00000181634	TNF superfamily member 15	9
43	ENSG00000136869	toll like receptor 4	9
76	ENSG00000180964	transcription elongation factor A like 8	X
26	ENSG00000106460	transmembrane protein 106B	7
20	ENSG00000167552	tubulin alpha 1a	12
28	ENSG00000128294	tyrosylprotein sulfotransferase 2	22
68	ENSG00000179913	UDP-GlcNAc:betaGal beta-1,3-N-acetylglucosaminyltransferase 3	19
82	ENSG00000147394	zinc finger protein 185 with LIM domain	X
27	ENSG00000197013	zinc finger protein 429	19
36	ENSG00000164684	zinc finger protein 704	8
10	ENSG00000214652	zinc finger protein 727	7
11	ENSG00000234444	zinc finger protein 736	7

Table S4. Deregulated genes resulting from RNA-seq in the HTB-43-R cell line compared to the parental line (HTB-43). A total of 392 downregulated genes and 263 upregulated genes were found. Green indicates downregulated genes and orange indicates upregulated genes.

Sample No.	Gene Code	Gene name	Chromosome
91	ENSG00000116133	24-dehydrocholesterol reductase	1
188	ENSG00000089127	2'-5'-oligoadenylate synthetase 1	12
189	ENSG00000111335	2'-5'-oligoadenylate synthetase 2	12
297	ENSG00000135114	2'-5'-oligoadenylate synthetase like	12
194	ENSG00000112972	3-hydroxy-3-methylglutaryl-CoA synthase 1	5
66	ENSG00000186603	4-hydroxyphenylpyruvate dioxygenase like	1
29	ENSG00000123836	6-phosphofructo-2-kinase/fructose-2,6-biphosphatase 2	1
21	ENSG00000143322	ABL proto-oncogene 2, non-receptor tyrosine kinase	1
210	ENSG00000075624	actin beta	7
261	ENSG00000169129	actin filament associated protein 1 like 2	10
22	ENSG00000162704	actin related protein 2/3 complex subunit 5	1
348	ENSG00000072778	acyl-CoA dehydrogenase very long chain	17
86	ENSG00000151726	acyl-CoA synthetase long chain family member 1	4
58	ENSG00000163638	ADAM metallopeptidase with thrombospondin type 1 motif 9	3
139	ENSG00000168710	adenosylhomocysteinase like 1	1

99	ENSG00000162433	adenylate kinase 4	1
114	ENSG00000153292	adhesion G protein-coupled receptor F1	6
206	ENSG00000112414	adhesion G protein-coupled receptor G6	6
25	ENSG00000143862	ADP ribosylation factor like GTPase 8A	1
55	ENSG00000134108	ADP ribosylation factor like GTPase 8B	3
196	ENSG00000169252	adrenoceptor beta 2	5
2	ENSG00000188157	agrin	1
226	ENSG00000127914	A-kinase anchoring protein 9	7
48	ENSG00000174574	akirin 1	1
69	ENSG00000117448	aldo-keto reductase family 1 member A1	1
166	ENSG00000151632	aldo-keto reductase family 1 member C2	10
167	ENSG00000196139	aldo-keto reductase family 1 member C3	10
96	ENSG00000088035	ALG6 alpha-1,3-glucosyltransferase	1
79	ENSG00000109321	amphiregulin	4
125	ENSG00000162688	amylo-alpha-1, 6-glucosidase, 4-alpha-glucanotransferase	1
126	ENSG00000146416	androgen induced 1	6
45	ENSG00000174501	ankyrin repeat domain 36C	2
245	ENSG00000165272	aquaporin 3 (Gill blood group)	9
217	ENSG00000172379	aryl hydrocarbon receptor nuclear translocator 2	15
197	ENSG00000183876	arylsulfatase family member I	5
184	ENSG00000180801	arylsulfatase family member J	4
159	ENSG00000165029	ATP binding cassette subfamily A member 1	9
51	ENSG00000144452	ATP binding cassette subfamily A member 12	2
164	ENSG00000107331	ATP binding cassette subfamily A member 2	9
36	ENSG00000130770	ATP synthase inhibitory factor subunit 1	1
78	ENSG00000123472	ATP synthase mitochondrial F1 complex assembly factor 1	1
141	ENSG00000116459	ATP synthase peripheral stalk-membrane subunit b	1
4	ENSG00000160072	ATPase family AAA domain containing 3B	1
60	ENSG00000114573	ATPase H+ transporting V1 subunit A	3
141	ENSG00000147416	ATPase H+ transporting V1 subunit B2	8
149	ENSG00000155097	ATPase H+ transporting V1 subunit C1	8
3	ENSG00000175756	aurora kinase A interacting protein 1	1
332	ENSG00000066739	autophagy related 2B	14
382	ENSG00000167601	AXL receptor tyrosine kinase	19
179	ENSG00000043039	BARX homeobox 2	11
175	ENSG00000082153	basic leucine zipper and W2 domains 1	2
374	ENSG00000172270	basigin (Ok blood group)	19
337	ENSG00000169594	basonuclin 1	15
114	ENSG00000142867	BCL10 immune signaling adaptor	1
212	ENSG00000166710	beta-2-microglobulin	15
80	ENSG00000174808	betacellulin	4
248	ENSG00000182240	beta-secretase 2	21
379	ENSG00000130303	bone marrow stromal cell antigen 2	19
81	ENSG00000152785	bone morphogenetic protein 3	4
188	ENSG00000176788	brain abundant membrane attached signal protein 1	5
245	ENSG00000064787	brain enriched myelin associated protein 1	20
261	ENSG00000184515	brain expressed X-linked 5	X
131	ENSG00000164713	brain protein I3	7
184	ENSG00000060982	branched chain amino acid transaminase 1	12
336	ENSG00000064726	BTB domain containing 1	15
328	ENSG00000011114	BTB domain containing 7	14
136	ENSG00000143126	cadherin EGF LAG seven-pass G-type receptor 2	1
220	ENSG00000103381	calcineurin like phosphoesterase domain containing 1	16
320	ENSG00000198668	calmodulin 1	14
123	ENSG00000117519	calponin 3	1
223	ENSG00000180917	cap methyltransferase 2	16
142	ENSG00000116489	capping actin protein of muscle Z-line subunit alpha 1	1
216	ENSG00000103811	cathepsin H	15
5	ENSG00000163131	cathepsin S	1
367	ENSG00000177469	caveolae associated protein 1	17
120	ENSG00000272398	CD24 molecule	6
237	ENSG00000120217	CD274 molecule	9
30	ENSG00000196352	CD55 molecule (Cromer blood group)	1

241	ENSG00000086548	CEA cell adhesion molecule 6	19
58	ENSG00000117399	cell division cycle 20	1
18	ENSG00000070831	cell division cycle 42	1
116	ENSG00000142871	cellular communication network factor 1	1
205	ENSG00000118523	cellular communication network factor 2	6
151	ENSG00000136999	cellular communication network factor 3	8
244	ENSG00000044459	centlein	9
62	ENSG00000047457	ceruloplasmin	3
284	ENSG00000166226	chaperonin containing TCP1 subunit 2	12
222	ENSG00000146731	chaperonin containing TCP1 subunit 6A	7
133	ENSG00000121940	chloride channel CLIC like 1	1
29	ENSG00000169504	chloride intracellular channel 4	1
258	ENSG00000171365	chloride voltage-gated channel 5	X
103	ENSG00000153046	chromodomain Y like	6
224	ENSG00000166446	chromodomain Y like 2	16
113	ENSG00000162642	chromosome 1 open reading frame 52	1
204	ENSG00000227051	chromosome 14 open reading frame 132	14
76	ENSG00000154274	chromosome 4 open reading frame 19	4
189	ENSG00000082213	chromosome 5 open reading frame 22	5
213	ENSG00000128849	cingulin like 1	15
323	ENSG00000165934	cleavage and polyadenylation specific factor 2	14
314	ENSG00000198894	CLOCK interacting pacemaker	14
149	ENSG00000009307	cold shock domain containing E1	1
373	ENSG00000183287	collagen and calcium binding EGF domains 1	18
203	ENSG00000111799	collagen type XII alpha 1 chain	6
345	ENSG00000108561	complement C1q binding protein	17
234	ENSG00000125730	complement C3	19
358	ENSG00000141030	COP9 signalosome subunit 3	17
279	ENSG00000111481	COPI coat complex subunit zeta 1	12
386	ENSG00000214078	copine 1	20
158	ENSG00000106789	coronin 2A	9
77	ENSG00000163739	C-X-C motif chemokine ligand 1	4
50	ENSG00000131236	cyclase associated actin cytoskeleton regulatory protein 1	1
227	ENSG00000105810	cyclin dependent kinase 6	7
156	ENSG00000147883	cyclin dependent kinase inhibitor 2B	9
256	ENSG00000008086	cyclin dependent kinase like 5	X
86	ENSG00000162377	cytochrome c oxidase assembly factor 7 (putative)	1
293	ENSG00000136026	cytoskeleton associated protein 4	12
329	ENSG00000089737	DEAD-box helicase 24	14
219	ENSG00000136271	DEAD-box helicase 56	7
135	ENSG00000257093	DENN domain containing 11	7
102	ENSG00000024526	DEP domain containing 1	1
252	ENSG00000165732	DExD-box helicase 21	10
330	ENSG00000100697	dicer 1, ribonuclease III	14
310	ENSG00000119689	dihydrolipoamide S-succinyltransferase	14
115	ENSG00000153904	dimethylarginine dimethylaminohydrolase 1	1
61	ENSG00000132768	diphthamide biosynthesis 2	1
129	ENSG00000117543	diphthamide biosynthesis 5	1
59	ENSG00000057019	discoidin, CUB and LCCL domain containing 2	3
241	ENSG00000137038	distal membrane arm assembly complex 1	9
80	ENSG00000142700	DMRT like family A2	1
146	ENSG00000118655	DNA cross-link repair 1B	1
187	ENSG00000136048	DNA damage regulated autophagy modulator 1	12
10	ENSG00000160049	DNA fragmentation factor subunit alpha	1
120	ENSG00000117505	down-regulator of transcription 1	1
198	ENSG00000113758	drebrin 1	5
36	ENSG00000143507	dual specificity phosphatase 10	1
227	ENSG00000108861	dual specificity phosphatase 3	17
142	ENSG00000120875	dual specificity phosphatase 4	8
307	ENSG00000119661	dynein axonemal light chain 1	14
207	ENSG00000197102	dynein cytoplasmic 1 heavy chain 1	14
202	ENSG00000151914	dystonin	6
57	ENSG00000117395	EBNA1 binding protein 2	1

79	ENSG00000159658	EF-hand calcium binding domain 14	1
13	ENSG00000142634	EF-hand domain family member D2	1
165	ENSG00000115380	EGF containing fibulin extracellular matrix protein 1	2
24	ENSG00000011007	elongin A	1
59	ENSG00000066322	ELOVL fatty acid elongase 1	1
9	ENSG00000074800	enolase 1	1
19	ENSG00000133216	EPH receptor B2	1
221	ENSG00000146648	epidermal growth factor receptor	7
82	ENSG00000085832	epidermal growth factor receptor pathway substrate 15	1
78	ENSG00000124882	epiregulin	4
56	ENSG00000134109	ER degradation enhancing alpha-mannosidase like protein 1	3
34	ENSG00000196482	estrogen related receptor gamma	1
69	ENSG00000244405	ETS variant transcription factor 5	3
246	ENSG00000101210	eukaryotic translation elongation factor 1 alpha 2	20
46	ENSG00000084623	eukaryotic translation initiation factor 3 subunit I	1
277	ENSG00000063046	eukaryotic translation initiation factor 4B	12
349	ENSG00000132507	eukaryotic translation initiation factor 5A	17
385	ENSG00000242372	eukaryotic translation initiation factor 6	20
127	ENSG00000162694	exostosin like glycosyltransferase 2	1
20	ENSG00000116199	FAM20B glycosaminoglycan xylosylkinase	1
208	ENSG00000177706	FAM20C golgi associated secretory pathway kinase	7
133	ENSG00000196937	FAM3 metabolism regulating signaling molecule C	7
214	ENSG00000157470	family with sequence similarity 81 member A	15
152	ENSG00000147689	family with sequence similarity 83 member A	8
234	ENSG00000168522	farnesyltransferase, CAAX box, alpha	8
211	ENSG00000075618	fascin actin-bundling protein 1	7
265	ENSG00000149485	fatty acid desaturase 1	11
309	ENSG00000119616	FCF1 rRNA-processing protein	14
303	ENSG00000139926	FERM domain containing 6	14
124	ENSG00000156869	ferric chelate reductase 1	1
267	ENSG00000172500	FGF1 intracellular binding protein	11
269	ENSG00000075388	fibroblast growth factor 4	11
182	ENSG00000137440	fibroblast growth factor binding protein 1	4
264	ENSG00000066468	fibroblast growth factor receptor 2	10
72	ENSG00000068078	fibroblast growth factor receptor 3	4
52	ENSG00000115414	fibronectin 1	2
366	ENSG00000141756	FKBP prolyl isomerase 10	17
216	ENSG00000106080	FKBP prolyl isomerase 14	7
94	ENSG00000217128	folliculin interacting protein 1	5
85	ENSG00000052795	folliculin interacting protein 2	4
52	ENSG00000198815	forkhead box J3	1
100	ENSG00000164379	forkhead box Q1	6
211	ENSG00000248905	formin 1	15
121	ENSG00000137942	formin binding protein 1 like	1
313	ENSG00000170345	Fos proto-oncogene, AP-1 transcription factor subunit	14
253	ENSG00000196968	fucosyltransferase 11	10
89	ENSG00000082074	FYN binding protein 1	5
122	ENSG00000111816	fyn related Src family tyrosine kinase	6
132	ENSG00000121957	G protein signaling modulator 2	1
138	ENSG00000065135	G protein subunit alpha i3	1
41	ENSG00000116977	galectin 8	1
154	ENSG00000196505	ganglioside induced differentiation associated protein 2	1
204	ENSG00000152661	gap junction protein alpha 1	6
1	ENSG00000189433	gap junction protein beta 4	1
368	ENSG00000182963	gap junction protein gamma 1	17
71	ENSG00000159592	GC-rich promoter binding protein 1 like 1	1
	ENSEMBL	GENENAME	CHR
	ENSEMBL	GENENAME	CHR
231	ENSG00000147536	GINS complex subunit 4	8
96	ENSG00000113552	glucosamine-6-phosphate deaminase 1	5
104	ENSG00000111846	glucosaminyl (N-acetyl) transferase 2 (I blood group)	6
215	ENSG00000140297	glucosaminyl (N-acetyl) transferase 3, mucin type	15
11	ENSG00000177628	glucosylceramidase beta	1

255	ENSG00000148672	glutamate dehydrogenase 1	10
173	ENSG00000115419	glutaminase	2
296	ENSG00000257218	glutamyl-tRNA amidotransferase subunit C	12
331	ENSG00000182512	glutaredoxin 5	14
143	ENSG00000104687	glutathione-disulfide reductase	8
82	ENSG00000138678	glycerol-3-phosphate acyltransferase 3	4
302	ENSG00000100504	glycogen phosphorylase L	14
129	ENSG00000136235	glycoprotein nmb	7
13	ENSG00000198715	glycosylated lysosomal membrane protein	1
179	ENSG00000063660	glypican 1	2
325	ENSG00000066455	golgin A5	14
326	ENSG00000170270	GON7 subunit of KEOPS complex	14
101	ENSG00000116717	growth arrest and DNA damage inducible alpha	1
238	ENSG00000130513	growth differentiation factor 15	19
224	ENSG00000105793	GTP binding protein 10	7
157	ENSG00000119125	guanine deaminase	9
3	ENSG00000183347	guanylate binding protein family member 6	1
48	ENSG00000144366	GULP PTB domain containing engulfment adaptor 1	2
201	ENSG00000273703	H2B clustered histone 14	6
199	ENSG00000276410	H2B clustered histone 3	6
200	ENSG00000276180	H4 clustered histone 9	6
190	ENSG00000135116	harakiri, BCL2 interacting protein	12
42	ENSG00000118960	HCLS1 binding protein 3	2
192	ENSG00000152137	heat shock protein family B (small) member 8	12
174	ENSG00000144381	heat shock protein family D (Hsp60) member 1	2
64	ENSG00000126107	HECT domain E3 ubiquitin protein ligase 3	1
95	ENSG00000113070	heparin binding EGF like growth factor	5
17	ENSG00000127483	heterochromatin protein 1 binding protein 3	1
22	ENSG00000125944	heterogeneous nuclear ribonucleoprotein R	1
92	ENSG00000049860	hexosaminidase subunit beta	5
31	ENSG00000198830	high mobility group nucleosomal binding domain 2	1
106	ENSG00000095951	HIVEP zinc finger 1	6
125	ENSG00000010818	HIVEP zinc finger 2	6
111	ENSG00000206337	HLA complex P5	6
97	ENSG00000113716	HMG-box containing 3	5
201	ENSG00000215271	homeobox and leucine zipper encoding	14
233	ENSG00000168172	hook microtubule tethering protein 3	8
203	ENSG00000100644	hypoxia inducible factor 1 subunit alpha	14
50	ENSG00000030419	IKAROS family zinc finger 2	2
291	ENSG00000166130	IKBKB interacting protein	12
151	ENSG00000143061	immunoglobulin superfamily member 3	1
23	ENSG00000117318	inhibitor of DNA binding 3, HLH protein	1
181	ENSG00000178035	inosine monophosphate dehydrogenase 2	3
84	ENSG00000109452	inositol polyphosphate-4-phosphatase type II B	4
369	ENSG00000159217	insulin like growth factor 2 mRNA binding protein 1	17
215	ENSG00000136231	insulin like growth factor 2 mRNA binding protein 3	7
278	ENSG00000167779	insulin like growth factor binding protein 6	12
235	ENSG00000171105	insulin receptor	19
90	ENSG00000164171	integrin subunit alpha 2	5
172	ENSG00000091409	integrin subunit alpha 6	2
212	ENSG00000105855	integrin subunit beta 8	7
236	ENSG00000090339	intercellular adhesion molecule 1	19
111	ENSG00000137965	interferon induced protein 44	1
169	ENSG00000125538	interleukin 1 beta	2
164	ENSG00000162892	interleukin 24	1
10	ENSG00000160712	interleukin 6 receptor	1
73	ENSG00000197429	intracisternal A particle-promoted polypeptide	1
185	ENSG00000170561	iroquois homeobox 2	5
1	ENSG00000187608	ISG15 ubiquitin like modifier	1
98	ENSG00000162434	Janus kinase 1	1
236	ENSG00000096968	Janus kinase 2	9
94	ENSG00000177606	Jun proto-oncogene, AP-1 transcription factor subunit	1
383	ENSG00000167754	kallikrein related peptidase 5	19

384	ENSG00000167755	kallikrein related peptidase 6	19
44	ENSG00000025800	karyopherin subunit alpha 6	1
167	ENSG00000114982	KAT8 regulatory NSL complex subunit 3	2
68	ENSG00000114796	kelch like family member 24	3
138	ENSG00000176595	kelch repeat and BTB domain containing 11	8
365	ENSG00000186847	keratin 14	17
364	ENSG00000171345	keratin 19	17
185	ENSG00000170477	keratin 4	12
276	ENSG00000205420	keratin 6A	12
19	ENSG00000235750	KIAA0040	1
218	ENSG00000164542	KIAA0895	7
11	ENSG00000116685	KIAA2013	1
305	ENSG00000126777	kinectin 1	14
62	ENSG00000142945	kinesin family member 2C	1
287	ENSG00000049130	KIT ligand	12
24	ENSG00000159166	ladinin 1	1
250	ENSG00000133424	LARGE xylosyl- and glucuronyltransferase 1	22
43	ENSG00000213626	LBH regulator of WNT signaling pathway	2
324	ENSG00000100600	legumain	14
103	ENSG00000066557	leucine rich repeat containing 40	1
74	ENSG00000132128	leucine rich repeat containing 41	1
88	ENSG00000116212	leucine rich repeat containing 42	1
21	ENSG00000169641	leucine zipper protein 1	1
249	ENSG00000128342	LIF interleukin 6 family cytokine	22
274	ENSG00000050405	LIM domain and actin binding 1	12
70	ENSG00000145012	LIM domain containing preferred translocation partner in lipoma	3
161	ENSG00000148346	lipocalin 2	9
219	ENSG00000189067	lipopolysaccharide induced TNF factor	16
359	ENSG00000131899	LLGL scribble cell polarity complex component 1	17
117	ENSG00000233237	long intergenic non-protein coding RNA 472	6
370	ENSG00000227036	long intergenic non-protein coding RNA 511	17
380	ENSG00000261824	long intergenic non-protein coding RNA 662	19
371	ENSG00000227036	long intergenic non-protein coding RNA 673	17
67	ENSG00000240024	long intergenic non-protein coding RNA 888	3
153	ENSG00000168672	LRAT domain containing 2	8
154	ENSG00000160932	lymphocyte antigen 6 family member E	8
193	ENSG00000136167	lymphocyte cytosolic protein 1	13
20	ENSG00000004487	lysine demethylase 1A	1
44	ENSG00000172086	lysine rich coiled-coil 1	2
32	ENSG00000123684	lysophosphatidylglycerol acyltransferase 1	1
26	ENSG00000011009	lysophospholipase 2	1
199	ENSG00000185896	lysosomal associated membrane protein 1	13
262	ENSG00000005893	lysosomal associated membrane protein 2	X
40	ENSG00000143669	lysosomal trafficking regulator	1
258	ENSG00000138131	lysyl oxidase like 4	10
230	ENSG00000197063	MAF bZIP transcription factor G	17
110	ENSG00000234745	major histocompatibility complex, class I, B	6
108	ENSG00000204642	major histocompatibility complex, class I, F	6
150	ENSG00000147676	mal, T cell differentiation protein 2	8
181	ENSG00000003056	mannose-6-phosphate receptor, cation dependent	12
77	ENSG00000079277	MAPK interacting serine/threonine kinase 1	1
177	ENSG00000196611	matrix metallopeptidase 1	11
197	ENSG00000126217	MCF.2 cell line derived transforming sequence like	13
60	ENSG00000159479	mediator complex subunit 8	1
144	ENSG00000081026	membrane associated guanylate kinase, WW and PDZ domain containing 3	1
132	ENSG00000105976	MET proto-oncogene, receptor tyrosine kinase	7
340	ENSG00000169715	metallothionein 1E	16
378	ENSG00000071655	methyl-CpG binding domain protein 3	19
37	ENSG00000154305	MIA SH3 domain ER export factor 3	1
139	ENSG00000129422	microtubule associated scaffold protein 1	8
91	ENSG00000069020	microtubule associated serine/threonine kinase family member 4	5
14	ENSG00000125462	MIR9-1 host gene	1
346	ENSG00000167842	MIS12 kinetochore complex component	17

90	ENSG00000116221	mitochondrial ribosomal protein L37	1
259	ENSG00000055950	mitochondrial ribosomal protein L43	10
12	ENSG00000116688	mitofusin 2	1
304	ENSG00000168175	mitogen-activated protein kinase 1 interacting protein 1 like	14
308	ENSG00000156030	mitotic deacetylase associated SANT domain protein	14
375	ENSG00000099812	mitotic spindle positioning	19
208	ENSG00000080823	MOK protein kinase	14
16	ENSG00000053372	MRT4 homolog, ribosome maturation factor	1
2	ENSG00000055732	mucolipin 3	1
93	ENSG00000162601	Myb like, SWIRM and MPN domains 1	1
47	ENSG00000214114	MYC binding protein	1
361	ENSG00000278259	myosin XIX	17
102	ENSG00000260604	NA	NA
88	ENSG00000248370	NA	NA
195	ENSG00000275989	NA	NA
196	ENSG00000225760	NA	NA
198	ENSG00000235280	NA	NA
225	ENSG00000263934	NA	NA
56	ENSG00000228192	NA	NA
162	ENSG00000275131	NA	NA
249	ENSG00000273760	NA	NA
355	ENSG00000179277	NA	NA
356	ENSG00000276855	NA	NA
388	ENSG00000198618	NA	NA
392	ENSG00000210196	NA	NA
140	ENSG00000104763	N-acylsphingosine amidohydrolase 1	8
83	ENSG00000078618	nardilysin convertase	1
282	ENSG00000196531	nascent polypeptide associated complex subunit alpha	12
87	ENSG00000058804	NDC1 transmembrane nucleoporin	1
16	ENSG00000143217	nectin cell adhesion molecule 4	1
98	ENSG00000145911	NEDD4 binding protein 3	5
352	ENSG00000065320	netrin 1	17
105	ENSG00000111859	neural precursor cell expressed, developmentally down-regulated 9	6
112	ENSG00000204386	neuraminidase 1	6
163	ENSG00000107130	neuronal calcium sensor 1	9
163	ENSG00000135842	niban apoptosis regulator 1	1
160	ENSG00000136830	niban apoptosis regulator 2	9
311	ENSG00000119638	NIMA related kinase 9	14
148	ENSG00000104361	NIPA like domain containing 2	8
136	ENSG00000164867	nitric oxide synthase 3	7
169	ENSG00000229544	NK1 homeobox 2	10
221	ENSG00000140853	NLR family CARD domain containing 5	16
347	ENSG00000091592	NLR family pyrin domain containing 1	17
218	ENSG00000103202	NME/NM23 nucleoside diphosphate kinase 4	16
186	ENSG00000037474	NOP2/Sun RNA methyltransferase 2	5
76	ENSG00000117481	NOP2/Sun RNA methyltransferase 4	1
160	ENSG00000134250	notch receptor 2	1
148	ENSG00000213281	NRAS proto-oncogene, GTPase	1
319	ENSG00000119720	NRDE-2, necessary for RNA interference, domain containing	14
70	ENSG00000132780	nuclear autoantigenic sperm protein	1
243	ENSG00000124151	nuclear receptor coactivator 3	20
123	ENSG00000111912	nuclear receptor coactivator 7	6
93	ENSG00000175745	nuclear receptor subfamily 2 group F member 1	5
281	ENSG00000139579	nucleic acid binding protein 2	12
260	ENSG00000166197	nucleolar and coiled-body phosphoprotein 1	10
7	ENSG00000162408	nucleolar protein 9	1
260	ENSG00000186462	nucleosome assembly protein 1 like 2	X
251	ENSG00000226328	NUP50 divergent transcript	22
178	ENSG00000124006	obscurin like cytoskeletal adaptor 1	2
84	ENSG00000085840	origin recognition complex subunit 1	1
176	ENSG00000115942	origin recognition complex subunit 2	2
121	ENSG00000081087	osteoclastogenesis associated transmembrane protein 1	6
146	ENSG00000164823	oxidative stress induced growth inhibitor family member 2	8

182	ENSG00000173391	oxidized low density lipoprotein receptor 1	12
228	ENSG00000242265	paternally expressed 10	7
243	ENSG00000164985	PC4 and SFRS1 interacting protein 1	9
257	ENSG00000107438	PDZ and LIM domain 1	10
43	ENSG00000162517	penta-EF-hand domain containing 1	1
220	ENSG00000196262	peptidylprolyl isomerase A	7
68	ENSG00000117450	peroxiredoxin 1	1
263	ENSG00000165672	peroxiredoxin 3	10
252	ENSG00000186951	peroxisome proliferator activated receptor alpha	22
299	ENSG00000247077	PGAM family member 5, mitochondrial serine/threonine protein phosphatase	12
391	ENSG00000177096	PH domain containing endocytic trafficking adaptor 2	22
108	ENSG00000142892	phosphatidylinositol glycan anchor biosynthesis class K	1
362	ENSG00000276293	phosphatidylinositol-5-phosphate 4-kinase type 2 beta	17
210	ENSG00000179364	phosphofurin acidic cluster sorting protein 2	14
97	ENSG00000079739	phosphoglucomutase 1	1
159	ENSG00000092621	phosphoglycerate dehydrogenase	1
53	ENSG00000127125	phosphopantothenoylcysteine synthetase	1
253	ENSG00000198355	Pim-3 proto-oncogene, serine/threonine kinase	22
25	ENSG00000057757	PITH domain containing 1	1
207	ENSG00000118495	PLAG1 like zinc finger 1	6
209	ENSG00000197461	platelet derived growth factor subunit A	7
240	ENSG00000166289	pleckstrin homology and FYVE domain containing 1	19
306	ENSG00000126822	pleckstrin homology and RhoGEF domain containing G3	14
186	ENSG00000139289	pleckstrin homology like domain family A member 1	12
17	ENSG00000143157	pogo transposable element derived with KRAB domain	1
333	ENSG00000090060	poly(A) polymerase alpha	14
28	ENSG00000162896	polymeric immunoglobulin receptor	1
47	ENSG00000136542	polypeptide N-acetylgalactosaminyltransferase 5	2
275	ENSG00000139629	polypeptide N-acetylgalactosaminyltransferase 6	12
390	ENSG00000157551	potassium inwardly rectifying channel subfamily J member 15	21
85	ENSG00000134748	pre-mRNA processing factor 38A	1
131	ENSG00000134186	pre-mRNA processing factor 38B	1
285	ENSG00000177425	pro-apoptotic WT1 regulator	12
344	ENSG00000108518	profilin 1	17
15	ENSG00000162734	proliferation and apoptosis adaptor protein 15	1
137	ENSG00000134222	proline and serine rich coiled-coil 1	1
27	ENSG00000189266	proline rich nuclear receptor coactivator 2	1
55	ENSG00000117385	prolyl 3-hydroxylase 1	1
92	ENSG00000169174	proprotein convertase subtilisin/kexin type 9	1
162	ENSG00000148344	prostaglandin E synthase	9
152	ENSG00000134247	prostaglandin F2 receptor inhibitor	1
247	ENSG00000095303	prostaglandin-endoperoxide synthase 1	9
343	ENSG00000142507	proteasome 20S subunit beta 6	17
318	ENSG00000100764	proteasome 26S subunit, ATPase 1	14
112	ENSG00000142875	protein kinase cAMP-activated catalytic subunit beta	1
180	ENSG00000114302	protein kinase cAMP-dependent type II regulatory subunit alpha	3
235	ENSG00000185900	protein O-mannose kinase	8
26	ENSG00000077157	protein phosphatase 1 regulatory subunit 12B	1
256	ENSG00000119938	protein phosphatase 1 regulatory subunit 3C	10
34	ENSG00000117751	protein phosphatase 1 regulatory subunit 8	1
33	ENSG00000066027	protein phosphatase 2 regulatory subunit B'alpha	1
206	ENSG00000078304	protein phosphatase 2 regulatory subunit B'gamma	14
321	ENSG00000100796	protein phosphatase 4 regulatory subunit 3A	14
124	ENSG00000152894	protein tyrosine phosphatase receptor type K	6
271	ENSG00000129317	pseudouridine synthase 7 like	12
257	ENSG00000067992	pyruvate dehydrogenase kinase 3	X
147	ENSG00000164951	pyruvate dehyrogenase phosphatase catalytic subunit 1	8
107	ENSG00000137955	Rab geranylgeranyltransferase subunit beta	1
27	ENSG00000117280	RAB29, member RAS oncogene family	1
372	ENSG00000168461	RAB31, member RAS oncogene family	18
61	ENSG00000075785	RAB7A, member RAS oncogene family	3
255	ENSG00000123595	RAB9A, member RAS oncogene family	X
18	ENSG00000227373	RABGAP1L divergent transcript	1

239	ENSG00000137040	RAN binding protein 6	9
298	ENSG00000132341	RAN, member RAS oncogene family	12
268	ENSG00000173156	ras homolog family member D	11
137	ENSG00000106615	Ras homolog, mTORC1 binding	7
170	ENSG00000144118	RAS like proto-oncogene B	2
12	ENSG00000143622	Ras like without CAAX 1	1
119	ENSG00000025039	Ras related GTP binding D	6
118	ENSG00000213516	RBMX like 1	1
194	ENSG00000136144	RCC1 and BTB domain containing protein 1	13
341	ENSG00000183688	refilin B	17
37	ENSG00000180198	regulator of chromosome condensation 1	1
15	ENSG00000179051	regulator of chromosome condensation 2	1
73	ENSG00000159788	regulator of G protein signaling 12	4
35	ENSG00000117748	replication protein A2	1
5	ENSG00000157916	retention in endoplasmic reticulum sorting receptor 1	1
65	ENSG00000118849	retinoic acid receptor responder 1	3
122	ENSG00000137962	Rho GTPase activating protein 29	1
166	ENSG00000114993	rhotekin	2
200	ENSG00000165799	ribonuclease A family member 7	14
363	ENSG00000108298	ribosomal protein L19	17
6	ENSG00000116251	ribosomal protein L22	1
193	ENSG00000145592	ribosomal protein L37	5
280	ENSG00000229117	ribosomal protein L41	12
295	ENSG00000089157	ribosomal protein lateral stalk subunit P0	12
377	ENSG00000115268	ribosomal protein S15	19
63	ENSG00000142937	ribosomal protein S8	1
238	ENSG00000107036	RIC1 homolog, RAB6A GEF complex partner 1	9
81	ENSG00000123091	ring finger protein 11	1
171	ENSG00000153250	RNA binding motif single stranded interacting protein 1	2
350	ENSG00000181222	RNA polymerase II subunit A	17
145	ENSG00000081019	round spermatid basic protein 1	1
9	ENSG00000197956	S100 calcium binding protein A6	1
8	ENSG00000163220	S100 calcium binding protein A9	1
74	ENSG00000163993	S100 calcium binding protein P	4
161	ENSG00000265808	SEC22 homolog B, vesicle trafficking protein	1
242	ENSG00000124107	secretory leukocyte peptidase inhibitor	20
75	ENSG00000091490	SEL1L family member 3	4
223	ENSG00000075213	semaphorin 3A	7
217	ENSG00000122545	septin 7	7
99	ENSG00000161011	sequestosome 1	5
28	ENSG00000188529	serine and arginine rich splicing factor 10	1
104	ENSG00000116754	serine and arginine rich splicing factor 11	1
40	ENSG00000116350	serine and arginine rich splicing factor 4	1
270	ENSG00000150687	serine protease 23	11
376	ENSG00000118046	serine/threonine kinase 11	19
101	ENSG00000170542	serpin family B member 9	6
100	ENSG00000142864	SERPINE1 mRNA binding protein 1	1
31	ENSG00000082497	SERTA domain containing 4	1
135	ENSG00000031698	seryl-tRNA synthetase 1	1
205	ENSG00000183576	SET domain containing 3, actin histidine methyltransferase	14
262	ENSG00000187164	shootin 1	10
283	ENSG00000166888	signal transducer and activator of transcription 6	12
266	ENSG00000213445	signal-induced proliferation-associated 1	11
113	ENSG00000186577	small integral membrane protein 29	6
41	ENSG00000060688	small nuclear ribonucleoprotein U5 subunit 40	1
242	ENSG00000164975	small nuclear RNA activating complex polypeptide 3	9
214	ENSG00000228649	small nucleolar RNA host gene 26	7
357	ENSG00000175061	small nucleolar RNA host gene 29	17
38	ENSG00000242125	small nucleolar RNA host gene 3	1
39	ENSG00000200087	small nucleolar RNA, H/ACA box 73B	1
192	ENSG00000079215	solute carrier family 1 member 3	5
143	ENSG00000155380	solute carrier family 16 member 1	1
180	ENSG00000059804	solute carrier family 2 member 3	12

202	ENSG00000183032	solute carrier family 25 member 21	14
290	ENSG00000075415	solute carrier family 25 member 3	12
128	ENSG00000162695	solute carrier family 30 member 7	1
126	ENSG00000117620	solute carrier family 35 member A3	1
106	ENSG00000137968	solute carrier family 44 member 5	1
389	ENSG00000198743	solute carrier family 5 member 3	21
57	ENSG00000131389	solute carrier family 6 member 6	3
83	ENSG00000151012	solute carrier family 7 member 11	4
230	ENSG00000120896	sorbin and SH3 domain containing 3	8
168	ENSG00000198142	sosondowah ankyrin repeat domain family member C	2
229	ENSG00000008294	sperm associated antigen 9	17
191	ENSG00000145604	S-phase kinase associated protein 2	5
232	ENSG00000134278	spire type actin nucleation factor 1	18
30	ENSG00000117632	stathmin 1	1
247	ENSG00000197457	stathmin 3	20
130	ENSG00000127954	STEAP4 metalloreductase	7
187	ENSG00000145545	steroid 5 alpha-reductase 1	5
246	ENSG00000148175	stomatin	9
315	ENSG00000140022	stonin 2	14
32	ENSG00000175793	stratifin	1
190	ENSG00000113387	SUB1 regulator of transcription	5
387	ENSG00000196562	sulfatase 2	20
53	ENSG00000144455	sulfatase modifying factor 1	3
288	ENSG00000120833	suppressor of cytokine signaling 2	12
14	ENSG00000055070	SUZ RNA binding domain containing 1	1
176	ENSG00000137501	synaptotagmin like 2	11
171	ENSG00000166900	syntaxin 3	11
353	ENSG00000133028	synthesis of cytochrome C oxidase 1	17
67	ENSG00000132773	target of EGR1, exonuclease	1
134	ENSG00000197780	TATA-box binding protein associated factor 13	1
45	ENSG00000084652	taxilin alpha	1
66	ENSG00000177565	TBL1X receptor 1	3
254	ENSG00000108219	tetraspanin 14	10
183	ENSG00000168785	tetraspanin 5	4
316	ENSG00000165533	tetratricopeptide repeat domain 8	14
231	ENSG00000177426	TGFB induced factor homeobox 1	18
175	ENSG00000137492	THAP domain containing 12	11
4	ENSG00000265972	thioredoxin interacting protein	1
335	ENSG00000137801	thrombospondin 1	15
128	ENSG00000005108	thrombospondin type 1 domain containing 7A	7
292	ENSG00000139372	thymine DNA glycosylase	12
254	ENSG00000205542	thymosin beta 4 X-linked	X
322	ENSG00000100815	thyroid hormone receptor interactor 11	14
233	ENSG00000223573	TINCR ubiquitin domain containing	19
209	ENSG00000185215	TNF alpha induced protein 2	14
115	ENSG00000146072	TNF receptor superfamily member 21	6
170	ENSG00000078902	toll interacting protein	11
89	ENSG00000116205	transcription elongation factor A N-terminal and central domain containing 2	1
251	ENSG00000108064	transcription factor A, mitochondrial	10
153	ENSG00000116830	transcription termination factor 2	1
228	ENSG00000141232	transducer of ERBB2, 1	17
35	ENSG00000092969	transforming growth factor beta 2	1
195	ENSG00000120708	transforming growth factor beta induced	5
360	ENSG00000171928	trans-golgi network vesicle protein 23 homolog B	17
39	ENSG00000173726	translocase of outer mitochondrial membrane 20	1
213	ENSG00000196683	translocase of outer mitochondrial membrane 7	7
63	ENSG00000169908	transmembrane 4 L six family member 1	3
289	ENSG00000057704	transmembrane and coiled-coil domain family 3	12
286	ENSG00000139324	transmembrane O-mannosyltransferase targeting cadherins 3	12
312	ENSG00000170348	transmembrane p24 trafficking protein 10	14
71	ENSG00000168936	transmembrane protein 129, E3 ubiquitin ligase	4
172	ENSG00000006118	transmembrane protein 132A	11
173	ENSG00000179292	transmembrane protein 151A	11

294	ENSG00000151135	transmembrane protein 263	12
183	ENSG00000165685	transmembrane protein 52B	12
72	ENSG00000159596	transmembrane protein 69	1
178	ENSG00000137648	transmembrane serine protease 4	11
7	ENSG00000182898	trichohyalin like 1	1
354	ENSG00000221926	tripartite motif containing 16	17
109	ENSG00000204616	tripartite motif containing 31	6
147	ENSG00000197323	tripartite motif containing 33	1
87	ENSG00000179046	tripartite motif family like 2	4
49	ENSG00000043514	tRNA isopentenyltransferase 1	1
23	ENSG00000198860	tRNA splicing endonuclease subunit 15	1
105	ENSG00000162623	tRNA-yW synthesizing protein 3 homolog	1
156	ENSG00000116874	tryptophanyl tRNA synthetase 2, mitochondrial	1
301	ENSG00000102804	TSC22 domain family member 1	13
127	ENSG00000130338	TUB like protein 4	6
273	ENSG00000123416	tubulin alpha 1b	12
42	ENSG00000142910	tubulointerstitial nephritis antigen like 1	1
351	ENSG00000141510	tumor protein p53	17
272	ENSG00000151239	twinfilin actin binding protein 1	12
317	ENSG00000042088	tyrosyl-DNA phosphodiesterase 1	14
75	ENSG00000173660	ubiquinol-cytochrome c reductase hinge protein	1
342	ENSG00000132388	ubiquitin conjugating enzyme E2 G1	17
339	ENSG00000103353	ubiquitin family domain containing 1	16
240	ENSG00000147854	ubiquitin like with PHD and ring finger domains 2	9
327	ENSG00000012963	ubiquitin protein ligase E3 component n-recognin 7	14
95	ENSG00000162607	ubiquitin specific peptidase 1	1
110	ENSG00000077254	ubiquitin specific peptidase 33	1
237	ENSG00000179913	UDP-GlcNAc:betaGal beta-1,3-N-acetylglucosaminyltransferase 3	19
165	ENSG00000197355	UDP-N-acetylglucosamine pyrophosphorylase 1 like 1	9
338	ENSG00000140553	unc-45 myosin chaperone A	15
54	ENSG00000144455	uncharacterized LOC100130207	3
144	ENSG00000237807	uncharacterized LOC100507516	8
116	ENSG00000231683	uncharacterized LOC101927136	6
381	ENSG00000267575	uncharacterized LOC101927151	19
225	ENSG00000105793	uncharacterized LOC101927446	7
46	ENSG00000286081	uncharacterized LOC105373682	2
49	ENSG00000233654	uncharacterized LOC105376748	2
65	ENSG00000126088	uroporphyrinogen decarboxylase	1
174	ENSG00000198382	UV radiation resistance associated	11
150	ENSG00000173218	VANGL planar cell polarity protein 1	1
130	ENSG00000134215	vav guanine nucleotide exchange factor 3	1
64	ENSG00000197415	ventricular zone expressed PH domain containing 1	3
226	ENSG00000108828	vesicle amine transport 1	17
8	ENSG00000049245	vesicle associated membrane protein 3	1
263	ENSG00000102243	vestigial like family member 1	X
232	ENSG00000078668	voltage dependent anion channel 3	8
334	ENSG00000100749	VRK serine/threonine kinase 1	14
157	ENSG00000231365	WARS2 antisense RNA 1	1
33	ENSG00000158195	WASP family member 2	1
191	ENSG00000176871	WD repeat and SOCS box containing 2	12
177	ENSG00000138442	WD repeat domain 12	2
155	ENSG00000065183	WD repeat domain 3	1
140	ENSG00000116455	WD repeat domain 77	1
38	ENSG00000143816	Wnt family member 9A	1
54	ENSG00000065978	Y-box binding protein 1	1
259	ENSG00000181704	Yip1 domain family member 6	X
222	ENSG00000184939	ZFP90 zinc finger protein	16
229	ENSG00000105939	zinc finger CCCH-type containing, antiviral 1	7
117	ENSG00000117174	zinc finger HIT-type containing 6	1
107	ENSG00000197279	zinc finger protein 165	6
244	ENSG00000171940	zinc finger protein 217	20
155	ENSG00000196922	zinc finger protein 252, pseudogene	8
300	ENSG00000090612	zinc finger protein 268	12

118	ENSG00000188994	zinc finger protein 292	6
248	ENSG00000265763	zinc finger protein 488	10
119	ENSG00000122482	zinc finger protein 644	1
6	ENSG00000143373	zinc finger protein 687	1
158	ENSG00000143067	zinc finger protein 697	1
145	ENSG00000164684	zinc finger protein 704	8
134	ENSG00000048405	zinc finger protein 800	7
168	ENSG00000215146	zinc finger protein 91 pseudogene	10
239	ENSG00000184635	zinc finger protein 93	19
109	ENSG00000036549	zinc finger ZZ-type containing 3	1
51	ENSG00000084073	zinc metallopeptidase STE24	1
250	ENSG00000122952	ZW10 interacting kinetochore protein	10
Upregulated			
Downregulated:			

Table S5. Deregulated genes resulting from RNA-seq in the CCL-138-R cell line compared to the parental line (CCL-138). A total of 68 downregulated genes and 154 upregulated genes were found. Green indicates downregulated genes and orange indicates upregulated genes.

Sample No.	Gene Code	Gene name	Chromosome
102	ENSG00000089127	2'-5'-oligoadenylate synthetase 1	12
104	ENSG00000111335	2'-5'-oligoadenylate synthetase 2	12
103	ENSG00000111331	2'-5'-oligoadenylate synthetase 3	12
105	ENSG00000135114	2'-5'-oligoadenylate synthetase like	12
63	ENSG00000140350	acidic nuclear phosphoprotein 32 family member A	15
42	ENSG00000169067	actin beta like 2	5
58	ENSG00000153294	adhesion G protein-coupled receptor F4	6
18	ENSG00000071073	alpha-1,3-mannosyl-glycoprotein 4-beta-N-acetylglucosaminyltransferase A	2
125	ENSG00000141279	aminopeptidase puromycin sensitive	17
92	ENSG00000166016	ankyrin repeat and BTB domain containing 2	11
135	ENSG00000130203	apolipoprotein E	19
149	ENSG00000100342	apolipoprotein L1	22
12	ENSG00000180801	arylsulfatase family member J	4
4	ENSG00000066279	assembly factor for spindle microtubules	1
66	ENSG00000152234	ATP synthase F1 subunit alpha	18
24	ENSG00000144476	atypical chemokine receptor 3	2
97	ENSG00000023445	baculoviral IAP repeat containing 3	11
95	ENSG00000168062	basic leucine zipper ATF-like transcription factor 2	11
71	ENSG00000172331	bisphosphoglycerate mutase	7
134	ENSG00000130303	bone marrow stromal cell antigen 2	19
136	ENSG00000125845	bone morphogenetic protein 2	20
141	ENSG00000064787	brain enriched myelin associated protein 1	20
56	ENSG00000060982	branched chain amino acid transaminase 1	12
16	ENSG00000113361	cadherin 6	5
41	ENSG00000151882	C-C motif chemokine ligand 28	5
60	ENSG00000272398	CD24 molecule	6
45	ENSG00000019582	CD74 molecule	5
1	ENSG00000117399	cell division cycle 20	1
5	ENSG00000117724	centromere protein F	1
58	ENSG00000151849	centromere protein J	13
15	ENSG00000150753	chaperonin containing TCP1 subunit 5	5
154	ENSG00000155962	chloride intracellular channel 2	X
57	ENSG00000112782	chloride intracellular channel 5	6
54	ENSG00000243649	complement factor B	6

80	ENSG00000048740	CUGBP Elav-like family member 2	10
34	ENSG00000169245	C-X-C motif chemokine ligand 10	4
35	ENSG00000169248	C-X-C motif chemokine ligand 11	4
18	ENSG00000134057	cyclin B1	5
13	ENSG00000134326	cytidine/uridine monophosphate kinase 2	2
52	ENSG00000006634	DBF4 zinc finger	7
39	ENSG00000181381	DExD/H-box 60 like	4
38	ENSG00000137628	DExD/H-box helicase 60	4
37	ENSG00000070190	dual adaptor of phosphotyrosine and 3-phosphoinositides 1	4
12	ENSG00000143507	dual specificity phosphatase 10	1
7	ENSG00000115380	EGF containing fibulin extracellular matrix protein 1	2
111	ENSG00000129521	egl-9 family hypoxia inducible factor 3	14
47	ENSG00000078401	endothelin 1	6
16	ENSG00000055332	eukaryotic translation initiation factor 2 alpha kinase 2	2
23	ENSG00000115414	fibronectin 1	2
113	ENSG00000185070	fibronectin leucine rich transmembrane protein 2	14
137	ENSG00000125848	fibronectin leucine rich transmembrane protein 3	20
40	ENSG00000082074	FYN binding protein 1	5
130	ENSG00000108679	galectin 3 binding protein	17
	ENSEMBL	GENENAME	CHR
	ENSEMBL	GENENAME	CHR
54	ENSG00000148672	glutamate dehydrogenase 1	10
43	ENSG00000173221	glutaredoxin	5
65	ENSG00000136235	glycoprotein nmb	7
124	ENSG00000030582	granulin precursor	17
8	ENSG00000117228	guanylate binding protein 1	1
26	ENSG00000187837	H1.2 linker histone, cluster member	6
32	ENSG00000124575	H1.3 linker histone, cluster member	6
28	ENSG00000168298	H1.4 linker histone, cluster member	6
48	ENSG00000184357	H1.5 linker histone, cluster member	6
38	ENSG00000196787	H2A clustered histone 11	6
40	ENSG00000274997	H2A clustered histone 12	6
42	ENSG00000196747	H2A clustered histone 13	6
44	ENSG00000276368	H2A clustered histone 14	6
23	ENSG00000278463	H2A clustered histone 4	6
31	ENSG00000277075	H2A clustered histone 8	6
36	ENSG00000278588	H2B clustered histone 10	6
37	ENSG00000124635	H2B clustered histone 11	6
41	ENSG00000185130	H2B clustered histone 13	6
45	ENSG00000273703	H2B clustered histone 14	6
3	ENSG00000203814	H2B clustered histone 18	1
24	ENSG00000276410	H2B clustered histone 3	6
29	ENSG00000274290	H2B clustered histone 6	6
34	ENSG00000275713	H2B clustered histone 9	6
19	ENSG00000275714	H3 clustered histone 1	6
43	ENSG00000278828	H3 clustered histone 10	6
49	ENSG00000275379	H3 clustered histone 11	6
50	ENSG00000197153	H3 clustered histone 12	6
22	ENSG00000286522	H3 clustered histone 2	6
25	ENSG00000287080	H3 clustered histone 3	6
33	ENSG00000277775	H3 clustered histone 7	6
35	ENSG00000273983	H3 clustered histone 8	6
20	ENSG00000278637	H4 clustered histone 1	6
46	ENSG00000197238	H4 clustered histone 11	6
47	ENSG00000273542	H4 clustered histone 12	6
21	ENSG00000278705	H4 clustered histone 2	6
27	ENSG00000197061	H4 clustered histone 3	6
30	ENSG00000277157	H4 clustered histone 4	6
39	ENSG00000276180	H4 clustered histone 9	6
36	ENSG00000138642	HECT and RLD domain containing E3 ubiquitin protein ligase family member 6	4
143	ENSG00000130589	helicase with zinc finger 2	20
133	ENSG00000196684	hematopoietic SH2 domain containing	19
60	ENSG00000189403	high mobility group box 1	13

53	ENSG00000206337	HLA complex P5	6
119	ENSG00000086696	hydroxysteroid 17-beta dehydrogenase 2	16
66	ENSG00000146674	insulin like growth factor binding protein 3	7
132	ENSG00000090339	intercellular adhesion molecule 1	19
114	ENSG00000165949	interferon alpha inducible protein 27	14
2	ENSG00000126709	interferon alpha inducible protein 6	1
62	ENSG00000027697	interferon gamma receptor 1	6
123	ENSG00000068079	interferon induced protein 35	17
7	ENSG00000137965	interferon induced protein 44	1
6	ENSG00000137959	interferon induced protein 44 like	1
84	ENSG00000185745	interferon induced protein with tetratricopeptide repeats 1	10
82	ENSG00000119922	interferon induced protein with tetratricopeptide repeats 2	10
83	ENSG00000119917	interferon induced protein with tetratricopeptide repeats 3	10
85	ENSG00000152778	interferon induced protein with tetratricopeptide repeats 5	10
86	ENSG00000185885	interferon induced transmembrane protein 1	11
87	ENSG00000142089	interferon induced transmembrane protein 3	11
21	ENSG00000115267	interferon induced with helicase C domain 1	2
88	ENSG00000185507	interferon regulatory factor 7	11
115	ENSG00000172183	interferon stimulated exonuclease gene 20	15
19	ENSG00000125538	interleukin 1 beta	2
30	ENSG00000174564	interleukin 20 receptor subunit beta	3
1	ENSG00000187608	ISG15 ubiquitin like modifier	1
151	ENSG00000130487	kelch domain containing 7B	22
122	ENSG00000186832	keratin 16	17
121	ENSG00000108244	keratin 23	17
57	ENSG00000186081	keratin 5	12
79	ENSG00000067082	Kruppel like factor 6	10
147	ENSG00000128342	LIF interleukin 6 family cytokine	22
6	ENSG00000118961	lipid droplet associated hydrolase	2
32	ENSG00000078081	lysosomal associated membrane protein 3	3
52	ENSG00000234745	major histocompatibility complex, class I, B	6
51	ENSG00000204525	major histocompatibility complex, class I, C	6
50	ENSG00000204592	major histocompatibility complex, class I, E	6
49	ENSG00000204642	major histocompatibility complex, class I, F	6
56	ENSG00000231389	major histocompatibility complex, class II, DP alpha 1	6
98	ENSG00000137673	matrix metallopeptidase 7	11
144	ENSG00000157601	MX dynamin like GTPase 1	21
96	ENSG00000172927	myeloma overexpressed	11
46	ENSG00000260604	NA	NA
62	ENSG00000224078	NA	NA
64	ENSG00000259781	NA	NA
108	ENSG00000165795	NDRG family member 2	14
17	ENSG00000163121	neuralized E3 ubiquitin protein ligase 3	2
25	ENSG00000204099	neuraminidase 4	2
118	ENSG00000140853	NLR family CARD domain containing 5	16
150	ENSG00000177989	outer dense fiber of sperm tails 3B	22
5	ENSG00000162366	PDZK1 interacting protein 1	1
93	ENSG00000149090	peptidase domain containing associated with muscle regeneration 1	11
139	ENSG00000124102	peptidase inhibitor 3	20
31	ENSG00000188313	phospholipid scramblase 1	3
81	ENSG00000122861	plasminogen activator, urokinase	10
72	ENSG00000059378	poly(ADP-ribose) polymerase family member 12	7
29	ENSG00000173193	poly(ADP-ribose) polymerase family member 14	3
28	ENSG00000138496	poly(ADP-ribose) polymerase family member 9	3
107	ENSG00000178695	potassium channel tetramerization domain containing 12	13
127	ENSG00000167183	proline rich 15 like	17
78	ENSG00000148344	prostaglandin E synthase	9
74	ENSG00000167653	prostate stem cell antigen	8
53	ENSG00000106278	protein tyrosine phosphatase receptor type Z1	7
106	ENSG00000184226	protocadherin 9	13
117	ENSG00000103485	quinolinate phosphoribosyltransferase	16
14	ENSG00000134321	radical S-adenosyl methionine domain containing 2	2
15	ENSG00000152689	RAS guanyl releasing protein 3	2

33	ENSG00000136514	receptor transporter protein 4	3
69	ENSG00000189056	reelin	7
101	ENSG00000139547	retinol dehydrogenase 16	12
109	ENSG00000165799	ribonuclease A family member 7	14
59	ENSG00000122026	ribosomal protein L21	13
9	ENSG00000168028	ribosomal protein SA	3
11	ENSG00000143556	S100 calcium binding protein A7	1
138	ENSG00000101347	SAM and HD domain containing deoxynucleoside triphosphate triphosphohydrolase 1	20
65	ENSG00000172123	schlafen family member 12	17
51	ENSG00000170381	semaphorin 3E	7
112	ENSG00000165389	serine palmitoyltransferase small subunit A	14
131	ENSG00000197632	serpin family B member 2	18
91	ENSG00000173432	serum amyloid A1	11
90	ENSG00000134339	serum amyloid A2	11
61	ENSG00000118515	serum/glucocorticoid regulated kinase 1	6
2	ENSG00000031698	seryl-tRNA synthetase 1	1
44	ENSG00000169247	SH3 domain and tetratricopeptide repeats 2	5
26	ENSG00000164054	shisa family member 5	3
22	ENSG00000115415	signal transducer and activator of transcription 1	2
100	ENSG00000170581	signal transducer and activator of transcription 2	12
10	ENSG00000241794	small proline rich protein 2A	1
9	ENSG00000163216	small proline rich protein 2D	1
61	ENSG00000102743	solute carrier family 25 member 15	13
73	ENSG00000147606	solute carrier family 26 member 7	8
126	ENSG00000189120	Sp6 transcription factor	17
67	ENSG00000161888	SPC24 component of NDC80 kinetochore complex	19
128	ENSG00000008294	sperm associated antigen 9	17
152	ENSG00000130066	spermidine/spermine N1-acetyltransferase 1	X
10	ENSG00000181449	SRY-box transcription factor 2	3
48	ENSG00000124766	SRY-box transcription factor 4	6
67	ENSG00000205413	sterile alpha motif domain containing 9	7
68	ENSG00000177409	sterile alpha motif domain containing 9 like	7
55	ENSG00000149136	structure specific recognition protein 1	11
64	ENSG00000112096	superoxide dismutase 2	6
8	ENSG00000173705	sushi domain containing 5	3
3	ENSG00000162512	syndecan 3	1
77	ENSG00000041982	tenascin C	9
59	ENSG00000112773	terminal nucleotidyltransferase 5A	6
17	ENSG00000113407	threonyl-tRNA synthetase 1	5
14	ENSG00000071539	thyroid hormone receptor interactor 13	5
129	ENSG00000035862	TIMP metallopeptidase inhibitor 2	17
148	ENSG00000100234	TIMP metallopeptidase inhibitor 3	22
63	ENSG00000118503	TNF alpha induced protein 3	6
76	ENSG00000181634	TNF superfamily member 15	9
70	ENSG00000105967	transcription factor EC	7
13	ENSG00000170006	transmembrane protein 154	4
11	ENSG00000121895	transmembrane protein 156	4
153	ENSG00000147027	transmembrane protein 47	X
145	ENSG00000184012	transmembrane serine protease 2	21
55	ENSG00000168394	transporter 1, ATP binding cassette subfamily B member	6
89	ENSG00000132274	tripartite motif containing 22	11
99	ENSG00000167552	tubulin alpha 1a	12
75	ENSG00000196116	tudor domain containing 7	9
94	ENSG00000156587	ubiquitin conjugating enzyme E2 L6	11
27	ENSG00000182179	ubiquitin like modifier activating enzyme 7	3
146	ENSG00000184979	ubiquitin specific peptidase 18	22
110	ENSG00000251002	uncharacterized LOC105370401	14
20	ENSG00000286081	uncharacterized LOC105373682	2
120	ENSG00000132530	XIAP associated factor 1	17
142	ENSG00000124256	Z-DNA binding protein 1	20
4	ENSG00000163874	zinc finger CCCH-type containing 12A	1
140	ENSG00000124201	zinc finger NFX1-type containing 1	20
116	ENSG00000162078	zymogen granule protein 16B	16

Upregulated genes
Downregulated genes

Table S6. Deregulated proteins in HTB-43-R cells versus parental cells (HTB-43). Adjusted *p*-values and *p*-values are indicated. Fold change (FC) values are indicated. Negative numbers correspond to deregulated proteins and positive numbers to positive proteins.

Swiss-Prot ID	Protein name	p	p (Corr)	Regulation	FC Resistant vs Parental cells
P30041	Peroxiredoxin-6 OS=Homo sapiens GN=PRDX6 PE=1 SV=3 - [PRDX6_HUMAN]	6.39E-05	1.78E-03	up	1.6062549
P23528	Cofilin-1 OS=Homo sapiens GN=CFL1 PE=1 SV=3 - [COF1_HUMAN]	3.79E-03	1.48E-02	down	-1.2341194
P68104	Elongation factor 1-alpha 1 OS=Homo sapiens GN=EEF1A1 PE=1 SV=1 - [EF1A1_HUMAN]	1.88E-02	4.43E-02	down	-1.2412293
P60174	Triosephosphate isomerase OS=Homo sapiens GN=TPI1 PE=1 SV=3 - [TPIS_HUMAN]	2.00E-02	4.65E-02	up	1.1414198
Q06830	Peroxiredoxin-1 OS=Homo sapiens GN=PRDX1 PE=1 SV=1 - [PRDX1_HUMAN]	2.69E-04	3.18E-03	down	-1.4436241
P62937	Peptidyl-prolyl cis-trans isomerase A OS=Homo sapiens GN=PPIA PE=1 SV=2 - [PPIA_HUMAN]	2.92E-03	1.27E-02	down	-1.2447767
P04792	Heat shock protein beta-1 OS=Homo sapiens GN=HSPB1 PE=1 SV=2 - [HSPB1_HUMAN]	4.59E-04	4.06E-03	up	1.5795714
P00558	Phosphoglycerate kinase 1 OS=Homo sapiens GN=PGK1 PE=1 SV=3 - [PGK1_HUMAN]	1.81E-05	1.36E-03	up	1.3082011
P35232	Prohibitin OS=Homo sapiens GN=PHB PE=1 SV=1 - [PHB_HUMAN]	7.32E-03	2.27E-02	up	1.1551746
P09211	Glutathione S-transferase P OS=Homo sapiens GN=GSTP1 PE=1 SV=2 - [GSTP1_HUMAN]	1.70E-03	8.55E-03	up	1.276601
P37108	Signal recognition particle 14 kDa protein OS=Homo sapiens GN=SRP14 PE=1 SV=2 - [SRP14_HUMAN]	1.71E-02	4.12E-02	up	1.1334251
Q99623	Prohibitin-2 OS=Homo sapiens GN=PHB2 PE=1 SV=2 - [PHB2_HUMAN]	9.38E-04	6.11E-03	up	1.202131
P07437	Tubulin beta chain OS=Homo sapiens GN=TUBB PE=1 SV=2 - [TBB5_HUMAN]	1.44E-02	3.66E-02	down	-1.3822523
P09382	Galectin-1 OS=Homo sapiens GN=LGALS1 PE=1 SV=2 - [LEG1_HUMAN]	5.37E-03	1.87E-02	down	-2.1661763
P15559	NAD(P)H dehydrogenase [quinone] 1 OS=Homo sapiens GN=NQO1 PE=1 SV=1 - [NQO1_HUMAN]	4.90E-04	4.30E-03	up	1.6138847
P06733	Alpha-enolase OS=Homo sapiens GN=ENO1 PE=1 SV=2 - [ENOA_HUMAN]	7.00E-03	2.22E-02	down	-1.5024956
P00492	Hypoxanthine-guanine phosphoribosyltransferase OS=Homo sapiens GN=HPRT1 PE=1 SV=2 - [HPRT_HUMAN]	6.43E-03	2.09E-02	up	1.1258961
P60842	Eukaryotic initiation factor 4A-I OS=Homo sapiens GN=EIF4A1 PE=1 SV=1 - [IF4A1_HUMAN]	5.87E-05	1.78E-03	down	-1.4718035
P30050	60S ribosomal protein L12 OS=Homo sapiens GN=RPL12 PE=1 SV=1 - [RL12_HUMAN]	1.51E-03	8.02E-03	down	-1.1765697
P31946	14-3-3 protein beta/alpha OS=Homo sapiens GN=YWHAB PE=1 SV=3 - [1433B_HUMAN]	1.64E-03	8.36E-03	up	1.2718947
P18124	60S ribosomal protein L7 OS=Homo sapiens GN=RPL7 PE=1 SV=1 - [RL7_HUMAN]	4.76E-03	1.70E-02	down	-1.1101823
P31947	14-3-3 protein sigma OS=Homo sapiens GN=SFN PE=1 SV=1 - [1433S_HUMAN]	1.25E-04	2.21E-03	down	-2.0151713
P23284	Peptidyl-prolyl cis-trans isomerase B OS=Homo sapiens GN=PPIB PE=1 SV=2 - [PPIB_HUMAN]	3.77E-04	3.62E-03	up	1.3232296
P00338	L-lactate dehydrogenase A chain OS=Homo sapiens GN=LDHA PE=1 SV=2 - [LDHA_HUMAN]	1.39E-03	7.57E-03	down	-1.2208092

Q14019	Coactosin-like protein OS=Homo sapiens GN=COTL1 PE=1 SV=3 - [COTL1_HUMAN]	7.24E-03	2.25E-02	up	1.5707432
P62258	14-3-3 protein epsilon OS=Homo sapiens GN=YWHAE PE=1 SV=1 - [1433E_HUMAN]	1.52E-02	3.80E-02	down	-1.1411275
P23396	40S ribosomal protein S3 OS=Homo sapiens GN=RPS3 PE=1 SV=2 - [RS3_HUMAN]	6.81E-03	2.18E-02	down	-1.1796442
P07195	L-lactate dehydrogenase B chain OS=Homo sapiens GN=LDHB PE=1 SV=2 - [LDHB_HUMAN]	6.82E-05	1.84E-03	up	1.232984
Q99714	3-hydroxyacyl-CoA dehydrogenase type-2 OS=Homo sapiens GN=HSD17B10 PE=1 SV=3 - [HCD2_HUMAN]	4.16E-03	1.58E-02	up	1.1669333
P07900	Heat shock protein HSP 90-alpha OS=Homo sapiens GN=HSP90AA1 PE=1 SV=5 - [HS90A_HUMAN]	3.92E-03	1.51E-02	up	1.1636283
P08238	Heat shock protein HSP 90-beta OS=Homo sapiens GN=HSP90AB1 PE=1 SV=4 - [HS90B_HUMAN]	8.96E-05	1.93E-03	down	-1.5619411
P07737	Profilin-1 OS=Homo sapiens GN=PFN1 PE=1 SV=2 - [PROF1_HUMAN]	7.13E-04	5.30E-03	down	-1.6706427
Q13885	Tubulin beta-2A chain OS=Homo sapiens GN=TUBB2A PE=1 SV=1 - [TBB2A_HUMAN]	6.21E-03	2.06E-02	down	-1.7487133
Q9BQE3	Tubulin alpha-1C chain OS=Homo sapiens GN=TUBA1C PE=1 SV=1 - [TBA1C_HUMAN]	1.30E-02	3.41E-02	down	-1.1826854
P11142	Heat shock cognate 71 kDa protein OS=Homo sapiens GN=HSPA8 PE=1 SV=1 - [HSP7C_HUMAN]	4.12E-03	1.57E-02	down	-1.1618044
P62701	40S ribosomal protein S4, X isoform OS=Homo sapiens GN=RPS4X PE=1 SV=2 - [RS4X_HUMAN]	3.69E-03	1.46E-02	down	-1.2036382
P07355	Annexin A2 OS=Homo sapiens GN=ANXA2 PE=1 SV=2 - [ANXA2_HUMAN]	1.65E-02	4.03E-02	down	-1.108626
Q99497	Protein DJ-1 OS=Homo sapiens GN=PARK7 PE=1 SV=2 - [PARK7_HUMAN]	1.23E-03	7.10E-03	down	-1.3677577
P00505	Aspartate aminotransferase, mitochondrial OS=Homo sapiens GN=GOT2 PE=1 SV=3 - [AATM_HUMAN]	9.53E-05	1.99E-03	up	1.4605905
P63244	Receptor of activated protein C kinase 1 OS=Homo sapiens GN=RACK1 PE=1 SV=3 - [RACK1_HUMAN]	3.52E-04	3.58E-03	down	-1.1869397
P31949	Protein S100-A11 OS=Homo sapiens GN=S100A11 PE=1 SV=2 - [S10AB_HUMAN]	1.27E-02	3.35E-02	up	1.3337348
P30086	Phosphatidylethanolamine-binding protein 1 OS=Homo sapiens GN=PEBP1 PE=1 SV=3 - [PEBP1_HUMAN]	1.65E-04	2.44E-03	up	1.3983476
P51148	Ras-related protein Rab-5C OS=Homo sapiens GN=RAB5C PE=1 SV=2 - [RAB5C_HUMAN]	1.90E-04	2.68E-03	up	1.3729767
P22392	Nucleoside diphosphate kinase B OS=Homo sapiens GN=NME2 PE=1 SV=1 - [NDKB_HUMAN]	8.64E-04	5.87E-03	up	1.4499636
P0DMV8	Heat shock 70 kDa protein 1A OS=Homo sapiens GN=HSPA1A PE=1 SV=1 - [HS71A_HUMAN]	8.40E-05	1.93E-03	up	1.3490398
P13639	Elongation factor 2 OS=Homo sapiens GN=EEF2 PE=1 SV=4 - [EF2_HUMAN]	3.11E-04	3.39E-03	down	-1.2252377
P50454	Serpin H1 OS=Homo sapiens GN=SERPINH1 PE=1 SV=2 - [SERPH_HUMAN]	1.10E-04	2.10E-03	down	-1.416099
P24534	Elongation factor 1-beta OS=Homo sapiens GN=EEF1B2 PE=1 SV=3 - [EF1B_HUMAN]	6.69E-03	2.15E-02	down	-1.2675023
Q9HB71	Calcyclin-binding protein OS=Homo sapiens GN=CACYBP PE=1 SV=2 - [CYBP_HUMAN]	1.81E-03	8.88E-03	up	1.2186345
O43707	Alpha-actinin-4 OS=Homo sapiens GN=ACTN4 PE=1 SV=2 - [ACTN4_HUMAN]	3.61E-03	1.44E-02	up	1.1556438
P68366	Tubulin alpha-4A chain OS=Homo sapiens GN=TUBA4A PE=1 SV=1 - [TBA4A_HUMAN]	5.78E-05	1.78E-03	down	-1.2423459
Q9Y277	Voltage-dependent anion-selective channel protein 3 OS=Homo sapiens GN=VDAC3 PE=1 SV=1 - [VDAC3_HUMAN]	2.92E-04	3.28E-03	down	-1.5041708
O75874	Isocitrate dehydrogenase [NADP] cytoplasmic OS=Homo sapiens GN=IDH1 PE=1 SV=2 - [IDHC_HUMAN]	2.38E-06	8.34E-04	up	1.7515198
P29692	Elongation factor 1-delta OS=Homo sapiens GN=EEF1D PE=1 SV=5 - [EF1D_HUMAN]	5.81E-03	1.98E-02	down	-1.1551785
Q96KP4	Cytosolic non-specific dipeptidase OS=Homo sapiens GN=CNDP2 PE=1 SV=2 - [CNDP2_HUMAN]	1.35E-04	2.34E-03	up	1.2593963
P51149	Ras-related protein Rab-7a OS=Homo sapiens GN=RAB7A PE=1 SV=1 - [RAB7A_HUMAN]	1.59E-03	8.29E-03	up	1.430655
Q15181	Inorganic pyrophosphatase OS=Homo sapiens GN=PPA1 PE=1 SV=2 - [IPYR_HUMAN]	1.26E-03	7.16E-03	down	-1.2080727
P15880	40S ribosomal protein S2 OS=Homo sapiens GN=RPS2 PE=1 SV=2 - [RS2_HUMAN]	1.95E-02	4.55E-02	down	-1.215076

P46783	40S ribosomal protein S10 OS=Homo sapiens GN=RPS10 PE=1 SV=1 - [RS10_HUMAN]	2.39E-03	1.10E-02	down	-1.2302696
P22626	Heterogeneous nuclear ribonucleoproteins A2/B1 OS=Homo sapiens GN=HNRNPA2B1 PE=1 SV=2 - [ROA2_HUMAN]	2.37E-03	1.09E-02	down	-1.1977986
P61106	Ras-related protein Rab-14 OS=Homo sapiens GN=RAB14 PE=1 SV=4 - [RAB14_HUMAN]	2.73E-03	1.21E-02	up	1.3336896
P15311	Ezrin OS=Homo sapiens GN=EZR PE=1 SV=4 - [EZRI_HUMAN]	1.62E-04	2.44E-03	up	1.450627
P11021	78 kDa glucose-regulated protein OS=Homo sapiens GN=HSPA5 PE=1 SV=2 - [GRP78_HUMAN]	2.86E-04	3.27E-03	up	1.4802543
P61247	40S ribosomal protein S3a OS=Homo sapiens GN=RPS3A PE=1 SV=2 - [RS3A_HUMAN]	2.55E-03	1.15E-02	down	-1.1924746
P26641	Elongation factor 1-gamma OS=Homo sapiens GN=EEF1G PE=1 SV=3 - [EF1G_HUMAN]	1.71E-03	8.55E-03	down	-1.1577983
P30101	Protein disulfide-isomerase A3 OS=Homo sapiens GN=PDIA3 PE=1 SV=4 - [PDIA3_HUMAN]	9.50E-04	6.11E-03	up	1.4193659
Q14247	Src substrate cortactin OS=Homo sapiens GN=CTTN PE=1 SV=2 - [SRC8_HUMAN]	1.97E-02	4.59E-02	down	-1.1920252
Q02543	60S ribosomal protein L18a OS=Homo sapiens GN=RPL18A PE=1 SV=2 - [RL18A_HUMAN]	7.13E-04	5.30E-03	down	-1.1813025
Q02878	60S ribosomal protein L6 OS=Homo sapiens GN=RPL6 PE=1 SV=3 - [RL6_HUMAN]	1.49E-02	3.75E-02	down	-1.150198
P16152	Carbonyl reductase [NADPH] 1 OS=Homo sapiens GN=CBR1 PE=1 SV=3 - [CBR1_HUMAN]	2.03E-02	4.68E-02	up	1.2413833
P49327	Fatty acid synthase OS=Homo sapiens GN=FASN PE=1 SV=3 - [FAS_HUMAN]	1.84E-02	4.35E-02	up	1.1383919
Q01518	Adenylyl cyclase-associated protein 1 OS=Homo sapiens GN=CAP1 PE=1 SV=5 - [CAP1_HUMAN]	7.91E-04	5.60E-03	down	-1.3925996
P13010	X-ray repair cross-complementing protein 5 OS=Homo sapiens GN=XRCC5 PE=1 SV=3 - [XRCC5_HUMAN]	5.67E-04	4.64E-03	down	-1.3123354
P68036	Ubiquitin-conjugating enzyme E2 L3 OS=Homo sapiens GN=UBE2L3 PE=1 SV=1 - [UB2L3_HUMAN]	8.69E-03	2.58E-02	up	1.2318766
P21796	Voltage-dependent anion-selective channel protein 1 OS=Homo sapiens GN=VDAC1 PE=1 SV=2 - [VDAC1_HUMAN]	1.32E-03	7.37E-03	up	1.236651
P62081	40S ribosomal protein S7 OS=Homo sapiens GN=RPS7 PE=1 SV=1 - [RS7_HUMAN]	1.38E-02	3.54E-02	down	-1.2224071
P06576	ATP synthase subunit beta, mitochondrial OS=Homo sapiens GN=ATP5B PE=1 SV=3 - [ATPB_HUMAN]	3.94E-03	1.52E-02	down	-1.2279522
P62424	60S ribosomal protein L7a OS=Homo sapiens GN=RPL7A PE=1 SV=2 - [RL7A_HUMAN]	1.67E-04	2.44E-03	down	-1.2012862
P63241	Eukaryotic translation initiation factor 5A-1 OS=Homo sapiens GN=EIF5A PE=1 SV=2 - [IF5A1_HUMAN]	2.84E-04	3.27E-03	down	-1.6620091
P17174	Aspartate aminotransferase, cytoplasmic OS=Homo sapiens GN=GOT1 PE=1 SV=3 - [AATC_HUMAN]	2.10E-02	4.81E-02	down	-1.1220492
P62820	Ras-related protein Rab-1A OS=Homo sapiens GN=RAB1A PE=1 SV=3 - [RAB1A_HUMAN]	3.44E-03	1.40E-02	up	1.2865559
Q05639	Elongation factor 1-alpha 2 OS=Homo sapiens GN=EEF1A2 PE=1 SV=1 - [EF1A2_HUMAN]	5.03E-07	8.34E-04	up	3.5856824
P61019	Ras-related protein Rab-2A OS=Homo sapiens GN=RAB2A PE=1 SV=1 - [RAB2A_HUMAN]	3.66E-04	3.61E-03	up	1.3084416
P12814	Alpha-actinin-1 OS=Homo sapiens GN=ACTN1 PE=1 SV=2 - [ACTN1_HUMAN]	6.70E-03	2.15E-02	up	1.1193713
P30042	ES1 protein homolog, mitochondrial OS=Homo sapiens GN=C21orf33 PE=1 SV=3 - [ES1_HUMAN]	7.37E-03	2.27E-02	up	1.1812938
P18669	Phosphoglycerate mutase 1 OS=Homo sapiens GN=PGAM1 PE=1 SV=2 - [PGAM1_HUMAN]	5.42E-03	1.88E-02	down	-1.2068174
P48047	ATP synthase subunit O, mitochondrial OS=Homo sapiens GN=ATP5O PE=1 SV=1 - [ATPO_HUMAN]	1.35E-02	3.49E-02	down	-1.2747991
Q16881	Thioredoxin reductase 1, cytoplasmic OS=Homo sapiens GN=TXNRD1 PE=1 SV=3 - [TRXR1_HUMAN]	5.27E-03	1.85E-02	up	1.1081886
P30085	UMP-CMP kinase OS=Homo sapiens GN=CMPK1 PE=1 SV=3 - [KCY_HUMAN]	1.13E-03	6.80E-03	down	-1.6023248
P07237	Protein disulfide-isomerase OS=Homo sapiens GN=P4HB PE=1 SV=3 - [PDIA1_HUMAN]	7.16E-03	2.24E-02	up	1.2946332
P38646	Stress-70 protein, mitochondrial OS=Homo sapiens GN=HSPA9 PE=1 SV=2 - [GRP75_HUMAN]	5.20E-03	1.83E-02	up	1.196175
P11413	Glucose-6-phosphate 1-dehydrogenase OS=Homo sapiens GN=G6PD PE=1 SV=4 - [G6PD_HUMAN]	2.67E-03	1.19E-02	up	1.1954743

P62263	40S ribosomal protein S14 OS=Homo sapiens GN=RPS14 PE=1 SV=3 - [RS14_HUMAN]	1.18E-02	3.19E-02	down	-1.3029432
P27348	14-3-3 protein theta OS=Homo sapiens GN=YWHAQ PE=1 SV=1 - [1433T_HUMAN]	9.39E-03	2.71E-02	up	1.2264786
P08708	40S ribosomal protein S17 OS=Homo sapiens GN=RPS17 PE=1 SV=2 - [RS17_HUMAN]	1.91E-02	4.47E-02	down	-1.2349489
P25705	ATP synthase subunit alpha, mitochondrial OS=Homo sapiens GN=ATP5A1 PE=1 SV=1 - [ATPA_HUMAN]	1.17E-03	6.90E-03	down	-1.2573305
Q06323	Proteasome activator complex subunit 1 OS=Homo sapiens GN=PSME1 PE=1 SV=1 - [PSME1_HUMAN]	1.40E-02	3.58E-02	up	1.2133229
P55072	Transitional endoplasmic reticulum ATPase OS=Homo sapiens GN=VCP PE=1 SV=4 - [TERA_HUMAN]	3.22E-03	1.34E-02	down	-1.1345266
Q13509	Tubulin beta-3 chain OS=Homo sapiens GN=TUBB3 PE=1 SV=2 - [TBB3_HUMAN]	2.35E-03	1.08E-02	down	-1.366184
P12235	ADP/ATP translocase 1 OS=Homo sapiens GN=SLC25A4 PE=1 SV=4 - [ADT1_HUMAN]	2.13E-03	1.01E-02	up	1.2829441
P83731	60S ribosomal protein L24 OS=Homo sapiens GN=RPL24 PE=1 SV=1 - [RL24_HUMAN]	8.73E-04	5.88E-03	down	-1.1736782
P09661	U2 small nuclear ribonucleoprotein A' OS=Homo sapiens GN=SNRPA1 PE=1 SV=2 - [RU2A_HUMAN]	3.31E-03	1.37E-02	down	-1.1250858
P18621	60S ribosomal protein L17 OS=Homo sapiens GN=RPL17 PE=1 SV=3 - [RL17_HUMAN]	5.61E-03	1.93E-02	down	-1.1430866
P22102	Trifunctional purine biosynthetic protein adenosine-3 OS=Homo sapiens GN=GART PE=1 SV=1 - [PUR2_HUMAN]	6.71E-05	1.84E-03	down	-1.5227143
P49588	Alanine--tRNA ligase, cytoplasmic OS=Homo sapiens GN=AARS PE=1 SV=2 - [SYAC_HUMAN]	3.50E-03	1.41E-02	up	1.4299626
Q08257	Quinone oxidoreductase OS=Homo sapiens GN=CRYZ PE=1 SV=1 - [QOR_HUMAN]	1.95E-05	1.36E-03	down	-1.3778034
P02794	Ferritin heavy chain OS=Homo sapiens GN=FTH1 PE=1 SV=2 - [FRIH_HUMAN]	5.66E-04	4.64E-03	up	1.9602916
O60506	Heterogeneous nuclear ribonucleoprotein Q OS=Homo sapiens GN=SYNCRIP PE=1 SV=2 - [HNRPQ_HUMAN]	1.30E-03	7.30E-03	up	1.2307497
Q15907	Ras-related protein Rab-11B OS=Homo sapiens GN=RAB11B PE=1 SV=4 - [RB11B_HUMAN]	3.47E-04	3.55E-03	up	1.2526463
P11586	C-1-tetrahydrofolate synthase, cytoplasmic OS=Homo sapiens GN=MTHFD1 PE=1 SV=3 - [C1TC_HUMAN]	3.78E-04	3.62E-03	down	-1.4721048
P62826	GTP-binding nuclear protein Ran OS=Homo sapiens GN=RAN PE=1 SV=3 - [RAN_HUMAN]	1.23E-02	3.28E-02	down	-1.3588738
P14625	Endoplasmin OS=Homo sapiens GN=HSP90B1 PE=1 SV=1 - [ENPL_HUMAN]	2.15E-05	1.36E-03	up	1.6472217
P27635	60S ribosomal protein L10 OS=Homo sapiens GN=RPL10 PE=1 SV=4 - [RL10_HUMAN]	1.58E-02	3.89E-02	down	-1.1761628
P80188	Neutrophil gelatinase-associated lipocalin OS=Homo sapiens GN=LCN2 PE=1 SV=2 - [NGAL_HUMAN]	2.94E-03	1.28E-02	up	2.7148077
P68431	Histone H3.1 OS=Homo sapiens GN=HIST1H3A PE=1 SV=2 - [H31_HUMAN]	1.11E-02	3.04E-02	down	-1.4482012
P39023	60S ribosomal protein L3 OS=Homo sapiens GN=RPL3 PE=1 SV=2 - [RL3_HUMAN]	3.22E-03	1.34E-02	down	-1.179685
P62917	60S ribosomal protein L8 OS=Homo sapiens GN=RPL8 PE=1 SV=2 - [RL8_HUMAN]	6.32E-03	2.08E-02	down	-1.1288501
P62906	60S ribosomal protein L10a OS=Homo sapiens GN=RPL10A PE=1 SV=2 - [RL10A_HUMAN]	7.02E-03	2.22E-02	down	-1.1210649
P40925	Malate dehydrogenase, cytoplasmic OS=Homo sapiens GN=MDH1 PE=1 SV=4 - [MDHC_HUMAN]	4.25E-04	3.88E-03	up	1.2935199
P46781	40S ribosomal protein S9 OS=Homo sapiens GN=RPS9 PE=1 SV=3 - [RS9_HUMAN]	4.72E-03	1.69E-02	down	-1.1730803
Q16658	Fascin OS=Homo sapiens GN=FSCN1 PE=1 SV=3 - [FSCN1_HUMAN]	1.81E-03	8.88E-03	down	-1.8247709
P62280	40S ribosomal protein S11 OS=Homo sapiens GN=RPS11 PE=1 SV=3 - [RS11_HUMAN]	7.24E-04	5.35E-03	down	-1.2117864
P12956	X-ray repair cross-complementing protein 6 OS=Homo sapiens GN=XRCC6 PE=1 SV=2 - [XRCC6_HUMAN]	9.95E-05	2.01E-03	down	-1.3034099
P17844	Probable ATP-dependent RNA helicase DDX5 OS=Homo sapiens GN=DDX5 PE=1 SV=1 - [DDX5_HUMAN]	2.52E-04	3.07E-03	down	-1.3476697
P61353	60S ribosomal protein L27 OS=Homo sapiens GN=RPL27 PE=1 SV=2 - [RL27_HUMAN]	6.65E-04	5.11E-03	down	-1.1860358
P62266	40S ribosomal protein S23 OS=Homo sapiens GN=RPS23 PE=1 SV=3 - [RS23_HUMAN]	1.35E-02	3.49E-02	down	-1.2439618

P29401	Transketolase OS=Homo sapiens GN=TKT PE=1 SV=3 - [TKT_HUMAN]	4.42E-03	1.64E-02	up	1.203193
P13804	Electron transfer flavoprotein subunit alpha, mitochondrial OS=Homo sapiens GN=ETFA PE=1 SV=1 - [ETFA_HUMAN]	7.37E-06	1.35E-03	up	1.5177015
P05161	Ubiquitin-like protein ISG15 OS=Homo sapiens GN=ISG15 PE=1 SV=5 - [ISG15_HUMAN]	6.08E-05	1.78E-03	down	-2.0635717
Q92841	Probable ATP-dependent RNA helicase DDX17 OS=Homo sapiens GN=DDX17 PE=1 SV=2 - [DDX17_HUMAN]	3.65E-04	3.61E-03	up	1.1654038
P00918	Carbonic anhydrase 2 OS=Homo sapiens GN=CA2 PE=1 SV=2 - [CAH2_HUMAN]	1.44E-02	3.66E-02	up	1.3509647
Q00610	Clathrin heavy chain 1 OS=Homo sapiens GN=CLTC PE=1 SV=5 - [CLH1_HUMAN]	9.59E-04	6.13E-03	up	1.2153449
P62191	26S protease regulatory subunit 4 OS=Homo sapiens GN=PSMC1 PE=1 SV=1 - [PRS4_HUMAN]	6.86E-03	2.19E-02	down	-1.1891428
Q04760	Lactoylglutathione lyase OS=Homo sapiens GN=GLO1 PE=1 SV=4 - [LGUL_HUMAN]	2.11E-02	4.82E-02	up	1.1353526
P43490	Nicotinamide phosphoribosyltransferase OS=Homo sapiens GN=NAMPT PE=1 SV=1 - [NAMPT_HUMAN]	1.84E-06	8.34E-04	up	1.7633975
P26038	Moesin OS=Homo sapiens GN=MSN PE=1 SV=3 - [MOES_HUMAN]	3.04E-03	1.29E-02	down	-1.4272814
O60701	UDP-glucose 6-dehydrogenase OS=Homo sapiens GN=UGDH PE=1 SV=1 - [UGDH_HUMAN]	5.83E-05	1.78E-03	up	1.2146384
P04843	Dolichyl-diphosphooligosaccharide--protein glycosyltransferase subunit 1 OS=Homo sapiens GN=RPN1 PE=1 SV=1 - [RPN1_HUMAN]	1.66E-02	4.04E-02	up	1.1063002
P00568	Adenylate kinase isoenzyme 1 OS=Homo sapiens GN=AK1 PE=1 SV=3 - [KAD1_HUMAN]	1.52E-02	3.80E-02	up	1.2606336
P17980	26S protease regulatory subunit 6A OS=Homo sapiens GN=PSMC3 PE=1 SV=3 - [PRS6A_HUMAN]	4.27E-03	1.61E-02	down	-1.1797079
P46779	60S ribosomal protein L28 OS=Homo sapiens GN=RPL28 PE=1 SV=3 - [RL28_HUMAN]	1.52E-02	3.80E-02	down	-1.2236639
P47895	Aldehyde dehydrogenase family 1 member A3 OS=Homo sapiens GN=ALDH1A3 PE=1 SV=2 - [AL1A3_HUMAN]	2.31E-03	1.07E-02	down	-1.1389194
P13667	Protein disulfide-isomerase A4 OS=Homo sapiens GN=PDIA4 PE=1 SV=2 - [PDIA4_HUMAN]	6.37E-05	1.78E-03	up	1.4710776
Q9NX63	MICOS complex subunit MIC19 OS=Homo sapiens GN=CHCHD3 PE=1 SV=1 - [MIC19_HUMAN]	1.67E-03	8.47E-03	up	1.286661
P61289	Proteasome activator complex subunit 3 OS=Homo sapiens GN=PSME3 PE=1 SV=1 - [PSME3_HUMAN]	2.30E-03	1.07E-02	down	-1.100153
P31939	Bifunctional purine biosynthesis protein PURH OS=Homo sapiens GN=ATIC PE=1 SV=3 - [PUR9_HUMAN]	1.45E-05	1.36E-03	down	-1.2837373
Q96C19	EF-hand domain-containing protein D2 OS=Homo sapiens GN=EFHD2 PE=1 SV=1 - [EFHD2_HUMAN]	5.46E-05	1.78E-03	down	-2.5403726
P14923	Junction plakoglobin OS=Homo sapiens GN=JUP PE=1 SV=3 - [PLAK_HUMAN]	3.41E-03	1.40E-02	up	1.227592
Q15084	Protein disulfide-isomerase A6 OS=Homo sapiens GN=PDIA6 PE=1 SV=1 - [PDIA6_HUMAN]	4.85E-05	1.78E-03	up	1.4844995
P36578	60S ribosomal protein L4 OS=Homo sapiens GN=RPL4 PE=1 SV=5 - [RL4_HUMAN]	6.73E-03	2.16E-02	down	-1.1735872
P00387	NADH-cytochrome b5 reductase 3 OS=Homo sapiens GN=CYB5R3 PE=1 SV=3 - [NB5R3_HUMAN]	3.01E-03	1.28E-02	up	1.1891589
O43175	D-3-phosphoglycerate dehydrogenase OS=Homo sapiens GN=PHGDH PE=1 SV=4 - [SERA_HUMAN]	1.49E-04	2.44E-03	down	-2.1595984
P51991	Heterogeneous nuclear ribonucleoprotein A3 OS=Homo sapiens GN=HNRNPA3 PE=1 SV=2 - [ROA3_HUMAN]	1.40E-02	3.58E-02	down	-1.2101227
Q14240	Eukaryotic initiation factor 4A-II OS=Homo sapiens GN=EIF4A2 PE=1 SV=2 - [IF4A2_HUMAN]	9.63E-03	2.75E-02	up	1.2813967
Q9UL46	Proteasome activator complex subunit 2 OS=Homo sapiens GN=PSME2 PE=1 SV=4 - [PSME2_HUMAN]	9.75E-03	2.77E-02	up	1.209638
P40939	Trifunctional enzyme subunit alpha, mitochondrial OS=Homo sapiens GN=HADHA PE=1 SV=2 - [ECHA_HUMAN]	5.81E-03	1.98E-02	up	1.198289
P52907	F-actin-capping protein subunit alpha-1 OS=Homo sapiens GN=CAPZA1 PE=1 SV=3 - [CAZA1_HUMAN]	9.21E-04	6.09E-03	down	-1.482052
O75347	Tubulin-specific chaperone A OS=Homo sapiens GN=TBCA PE=1 SV=3 - [TBCA_HUMAN]	6.10E-03	2.03E-02	down	-1.1259292
O00264	Membrane-associated progesterone receptor component 1 OS=Homo sapiens GN=PGRMC1 PE=1 SV=3 - [PGRC1_HUMAN]	5.31E-03	1.86E-02	up	1.391766
P35221	Catenin alpha-1 OS=Homo sapiens GN=CTNNA1 PE=1 SV=1 - [CTNA1_HUMAN]	2.30E-03	1.07E-02	up	1.3006402

P46777	60S ribosomal protein L5 OS=Homo sapiens GN=RPL5 PE=1 SV=3 - [RL5_HUMAN]	1.57E-02	3.87E-02	down	-1.133346
P61026	Ras-related protein Rab-10 OS=Homo sapiens GN=RAB10 PE=1 SV=1 - [RAB10_HUMAN]	1.19E-02	3.21E-02	up	1.2246257
P04080	Cystatin-B OS=Homo sapiens GN=CSTB PE=1 SV=2 - [CYTB_HUMAN]	1.33E-02	3.46E-02	up	1.2581038
P61586	Transforming protein RhoA OS=Homo sapiens GN=RHOA PE=1 SV=1 - [RHOA_HUMAN]	9.88E-03	2.79E-02	up	1.2297707
P33993	DNA replication licensing factor MCM7 OS=Homo sapiens GN=MCM7 PE=1 SV=4 - [MCM7_HUMAN]	9.86E-04	6.21E-03	down	-1.3645427
P23526	Adenosylhomocysteinase OS=Homo sapiens GN=AHCY PE=1 SV=4 - [SAHH_HUMAN]	1.32E-03	7.37E-03	down	-1.2220912
Q16891	MICOS complex subunit MIC60 OS=Homo sapiens GN=IMMT PE=1 SV=1 - [MIC60_HUMAN]	5.65E-03	1.94E-02	up	1.2135626
P20339	Ras-related protein Rab-5A OS=Homo sapiens GN=RAB5A PE=1 SV=2 - [RAB5A_HUMAN]	1.16E-02	3.15E-02	up	1.2418282
P05198	Eukaryotic translation initiation factor 2 subunit 1 OS=Homo sapiens GN=EIF2S1 PE=1 SV=3 - [IF2A_HUMAN]	4.26E-03	1.61E-02	down	-1.2081513
P62851	40S ribosomal protein S25 OS=Homo sapiens GN=RPS25 PE=1 SV=1 - [RS25_HUMAN]	8.74E-03	2.58E-02	down	-1.1635156
Q15149	Plectin OS=Homo sapiens GN=PLEC PE=1 SV=3 - [PLEC_HUMAN]	4.65E-03	1.68E-02	up	1.239284
P33316	Deoxyuridine 5'-triphosphate nucleotidohydrolase, mitochondrial OS=Homo sapiens GN=DUT PE=1 SV=4 - [DUT_HUMAN]	1.03E-03	6.39E-03	down	-1.6526828
O00303	Eukaryotic translation initiation factor 3 subunit F OS=Homo sapiens GN=EIF3F PE=1 SV=1 - [EIF3F_HUMAN]	4.07E-03	1.56E-02	down	-1.1988128
Q92688	Acidic leucine-rich nuclear phosphoprotein 32 family member B OS=Homo sapiens GN=ANP32B PE=1 SV=1 - [AN32B_HUMAN]	1.34E-03	7.38E-03	up	1.3922219
P04181	Ornithine aminotransferase, mitochondrial OS=Homo sapiens GN=OAT PE=1 SV=1 - [OAT_HUMAN]	2.08E-02	4.78E-02	up	1.2362257
P00441	Superoxide dismutase [Cu-Zn] OS=Homo sapiens GN=SOD1 PE=1 SV=2 - [SODC_HUMAN]	1.91E-02	4.47E-02	up	1.169954
P35613	Basigin OS=Homo sapiens GN=BSG PE=1 SV=2 - [BASI_HUMAN]	2.83E-03	1.24E-02	down	-1.4603549
P30044	Peroxiredoxin-5, mitochondrial OS=Homo sapiens GN=PRDX5 PE=1 SV=4 - [PRDX5_HUMAN]	4.86E-03	1.73E-02	up	1.3072814
P19338	Nucleolin OS=Homo sapiens GN=NCL PE=1 SV=3 - [NUCL_HUMAN]	6.74E-04	5.11E-03	down	-1.3032299
Q9Y617	Phosphoserine aminotransferase OS=Homo sapiens GN=PSAT1 PE=1 SV=2 - [SERC_HUMAN]	5.72E-04	4.65E-03	up	1.1873496
Q04828	Aldo-keto reductase family 1 member C1 OS=Homo sapiens GN=AKR1C1 PE=1 SV=1 - [AK1C1_HUMAN]	1.10E-04	2.10E-03	up	2.9348218
O43390	Heterogeneous nuclear ribonucleoprotein R OS=Homo sapiens GN=HNRNPR PE=1 SV=1 - [HNRPR_HUMAN]	1.15E-04	2.14E-03	down	-1.4232169
Q9NR30	Nucleolar RNA helicase 2 OS=Homo sapiens GN=DDX21 PE=1 SV=5 - [DDX21_HUMAN]	1.93E-04	2.70E-03	down	-1.6687161
P20700	Lamin-B1 OS=Homo sapiens GN=LMNB1 PE=1 SV=2 - [LMNB1_HUMAN]	4.39E-04	3.99E-03	down	-1.4446604
P67809	Nuclease-sensitive element-binding protein 1 OS=Homo sapiens GN=YBX1 PE=1 SV=3 - [YBOX1_HUMAN]	1.67E-02	4.04E-02	down	-1.1535116
P22314	Ubiquitin-like modifier-activating enzyme 1 OS=Homo sapiens GN=UBA1 PE=1 SV=3 - [UBA1_HUMAN]	1.43E-03	7.73E-03	down	-1.1387231
P40429	60S ribosomal protein L13a OS=Homo sapiens GN=RPL13A PE=1 SV=2 - [RL13A_HUMAN]	7.47E-04	5.41E-03	down	-1.1757252
P11940	Polyadenylate-binding protein 1 OS=Homo sapiens GN=PABPC1 PE=1 SV=2 - [PABP1_HUMAN]	7.66E-04	5.50E-03	down	-1.1792278
P37837	Transaldolase OS=Homo sapiens GN=TALDO1 PE=1 SV=2 - [TALDO_HUMAN]	1.42E-04	2.43E-03	up	1.5685321
Q9NUQ9	Protein FAM49B OS=Homo sapiens GN=FAM49B PE=1 SV=1 - [FA49B_HUMAN]	2.48E-04	3.05E-03	up	1.3108495
Q99986	Serine/threonine-protein kinase VRK1 OS=Homo sapiens GN=VRK1 PE=1 SV=1 - [VRK1_HUMAN]	9.19E-04	6.09E-03	down	-1.5845141
Q9BUF5	Tubulin beta-6 chain OS=Homo sapiens GN=TUBB6 PE=1 SV=1 - [TBB6_HUMAN]	4.40E-03	1.64E-02	down	-1.327108
Q13185	Chromobox protein homolog 3 OS=Homo sapiens GN=CBX3 PE=1 SV=4 - [CBX3_HUMAN]	1.03E-02	2.88E-02	down	-1.2591394
Q9Y4L1	Hypoxia up-regulated protein 1 OS=Homo sapiens GN=HYOU1 PE=1 SV=1 - [HYOU1_HUMAN]	7.35E-04	5.38E-03	up	1.3731519

Q13242	Serine/arginine-rich splicing factor 9 OS=Homo sapiens GN=SRSF9 PE=1 SV=1 - [SRSF9_HUMAN]	9.52E-04	6.11E-03	down	-1.2412403
P42330	Aldo-keto reductase family 1 member C3 OS=Homo sapiens GN=AKR1C3 PE=1 SV=4 - [AK1C3_HUMAN]	1.41E-03	7.65E-03	up	2.4039125
P12268	Inosine-5'-monophosphate dehydrogenase 2 OS=Homo sapiens GN=IMPDH2 PE=1 SV=2 - [IMDH2_HUMAN]	4.93E-04	4.30E-03	down	-1.2885474
Q99798	Aconitate hydratase, mitochondrial OS=Homo sapiens GN=ACO2 PE=1 SV=2 - [ACON_HUMAN]	7.45E-04	5.41E-03	up	1.3294115
Q03135	Caveolin-1 OS=Homo sapiens GN=CAV1 PE=1 SV=4 - [CAV1_HUMAN]	1.70E-02	4.10E-02	down	-1.1861877
Q13310	Polyadenylate-binding protein 4 OS=Homo sapiens GN=PABPC4 PE=1 SV=1 - [PABP4_HUMAN]	8.23E-05	1.93E-03	down	-1.4894267
O76003	Glutaredoxin-3 OS=Homo sapiens GN=GLRX3 PE=1 SV=2 - [GLRX3_HUMAN]	8.96E-04	5.99E-03	down	-1.3019738
Q01081	Splicing factor U2AF 35 kDa subunit OS=Homo sapiens GN=U2AF1 PE=1 SV=3 - [U2AF1_HUMAN]	1.28E-02	3.36E-02	down	-1.1551194
P27824	Calnexin OS=Homo sapiens GN=CANX PE=1 SV=2 - [CALX_HUMAN]	8.45E-03	2.51E-02	up	1.2374053
O95433	Activator of 90 kDa heat shock protein ATPase homolog 1 OS=Homo sapiens GN=AHSA1 PE=1 SV=1 - [AHSA1_HUMAN]	1.64E-05	1.36E-03	down	-1.598835
P62333	26S protease regulatory subunit 10B OS=Homo sapiens GN=PSMC6 PE=1 SV=1 - [PRS10_HUMAN]	4.87E-03	1.73E-02	down	-1.24646
P30740	Leukocyte elastase inhibitor OS=Homo sapiens GN=SERPINB1 PE=1 SV=1 - [ILEU_HUMAN]	1.15E-02	3.13E-02	up	1.199689
P62913	60S ribosomal protein L11 OS=Homo sapiens GN=RPL11 PE=1 SV=2 - [RL11_HUMAN]	3.52E-03	1.41E-02	down	-1.2254583
P47756	F-actin-capping protein subunit beta OS=Homo sapiens GN=CAPZB PE=1 SV=4 - [CAPZB_HUMAN]	8.98E-05	1.93E-03	down	-1.361371
Q9P258	Protein RCC2 OS=Homo sapiens GN=RCC2 PE=1 SV=2 - [RCC2_HUMAN]	1.63E-04	2.44E-03	down	-1.5995828
Q92598	Heat shock protein 105 kDa OS=Homo sapiens GN=HSPH1 PE=1 SV=1 - [HS105_HUMAN]	8.57E-04	5.84E-03	down	-1.1677066
P38117	Electron transfer flavoprotein subunit beta OS=Homo sapiens GN=ETFB PE=1 SV=3 - [ETFB_HUMAN]	6.18E-05	1.78E-03	up	1.4257207
P50914	60S ribosomal protein L14 OS=Homo sapiens GN=RPL14 PE=1 SV=4 - [RL14_HUMAN]	3.58E-03	1.43E-02	down	-1.2302359
P09972	Fructose-bisphosphate aldolase C OS=Homo sapiens GN=ALDOC PE=1 SV=2 - [ALDOC_HUMAN]	9.05E-03	2.65E-02	up	1.2637615
P35579	Myosin-9 OS=Homo sapiens GN=MYH9 PE=1 SV=4 - [MYH9_HUMAN]	7.89E-05	1.93E-03	down	-1.3237699
P31153	S-adenosylmethionine synthase isoform type-2 OS=Homo sapiens GN=MAT2A PE=1 SV=1 - [METK2_HUMAN]	1.72E-02	4.13E-02	down	-1.2178786
P49915	GMP synthase [glutamine-hydrolyzing] OS=Homo sapiens GN=GMPS PE=1 SV=1 - [GUAA_HUMAN]	7.50E-04	5.41E-03	down	-1.1682175
Q9UKM9	RNA-binding protein Raly OS=Homo sapiens GN=RALY PE=1 SV=1 - [RALY_HUMAN]	5.28E-04	4.49E-03	down	-1.1826164
P15924	Desmoplakin OS=Homo sapiens GN=DSP PE=1 SV=3 - [DESP_HUMAN]	2.90E-04	3.28E-03	down	-1.4416785
Q9Y265	RuvB-like 1 OS=Homo sapiens GN=RUVBL1 PE=1 SV=1 - [RUVB1_HUMAN]	1.68E-02	4.07E-02	up	1.109814
P98179	RNA-binding protein 3 OS=Homo sapiens GN=RBM3 PE=1 SV=1 - [RBM3_HUMAN]	8.32E-04	5.74E-03	down	-2.3412771
P62879	Guanine nucleotide-binding protein G(I)/G(S)/G(T) subunit beta-2 OS=Homo sapiens GN=GNB2 PE=1 SV=3 - [GBB2_HUMAN]	3.31E-03	1.37E-02	down	-1.1979517
Q53GQ0	Very-long-chain 3-oxoacyl-CoA reductase OS=Homo sapiens GN=HSD17B12 PE=1 SV=2 - [DHB12_HUMAN]	4.31E-03	1.62E-02	up	1.3972734
P09110	3-ketoacyl-CoA thiolase, peroxisomal OS=Homo sapiens GN=ACAA1 PE=1 SV=2 - [THIK_HUMAN]	2.72E-04	3.20E-03	up	1.2407163
Q9NTK5	Obg-like ATPase 1 OS=Homo sapiens GN=OLA1 PE=1 SV=2 - [OLA1_HUMAN]	6.14E-04	4.89E-03	down	-1.3208947
O00571	ATP-dependent RNA helicase DDX3X OS=Homo sapiens GN=DDX3X PE=1 SV=3 - [DDX3X_HUMAN]	1.66E-02	4.03E-02	down	-1.2072935
Q9NX40	OCIA domain-containing protein 1 OS=Homo sapiens GN=OCIAD1 PE=1 SV=1 - [OCAD1_HUMAN]	1.49E-03	7.96E-03	up	1.1379675
P30048	Thioredoxin-dependent peroxide reductase, mitochondrial OS=Homo sapiens GN=PRDX3 PE=1 SV=3 - [PRDX3_HUMAN]	3.05E-03	1.29E-02	down	-1.1892427
P14868	Aspartate--tRNA ligase, cytoplasmic OS=Homo sapiens GN=DARS PE=1 SV=2 - [SYDC_HUMAN]	6.66E-03	2.15E-02	down	-1.161467

P53396	ATP-citrate synthase OS=Homo sapiens GN=ACLY PE=1 SV=3 - [ACLY_HUMAN]	1.69E-04	2.44E-03	up	1.3355421
Q9HAV7	GrpE protein homolog 1, mitochondrial OS=Homo sapiens GN=GRPEL1 PE=1 SV=2 - [GRPE1_HUMAN]	1.45E-02	3.68E-02	up	1.3305161
P13693	Translationally-controlled tumor protein OS=Homo sapiens GN=TPT1 PE=1 SV=1 - [TCTP_HUMAN]	2.90E-04	3.28E-03	down	-1.2956079
P06748	Nucleophosmin OS=Homo sapiens GN=NPM1 PE=1 SV=2 - [NPM_HUMAN]	1.44E-03	7.75E-03	down	-1.2478081
Q86VP6	Cullin-associated NEDD8-dissociated protein 1 OS=Homo sapiens GN=CAND1 PE=1 SV=2 - [CAND1_HUMAN]	1.54E-03	8.10E-03	up	1.1377778
P55084	Trifunctional enzyme subunit beta, mitochondrial OS=Homo sapiens GN=HADHB PE=1 SV=3 - [ECHB_HUMAN]	3.42E-04	3.55E-03	up	1.2763329
P07339	Cathepsin D OS=Homo sapiens GN=CTSD PE=1 SV=1 - [CATD_HUMAN]	6.07E-03	2.03E-02	up	1.1749836
P16615	Sarcoplasmic/endoplasmic reticulum calcium ATPase 2 OS=Homo sapiens GN=ATP2A2 PE=1 SV=1 - [AT2A2_HUMAN]	2.10E-03	9.98E-03	up	1.4429438
P07384	Calpain-1 catalytic subunit OS=Homo sapiens GN=CAPN1 PE=1 SV=1 - [CAN1_HUMAN]	7.07E-05	1.86E-03	up	1.4105902
Q12931	Heat shock protein 75 kDa, mitochondrial OS=Homo sapiens GN=TRAP1 PE=1 SV=3 - [TRAP1_HUMAN]	7.43E-03	2.27E-02	up	1.1836349
O15372	Eukaryotic translation initiation factor 3 subunit H OS=Homo sapiens GN=EIF3H PE=1 SV=1 - [EIF3H_HUMAN]	1.59E-03	8.28E-03	down	-1.2745551
Q9Y547	Intraflagellar transport protein 25 homolog OS=Homo sapiens GN=HSPB11 PE=1 SV=1 - [IFT25_HUMAN]	5.95E-05	1.78E-03	down	-1.5542209
P48163	NADP-dependent malic enzyme OS=Homo sapiens GN=ME1 PE=1 SV=1 - [MAOX_HUMAN]	3.25E-04	3.46E-03	up	1.3942713
P46926	Glucosamine-6-phosphate isomerase 1 OS=Homo sapiens GN=GNPDA1 PE=1 SV=1 - [GNPI1_HUMAN]	2.08E-05	1.36E-03	up	1.8899547
O76021	Ribosomal L1 domain-containing protein 1 OS=Homo sapiens GN=RSL1D1 PE=1 SV=3 - [RL1D1_HUMAN]	7.43E-03	2.27E-02	down	-1.1990682
P54577	Tyrosine--tRNA ligase, cytoplasmic OS=Homo sapiens GN=YARS PE=1 SV=4 - [SYYC_HUMAN]	6.78E-03	2.17E-02	up	1.2195108
P06493	Cyclin-dependent kinase 1 OS=Homo sapiens GN=CDK1 PE=1 SV=3 - [CDK1_HUMAN]	3.08E-04	3.39E-03	down	-1.476753
P49591	Serine--tRNA ligase, cytoplasmic OS=Homo sapiens GN=SARS PE=1 SV=3 - [SYSC_HUMAN]	1.01E-04	2.01E-03	down	-1.4985554
O75608	Acyl-protein thioesterase 1 OS=Homo sapiens GN=LYPLA1 PE=1 SV=1 - [LYPA1_HUMAN]	6.94E-04	5.21E-03	up	1.6011112
Q9Y696	Chloride intracellular channel protein 4 OS=Homo sapiens GN=CLIC4 PE=1 SV=4 - [CLIC4_HUMAN]	1.52E-04	2.44E-03	down	-1.4006624
O75083	WD repeat-containing protein 1 OS=Homo sapiens GN=WDR1 PE=1 SV=4 - [WDR1_HUMAN]	2.81E-03	1.24E-02	up	1.1481489
Q13151	Heterogeneous nuclear ribonucleoprotein A0 OS=Homo sapiens GN=HNRNPA0 PE=1 SV=1 - [ROA0_HUMAN]	9.43E-04	6.11E-03	down	-1.2118063
P27144	Adenylate kinase 4, mitochondrial OS=Homo sapiens GN=AK4 PE=1 SV=1 - [KAD4_HUMAN]	3.46E-03	1.41E-02	down	-1.3533028
P23381	Tryptophan--tRNA ligase, cytoplasmic OS=Homo sapiens GN=WARS PE=1 SV=2 - [SYWC_HUMAN]	4.46E-03	1.65E-02	up	1.2812287
Q96K17	Transcription factor BTF3 homolog 4 OS=Homo sapiens GN=BTF3L4 PE=1 SV=1 - [BT3L4_HUMAN]	5.85E-03	1.98E-02	down	-1.4531513
P32119	Peroxiredoxin-2 OS=Homo sapiens GN=PRDX2 PE=1 SV=5 - [PRDX2_HUMAN]	9.14E-03	2.65E-02	up	1.1597544
P52565	Rho GDP-dissociation inhibitor 1 OS=Homo sapiens GN=ARHGDIA PE=1 SV=3 - [GDIR1_HUMAN]	4.39E-03	1.64E-02	up	1.1318933
Q15233	Non-POU domain-containing octamer-binding protein OS=Homo sapiens GN=NONO PE=1 SV=4 - [NONO_HUMAN]	4.47E-04	4.02E-03	down	-1.1952565
Q96AG4	Leucine-rich repeat-containing protein 59 OS=Homo sapiens GN=LRRC59 PE=1 SV=1 - [LRC59_HUMAN]	8.19E-03	2.46E-02	up	1.1533757
Q9UBX3	Mitochondrial dicarboxylate carrier OS=Homo sapiens GN=SLC25A10 PE=1 SV=2 - [DIC_HUMAN]	1.11E-03	6.70E-03	up	1.5409805
P13796	Plastin-2 OS=Homo sapiens GN=LCP1 PE=1 SV=6 - [PLSL_HUMAN]	1.93E-05	1.36E-03	up	4.9645185
O15511	Actin-related protein 2/3 complex subunit 5 OS=Homo sapiens GN=ARPC5 PE=1 SV=3 - [ARPC5_HUMAN]	2.00E-02	4.64E-02	up	1.2004961
O75431	Metaxin-2 OS=Homo sapiens GN=MTX2 PE=1 SV=1 - [MTX2_HUMAN]	4.09E-03	1.56E-02	up	1.2855476
Q01813	ATP-dependent 6-phosphofructokinase, platelet type OS=Homo sapiens GN=PFKP PE=1 SV=2 - [PFKAP_HUMAN]	8.80E-03	2.59E-02	up	1.1276958

Q9Y2X3	Nucleolar protein 58 OS=Homo sapiens GN=NOP58 PE=1 SV=1 - [NOP58_HUMAN]	8.53E-04	5.84E-03	down	-1.2856553
P54886	Delta-1-pyrroline-5-carboxylate synthase OS=Homo sapiens GN=ALDH18A1 PE=1 SV=2 - [P5CS_HUMAN]	3.96E-04	3.73E-03	down	-1.4123482
P51665	26S proteasome non-ATPase regulatory subunit 7 OS=Homo sapiens GN=PSMD7 PE=1 SV=2 - [PSMD7_HUMAN]	1.72E-02	4.13E-02	down	-1.1022326
P55060	Exportin-2 OS=Homo sapiens GN=CSE1L PE=1 SV=3 - [XPO2_HUMAN]	1.90E-03	9.15E-03	down	-1.1749027
O75396	Vesicle-trafficking protein SEC22b OS=Homo sapiens GN=SEC22B PE=1 SV=4 - [SC22B_HUMAN]	3.44E-05	1.72E-03	down	-2.1398134
P16402	Histone H1.3 OS=Homo sapiens GN=HIST1H1D PE=1 SV=2 - [H13_HUMAN]	4.48E-03	1.65E-02	down	-1.5689797
Q14566	DNA replication licensing factor MCM6 OS=Homo sapiens GN=MCM6 PE=1 SV=1 - [MCM6_HUMAN]	3.42E-04	3.55E-03	down	-1.5707914
Q14103	Heterogeneous nuclear ribonucleoprotein D0 OS=Homo sapiens GN=HNRNPD PE=1 SV=1 - [HNRPD_HUMAN]	1.41E-02	3.60E-02	up	1.1316749
P46940	Ras GTPase-activating-like protein IQGAP1 OS=Homo sapiens GN=IQGAP1 PE=1 SV=1 - [IQGA1_HUMAN]	7.40E-03	2.27E-02	down	-1.1817677
P21333	Filamin-A OS=Homo sapiens GN=FLNA PE=1 SV=4 - [FLNA_HUMAN]	3.00E-03	1.28E-02	down	-1.3866814
P62829	60S ribosomal protein L23 OS=Homo sapiens GN=RPL23 PE=1 SV=1 - [RL23_HUMAN]	9.45E-03	2.71E-02	down	-1.1723456
O00151	PDZ and LIM domain protein 1 OS=Homo sapiens GN=PDLIM1 PE=1 SV=4 - [PDLI1_HUMAN]	2.28E-05	1.38E-03	down	-1.7290163
P36957	Dihydrolipoyllysine-residue succinyltransferase component of 2-oxoglutarate dehydrogenase complex, mitochondrial OS=Homo sapiens GN=DLST PE=1 SV=4 - [ODO2_HUMAN]	1.10E-02	3.03E-02	down	-1.1369902
P48506	Glutamate--cysteine ligase catalytic subunit OS=Homo sapiens GN=GCLC PE=1 SV=2 - [GSH1_HUMAN]	3.72E-05	1.78E-03	up	1.7526379
O00429	Dynamin-1-like protein OS=Homo sapiens GN=DNM1L PE=1 SV=2 - [DNM1L_HUMAN]	1.58E-02	3.89E-02	up	1.1522093
P06737	Glycogen phosphorylase, liver form OS=Homo sapiens GN=PYGL PE=1 SV=4 - [PYGL_HUMAN]	4.23E-05	1.78E-03	down	-1.8788105
Q9Y3A5	Ribosome maturation protein SBDS OS=Homo sapiens GN=SBDS PE=1 SV=4 - [SBDS_HUMAN]	6.68E-04	5.11E-03	down	-1.3808237
P04179	Superoxide dismutase [Mn], mitochondrial OS=Homo sapiens GN=SOD2 PE=1 SV=2 - [SODM_HUMAN]	2.47E-03	1.13E-02	up	1.3963857
Q9BVK6	Transmembrane emp24 domain-containing protein 9 OS=Homo sapiens GN=TMED9 PE=1 SV=2 - [TMED9_HUMAN]	3.03E-03	1.29E-02	up	1.3050512
O00233	26S proteasome non-ATPase regulatory subunit 9 OS=Homo sapiens GN=PSMD9 PE=1 SV=3 - [PSMD9_HUMAN]	5.17E-04	4.41E-03	down	-1.390694
Q08J23	tRNA (cytosine(34)-C(5))-methyltransferase OS=Homo sapiens GN=NSUN2 PE=1 SV=2 - [NSUN2_HUMAN]	1.19E-02	3.22E-02	down	-1.2152523
P35998	26S protease regulatory subunit 7 OS=Homo sapiens GN=PSMC2 PE=1 SV=3 - [PRS7_HUMAN]	1.35E-03	7.38E-03	down	-1.1426486
P27797	Calreticulin OS=Homo sapiens GN=CALR PE=1 SV=1 - [CALR_HUMAN]	4.06E-04	3.77E-03	up	1.5843748
O43795	Unconventional myosin-Ib OS=Homo sapiens GN=MYO1B PE=1 SV=3 - [MYO1B_HUMAN]	1.52E-04	2.44E-03	down	-1.2396137
P24539	ATP synthase F(0) complex subunit B1, mitochondrial OS=Homo sapiens GN=ATP5F1 PE=1 SV=2 - [AT5F1_HUMAN]	3.09E-04	3.39E-03	down	-1.2738857
Q14204	Cytoplasmic dynein 1 heavy chain 1 OS=Homo sapiens GN=DYNC1H1 PE=1 SV=5 - [DYHC1_HUMAN]	6.09E-05	1.78E-03	up	1.6032346
Q13162	Peroxiredoxin-4 OS=Homo sapiens GN=PRDX4 PE=1 SV=1 - [PRDX4_HUMAN]	4.84E-05	1.78E-03	up	1.8027271
Q13200	26S proteasome non-ATPase regulatory subunit 2 OS=Homo sapiens GN=PSMD2 PE=1 SV=3 - [PSMD2_HUMAN]	1.34E-02	3.47E-02	down	-1.2142394
Q13057	Bifunctional coenzyme A synthase OS=Homo sapiens GN=COASY PE=1 SV=4 - [COASY_HUMAN]	2.49E-03	1.13E-02	up	1.1592919
P09758	Tumor-associated calcium signal transducer 2 OS=Homo sapiens GN=TACSTD2 PE=1 SV=3 - [TACD2_HUMAN]	9.11E-03	2.65E-02	down	-1.2121285
Q14152	Eukaryotic translation initiation factor 3 subunit A OS=Homo sapiens GN=EIF3A PE=1 SV=1 - [EIF3A_HUMAN]	3.84E-03	1.49E-02	down	-1.1910186
P41091	Eukaryotic translation initiation factor 2 subunit 3 OS=Homo sapiens GN=EIF2S3 PE=1 SV=3 - [IF2G_HUMAN]	2.29E-04	2.87E-03	down	-1.2338092
P16949	Stathmin OS=Homo sapiens GN=STMN1 PE=1 SV=3 - [STMN1_HUMAN]	5.90E-03	1.98E-02	down	-1.3613175
P22676	Calretinin OS=Homo sapiens GN=CALB2 PE=2 SV=2 - [CALB2_HUMAN]	3.13E-06	8.76E-04	up	3.1833932

Q9BY44	Eukaryotic translation initiation factor 2A OS=Homo sapiens GN=EIF2A PE=1 SV=3 - [EIF2A_HUMAN]	1.25E-02	3.30E-02	down	-1.2347101
P30838	Aldehyde dehydrogenase, dimeric NADP-preferring OS=Homo sapiens GN=ALDH3A1 PE=1 SV=3 - [AL3A1_HUMAN]	9.71E-06	1.36E-03	up	1.8669542
Q15785	Mitochondrial import receptor subunit TOM34 OS=Homo sapiens GN=TOMM34 PE=1 SV=2 - [TOM34_HUMAN]	1.63E-03	8.34E-03	down	-1.2463759
Q06210	Glutamine--fructose-6-phosphate aminotransferase [isomerizing] 1 OS=Homo sapiens GN=GFPT1 PE=1 SV=3 - [GFPT1_HUMAN]	1.21E-03	7.05E-03	up	1.3379711
Q13136	Liprin-alpha-1 OS=Homo sapiens GN=PPFIA1 PE=1 SV=1 - [LIPA1_HUMAN]	5.42E-04	4.57E-03	down	-1.3497835
P21964	Catechol O-methyltransferase OS=Homo sapiens GN=COMT PE=1 SV=2 - [COMT_HUMAN]	1.37E-02	3.52E-02	up	1.3126211
P31689	DnaJ homolog subfamily A member 1 OS=Homo sapiens GN=DNAJA1 PE=1 SV=2 - [DNJA1_HUMAN]	1.18E-02	3.19E-02	down	-1.3605753
Q9UHX1	Poly(U)-binding-splicing factor PUF60 OS=Homo sapiens GN=PUF60 PE=1 SV=1 - [PUF60_HUMAN]	8.15E-03	2.45E-02	down	-1.1477194
Q9BT78	COP9 signalosome complex subunit 4 OS=Homo sapiens GN=COPS4 PE=1 SV=1 - [CSN4_HUMAN]	3.47E-03	1.41E-02	up	1.1351424
Q9Y3I0	tRNA-splicing ligase RtcB homolog OS=Homo sapiens GN=RTCB PE=1 SV=1 - [RTCB_HUMAN]	2.17E-02	4.93E-02	up	1.1991403
P27105	Erythrocyte band 7 integral membrane protein OS=Homo sapiens GN=STOM PE=1 SV=3 - [STOM_HUMAN]	1.55E-03	8.14E-03	down	-1.3997545
P51572	B-cell receptor-associated protein 31 OS=Homo sapiens GN=BCAP31 PE=1 SV=3 - [BAP31_HUMAN]	1.83E-02	4.34E-02	up	1.2099215
P61221	ATP-binding cassette sub-family E member 1 OS=Homo sapiens GN=ABCE1 PE=1 SV=1 - [ABCE1_HUMAN]	2.61E-03	1.17E-02	down	-1.1531037
P63172	Dynein light chain Tctex-type 1 OS=Homo sapiens GN=DYNLT1 PE=1 SV=1 - [DYLT1_HUMAN]	1.58E-02	3.88E-02	up	1.6481178
P55786	Puromycin-sensitive aminopeptidase OS=Homo sapiens GN=NPEPPS PE=1 SV=2 - [PSA_HUMAN]	5.44E-03	1.88E-02	up	1.1913173
Q16851	UTP--glucose-1-phosphate uridylyltransferase OS=Homo sapiens GN=UGP2 PE=1 SV=5 - [UGPA_HUMAN]	4.64E-05	1.78E-03	up	1.4884177
O95336	6-phosphogluconolactonase OS=Homo sapiens GN=PGLS PE=1 SV=2 - [6PGL_HUMAN]	1.04E-03	6.43E-03	up	1.148455
Q15005	Signal peptidase complex subunit 2 OS=Homo sapiens GN=SPCS2 PE=1 SV=3 - [SPCS2_HUMAN]	4.79E-03	1.70E-02	up	1.3009044
Q9Y624	Junctional adhesion molecule A OS=Homo sapiens GN=F11R PE=1 SV=1 - [JAM1_HUMAN]	3.31E-04	3.50E-03	up	1.5451335
Q96AE4	Far upstream element-binding protein 1 OS=Homo sapiens GN=FUBP1 PE=1 SV=3 - [FUBP1_HUMAN]	3.97E-03	1.53E-02	down	-1.3270313
P56192	Methionine--tRNA ligase, cytoplasmic OS=Homo sapiens GN=MARS PE=1 SV=2 - [SYMC_HUMAN]	3.71E-03	1.46E-02	down	-1.137392
P32969	60S ribosomal protein L9 OS=Homo sapiens GN=RPL9 PE=1 SV=1 - [RL9_HUMAN]	5.86E-03	1.98E-02	down	-1.1909722
Q9HA64	Ketosamine-3-kinase OS=Homo sapiens GN=FN3KRP PE=1 SV=2 - [KT3K_HUMAN]	7.98E-03	2.41E-02	up	1.3307159
P49736	DNA replication licensing factor MCM2 OS=Homo sapiens GN=MCM2 PE=1 SV=4 - [MCM2_HUMAN]	4.51E-04	4.03E-03	down	-1.5317193
Q9NVI7	ATPase family AAA domain-containing protein 3A OS=Homo sapiens GN=ATAD3A PE=1 SV=2 - [ATD3A_HUMAN]	2.23E-03	1.05E-02	down	-1.5860535
P28074	Proteasome subunit beta type-5 OS=Homo sapiens GN=PSMB5 PE=1 SV=3 - [PSB5_HUMAN]	9.95E-04	6.23E-03	down	-1.5448285
P21281	V-type proton ATPase subunit B, brain isoform OS=Homo sapiens GN=ATP6V1B2 PE=1 SV=3 - [VATB2_HUMAN]	7.79E-04	5.55E-03	up	1.5195359
O60547	GDP-mannose 4,6 dehydratase OS=Homo sapiens GN=GMDS PE=1 SV=1 - [GMDS_HUMAN]	1.20E-03	7.02E-03	up	1.40803
P60228	Eukaryotic translation initiation factor 3 subunit E OS=Homo sapiens GN=EIF3E PE=1 SV=1 - [EIF3E_HUMAN]	7.08E-03	2.22E-02	down	-1.2316507
Q02218	2-oxoglutarate dehydrogenase, mitochondrial OS=Homo sapiens GN=OGDH PE=1 SV=3 - [ODO1_HUMAN]	3.68E-03	1.46E-02	down	-1.2107928
P38606	V-type proton ATPase catalytic subunit A OS=Homo sapiens GN=ATP6V1A PE=1 SV=2 - [VATA_HUMAN]	7.61E-03	2.32E-02	up	1.2802314
O95881	Thioredoxin domain-containing protein 12 OS=Homo sapiens GN=TXNDC12 PE=1 SV=1 - [TXD12_HUMAN]	3.54E-04	3.58E-03	down	-1.6267446
Q5VWZ2	Lysophospholipase-like protein 1 OS=Homo sapiens GN=LYPLAL1 PE=1 SV=3 - [LYPL1_HUMAN]	9.66E-04	6.16E-03	up	1.2498306
P14324	Farnesyl pyrophosphate synthase OS=Homo sapiens GN=FDPS PE=1 SV=4 - [FPPS_HUMAN]	1.09E-02	3.02E-02	up	1.227648

P18206	Vinculin OS=Homo sapiens GN=VCL PE=1 SV=4 - [VINC_HUMAN]	1.35E-02	3.49E-02	down	-1.2371044
P15374	Ubiquitin carboxyl-terminal hydrolase isozyme L3 OS=Homo sapiens GN=UCHL3 PE=1 SV=1 - [UCHL3_HUMAN]	2.02E-02	4.67E-02	up	1.2342337
Q15382	GTP-binding protein Rheb OS=Homo sapiens GN=RHEB PE=1 SV=1 - [RHEB_HUMAN]	4.97E-03	1.76E-02	up	1.4957985
P15153	Ras-related C3 botulinum toxin substrate 2 OS=Homo sapiens GN=RAC2 PE=1 SV=1 - [RAC2_HUMAN]	3.71E-04	3.61E-03	up	2.020465
Q9BR76	Coronin-1B OS=Homo sapiens GN=CORO1B PE=1 SV=1 - [COR1B_HUMAN]	5.38E-03	1.88E-02	up	1.1234653
P55263	Adenosine kinase OS=Homo sapiens GN=ADK PE=1 SV=2 - [ADK_HUMAN]	6.36E-03	2.08E-02	down	-1.3790295
P12081	Histidine--tRNA ligase, cytoplasmic OS=Homo sapiens GN=HARS PE=1 SV=2 - [SYHC_HUMAN]	8.68E-04	5.87E-03	down	-1.2718859
P39748	Flap endonuclease 1 OS=Homo sapiens GN=FEN1 PE=1 SV=1 - [FEN1_HUMAN]	3.01E-04	3.36E-03	up	1.3293201
Q12907	Vesicular integral-membrane protein VIP36 OS=Homo sapiens GN=LMAN2 PE=1 SV=1 - [LMAN2_HUMAN]	3.07E-05	1.63E-03	up	1.4461673
Q7Z2W4	Zinc finger CCCH-type antiviral protein 1 OS=Homo sapiens GN=ZC3HAV1 PE=1 SV=3 - [ZCCHV_HUMAN]	1.50E-04	2.44E-03	down	-1.8808278
Q9Y3F4	Serine-threonine kinase receptor-associated protein OS=Homo sapiens GN=STRAP PE=1 SV=1 - [STRAP_HUMAN]	3.73E-04	3.61E-03	down	-1.5498998
Q8TC12	Retinol dehydrogenase 11 OS=Homo sapiens GN=RDH11 PE=1 SV=2 - [RDH11_HUMAN]	3.19E-03	1.34E-02	down	-1.3041362
P30043	Flavin reductase (NADPH) OS=Homo sapiens GN=BLVRB PE=1 SV=3 - [BLVRB_HUMAN]	6.34E-04	4.98E-03	up	1.7419794
Q03169	Tumor necrosis factor alpha-induced protein 2 OS=Homo sapiens GN=TNFAIP2 PE=2 SV=2 - [TNAP2_HUMAN]	4.31E-05	1.78E-03	up	2.116569
P30405	Peptidyl-prolyl cis-trans isomerase F, mitochondrial OS=Homo sapiens GN=PPIF PE=1 SV=1 - [PPIF_HUMAN]	4.55E-03	1.66E-02	down	-1.2422752
O95831	Apoptosis-inducing factor 1, mitochondrial OS=Homo sapiens GN=AIFM1 PE=1 SV=1 - [AIFM1_HUMAN]	5.82E-03	1.98E-02	up	1.2126722
P62942	Peptidyl-prolyl cis-trans isomerase FKBP1A OS=Homo sapiens GN=FKBP1A PE=1 SV=2 - [FKB1A_HUMAN]	1.28E-02	3.36E-02	up	1.3899764
Q9Y376	Calcium-binding protein 39 OS=Homo sapiens GN=CAB39 PE=1 SV=1 - [CAB39_HUMAN]	1.19E-03	6.95E-03	down	-1.4694296
P13995	Bifunctional methylenetetrahydrofolate dehydrogenase/cyclohydrolase, mitochondrial OS=Homo sapiens GN=MTHFD2 PE=1 SV=2 - [MTDC_HUMAN]	7.02E-03	2.22E-02	down	-1.2777969
P36871	Phosphoglucomutase-1 OS=Homo sapiens GN=PGM1 PE=1 SV=3 - [PGM1_HUMAN]	1.53E-04	2.44E-03	down	-1.757677
Q14258	E3 ubiquitin/ISG15 ligase TRIM25 OS=Homo sapiens GN=TRIM25 PE=1 SV=2 - [TRI25_HUMAN]	1.43E-04	2.43E-03	up	1.6760329
Q7Z4W1	L-xylulose reductase OS=Homo sapiens GN=DCXR PE=1 SV=2 - [DCXR_HUMAN]	2.76E-04	3.20E-03	up	1.5713593
Q9Y262	Eukaryotic translation initiation factor 3 subunit L OS=Homo sapiens GN=EIF3L PE=1 SV=1 - [EIF3L_HUMAN]	8.17E-03	2.45E-02	down	-1.1597643
Q96RS6	NudC domain-containing protein 1 OS=Homo sapiens GN=NUDCD1 PE=1 SV=2 - [NUDC1_HUMAN]	1.85E-03	9.01E-03	up	1.1416517
P48637	Glutathione synthetase OS=Homo sapiens GN=GSS PE=1 SV=1 - [GSHB_HUMAN]	1.20E-02	3.23E-02	down	-1.2317704
Q00059	Transcription factor A, mitochondrial OS=Homo sapiens GN=TFAM PE=1 SV=1 - [TFAM_HUMAN]	1.86E-02	4.38E-02	down	-1.1820513
Q03252	Lamin-B2 OS=Homo sapiens GN=LMNB2 PE=1 SV=4 - [LMNB2_HUMAN]	6.05E-03	2.03E-02	down	-1.1603619
O14933	Ubiquitin/ISG15-conjugating enzyme E2 L6 OS=Homo sapiens GN=UBE2L6 PE=1 SV=4 - [UB2L6_HUMAN]	3.24E-03	1.34E-02	up	1.6019629
P30519	Heme oxygenase 2 OS=Homo sapiens GN=HMOX2 PE=1 SV=2 - [HMOX2_HUMAN]	2.17E-02	4.93E-02	up	1.1141288
O95373	Importin-7 OS=Homo sapiens GN=IPO7 PE=1 SV=1 - [IPO7_HUMAN]	1.02E-02	2.87E-02	down	-1.1922915
Q96DI7	U5 small nuclear ribonucleoprotein 40 kDa protein OS=Homo sapiens GN=SNRNP40 PE=1 SV=1 - [SNR40_HUMAN]	1.81E-03	8.88E-03	down	-1.3128988
P26583	High mobility group protein B2 OS=Homo sapiens GN=HMGB2 PE=1 SV=2 - [HMGB2_HUMAN]	1.26E-02	3.34E-02	up	1.3599427
Q9Y5M8	Signal recognition particle receptor subunit beta OS=Homo sapiens GN=SRPRB PE=1 SV=3 - [SRPRB_HUMAN]	3.88E-04	3.69E-03	up	1.4659659
Q96HS1	Serine/threonine-protein phosphatase PGAM5, mitochondrial OS=Homo sapiens GN=PGAM5 PE=1 SV=2 - [PGAM5_HUMAN]	3.57E-04	3.59E-03	down	-1.2412837

Q5JTV8	Torsin-1A-interacting protein 1 OS=Homo sapiens GN=TOR1AIP1 PE=1 SV=2 - [TOIP1_HUMAN]	1.16E-02	3.15E-02	up	1.2489173
Q92820	Gamma-glutamyl hydrolase OS=Homo sapiens GN=GGH PE=1 SV=2 - [GGH_HUMAN]	7.11E-05	1.86E-03	up	3.1326673
P11172	Uridine 5'-monophosphate synthase OS=Homo sapiens GN=UMPS PE=1 SV=1 - [UMPS_HUMAN]	8.03E-04	5.65E-03	up	1.3767604
Q9NQR4	Omega-amidase NIT2 OS=Homo sapiens GN=NIT2 PE=1 SV=1 - [NIT2_HUMAN]	1.78E-03	8.86E-03	up	1.1305518
Q9UL25	Ras-related protein Rab-21 OS=Homo sapiens GN=RAB21 PE=1 SV=3 - [RAB21_HUMAN]	8.73E-03	2.58E-02	up	1.1660937
P35270	Sepiapterin reductase OS=Homo sapiens GN=SPR PE=1 SV=1 - [SPRE_HUMAN]	9.94E-03	2.80E-02	up	1.4115462
P15121	Aldose reductase OS=Homo sapiens GN=AKR1B1 PE=1 SV=3 - [ALDR_HUMAN]	1.68E-03	8.49E-03	up	1.3091357
Q13247	Serine/arginine-rich splicing factor 6 OS=Homo sapiens GN=SRSF6 PE=1 SV=2 - [SRSF6_HUMAN]	1.72E-02	4.13E-02	down	-1.1612135
O75533	Splicing factor 3B subunit 1 OS=Homo sapiens GN=SF3B1 PE=1 SV=3 - [SF3B1_HUMAN]	1.06E-04	2.07E-03	down	-1.3605387
Q9NSD9	Phenylalanine--tRNA ligase beta subunit OS=Homo sapiens GN=FARSB PE=1 SV=3 - [SYFB_HUMAN]	1.01E-05	1.36E-03	down	-1.247156
Q7Z7H5	Transmembrane emp24 domain-containing protein 4 OS=Homo sapiens GN=TMED4 PE=1 SV=1 - [TMED4_HUMAN]	3.20E-03	1.34E-02	down	-1.314663
O95299	NADH dehydrogenase [ubiquinone] 1 alpha subcomplex subunit 10, mitochondrial OS=Homo sapiens GN=NDUFA10 PE=1 SV=1 - [NDUAA_HUMAN]	1.04E-02	2.90E-02	up	1.2588991
Q92599	Septin-8 OS=Homo sapiens GN=SEPT8 PE=1 SV=4 - [SEPT8_HUMAN]	2.20E-02	4.97E-02	up	1.1200876
Q96IU4	Protein ABHD14B OS=Homo sapiens GN=ABHD14B PE=1 SV=1 - [ABHEB_HUMAN]	2.18E-05	1.36E-03	up	1.8066789
Q13148	TAR DNA-binding protein 43 OS=Homo sapiens GN=TARDBP PE=1 SV=1 - [TADBP_HUMAN]	1.82E-02	4.32E-02	down	-1.3657229
P26440	Isovaleryl-CoA dehydrogenase, mitochondrial OS=Homo sapiens GN=IVD PE=1 SV=1 - [IVD_HUMAN]	1.61E-04	2.44E-03	up	1.5149204
P20073	Annexin A7 OS=Homo sapiens GN=ANXA7 PE=1 SV=3 - [ANXA7_HUMAN]	7.69E-03	2.34E-02	down	-1.1948313
Q99436	Proteasome subunit beta type-7 OS=Homo sapiens GN=PSMB7 PE=1 SV=1 - [PSB7_HUMAN]	1.65E-02	4.03E-02	down	-1.14481
Q99447	Ethanolamine-phosphate cytidylyltransferase OS=Homo sapiens GN=PCYT2 PE=1 SV=1 - [PCY2_HUMAN]	3.90E-04	3.69E-03	up	1.9419681
P24752	Acetyl-CoA acetyltransferase, mitochondrial OS=Homo sapiens GN=ACAT1 PE=1 SV=1 - [THIL_HUMAN]	2.01E-04	2.73E-03	up	1.4854594
P36542	ATP synthase subunit gamma, mitochondrial OS=Homo sapiens GN=ATP5C1 PE=1 SV=1 - [ATPG_HUMAN]	7.54E-05	1.91E-03	down	-1.4592322
P62714	Serine/threonine-protein phosphatase 2A catalytic subunit beta isoform OS=Homo sapiens GN=PPP2CB PE=1 SV=1 - [PP2AB_HUMAN]	1.15E-03	6.83E-03	up	1.2826419
P33992	DNA replication licensing factor MCM5 OS=Homo sapiens GN=MCM5 PE=1 SV=5 - [MCM5_HUMAN]	1.66E-04	2.44E-03	down	-1.2432284
Q16531	DNA damage-binding protein 1 OS=Homo sapiens GN=DDB1 PE=1 SV=1 - [DDB1_HUMAN]	1.82E-04	2.60E-03	up	1.2106942
P19367	Hexokinase-1 OS=Homo sapiens GN=HK1 PE=1 SV=3 - [HXK1_HUMAN]	5.34E-03	1.87E-02	down	-1.2471868
P12429	Annexin A3 OS=Homo sapiens GN=ANXA3 PE=1 SV=3 - [ANXA3_HUMAN]	9.44E-03	2.71E-02	up	1.108245
Q96P70	Importin-9 OS=Homo sapiens GN=IPO9 PE=1 SV=3 - [IPO9_HUMAN]	1.68E-02	4.06E-02	up	1.1196529
P45954	Short/branched chain specific acyl-CoA dehydrogenase, mitochondrial OS=Homo sapiens GN=ACADSB PE=1 SV=1 - [ACDSB_HUMAN]	3.90E-05	1.78E-03	up	1.1744773
Q9Y5B9	FACT complex subunit SPT16 OS=Homo sapiens GN=SUPT16H PE=1 SV=1 - [SP16H_HUMAN]	1.46E-02	3.68E-02	down	-1.176356
Q86W42	THO complex subunit 6 homolog OS=Homo sapiens GN=THOC6 PE=1 SV=1 - [THOC6_HUMAN]	1.74E-02	4.15E-02	up	1.2216566
P35222	Catenin beta-1 OS=Homo sapiens GN=CTNNB1 PE=1 SV=1 - [CTNB1_HUMAN]	2.00E-04	2.73E-03	down	-1.1231141
P52292	Importin subunit alpha-1 OS=Homo sapiens GN=KPNA2 PE=1 SV=1 - [IMA1_HUMAN]	5.66E-04	4.64E-03	down	-1.3627762
Q9H2U2	Inorganic pyrophosphatase 2, mitochondrial OS=Homo sapiens GN=PPA2 PE=1 SV=2 - [IPYR2_HUMAN]	3.58E-03	1.43E-02	up	1.383827
P08754	Guanine nucleotide-binding protein G(k) subunit alpha OS=Homo sapiens GN=GNAI3 PE=1 SV=3 - [GNAI3_HUMAN]	5.07E-04	4.38E-03	down	-1.5921568

P13861	cAMP-dependent protein kinase type II-alpha regulatory subunit OS=Homo sapiens GN=PRKAR2A PE=1 SV=2 - [KAP2_HUMAN]	2.99E-03	1.28E-02	down	-1.5732346
P13928	Annexin A8 OS=Homo sapiens GN=ANXA8 PE=1 SV=3 - [ANXA8_HUMAN]	6.20E-04	4.92E-03	down	-1.3408294
Q8TEX9	Importin-4 OS=Homo sapiens GN=IPO4 PE=1 SV=2 - [IPO4_HUMAN]	2.20E-02	4.97E-02	down	-1.1967877
P07205	Phosphoglycerate kinase 2 OS=Homo sapiens GN=PGK2 PE=1 SV=3 - [PGK2_HUMAN]	1.29E-02	3.38E-02	up	1.2785542
P01116	GTPase KRas OS=Homo sapiens GN=KRAS PE=1 SV=1 - [RASK_HUMAN]	1.09E-02	3.02E-02	down	-1.2984385
Q16563	Synaptophysin-like protein 1 OS=Homo sapiens GN=SYPL1 PE=1 SV=1 - [SYPL1_HUMAN]	2.06E-03	9.82E-03	up	1.2860348
Q9NWV4	UPF0587 protein C1orf123 OS=Homo sapiens GN=C1orf123 PE=1 SV=1 - [CA123_HUMAN]	1.29E-03	7.28E-03	down	-1.3113347
O75340	Programmed cell death protein 6 OS=Homo sapiens GN=PDCD6 PE=1 SV=1 - [PDCD6_HUMAN]	4.91E-04	4.30E-03	down	-2.1625555
P34896	Serine hydroxymethyltransferase, cytosolic OS=Homo sapiens GN=SHMT1 PE=1 SV=1 - [GLYC_HUMAN]	1.24E-03	7.10E-03	down	-1.3914781
O75947	ATP synthase subunit d, mitochondrial OS=Homo sapiens GN=ATP5H PE=1 SV=3 - [ATP5H_HUMAN]	7.32E-04	5.38E-03	down	-1.2155179
Q7L2H7	Eukaryotic translation initiation factor 3 subunit M OS=Homo sapiens GN=EIF3M PE=1 SV=1 - [EIF3M_HUMAN]	8.78E-04	5.89E-03	down	-1.139839
Q14512	Fibroblast growth factor-binding protein 1 OS=Homo sapiens GN=FGFBP1 PE=1 SV=1 - [FGFP1_HUMAN]	2.12E-02	4.84E-02	up	1.4420913
Q86UP2	Kinectin OS=Homo sapiens GN=KTN1 PE=1 SV=1 - [KTN1_HUMAN]	1.60E-04	2.44E-03	down	-1.4343544
O96019	Actin-like protein 6A OS=Homo sapiens GN=ACTL6A PE=1 SV=1 - [ACL6A_HUMAN]	9.03E-04	6.01E-03	down	-1.2459966
O95861	3'(2'),5'-bisphosphate nucleotidase 1 OS=Homo sapiens GN=BPNT1 PE=1 SV=1 - [BPNT1_HUMAN]	3.25E-04	3.46E-03	up	1.7618325
Q92616	eIF-2-alpha kinase activator GCN1 OS=Homo sapiens GN=GCN1 PE=1 SV=6 - [GCN1_HUMAN]	4.19E-04	3.84E-03	down	-1.2707899
Q9Y678	Coatomer subunit gamma-1 OS=Homo sapiens GN=COPG1 PE=1 SV=1 - [COPG1_HUMAN]	9.68E-03	2.76E-02	up	1.1939502
Q08380	Galectin-3-binding protein OS=Homo sapiens GN=LGALS3BP PE=1 SV=1 - [LG3BP_HUMAN]	2.14E-04	2.81E-03	up	1.935903
Q14558	Phosphoribosyl pyrophosphate synthase-associated protein 1 OS=Homo sapiens GN=PRPSAP1 PE=1 SV=2 - [KPRA_HUMAN]	3.39E-04	3.55E-03	up	1.3550591
Q13126	S-methyl-5'-thioadenosine phosphorylase OS=Homo sapiens GN=MTAP PE=1 SV=2 - [MTAP_HUMAN]	5.82E-05	1.78E-03	up	1.3452346
P22059	Oxysterol-binding protein 1 OS=Homo sapiens GN=OSBP PE=1 SV=1 - [OSBP1_HUMAN]	1.81E-02	4.31E-02	up	1.2997214
P30566	Adenylosuccinate lyase OS=Homo sapiens GN=ADSL PE=1 SV=2 - [PUR8_HUMAN]	6.61E-03	2.14E-02	down	-1.1616874
Q15029	116 kDa U5 small nuclear ribonucleoprotein component OS=Homo sapiens GN=EFTUD2 PE=1 SV=1 - [U5S1_HUMAN]	1.52E-02	3.80E-02	down	-1.109069
Q9Y446	Plakophilin-3 OS=Homo sapiens GN=PKP3 PE=1 SV=1 - [PKP3_HUMAN]	2.60E-04	3.11E-03	down	-1.6296604
P33991	DNA replication licensing factor MCM4 OS=Homo sapiens GN=MCM4 PE=1 SV=5 - [MCM4_HUMAN]	1.58E-04	2.44E-03	down	-1.4498371
Q12797	Aspartyl/asparaginyl beta-hydroxylase OS=Homo sapiens GN=ASPH PE=1 SV=3 - [ASPH_HUMAN]	5.06E-05	1.78E-03	up	1.4420688
P12532	Creatine kinase U-type, mitochondrial OS=Homo sapiens GN=CKMT1A PE=1 SV=1 - [KCRU_HUMAN]	1.91E-06	8.34E-04	up	3.3859243
Q9H307	Pinin OS=Homo sapiens GN=PNN PE=1 SV=4 - [PININ_HUMAN]	8.10E-03	2.44E-02	down	-1.192626
P53007	Tricarboxylate transport protein, mitochondrial OS=Homo sapiens GN=SLC25A1 PE=1 SV=2 - [TXTP_HUMAN]	4.00E-04	3.74E-03	up	1.2346582
Q9Y490	Talin-1 OS=Homo sapiens GN=TLN1 PE=1 SV=3 - [TLN1_HUMAN]	3.53E-03	1.42E-02	down	-1.1710095
Q9Y295	Developmentally-regulated GTP-binding protein 1 OS=Homo sapiens GN=DRG1 PE=1 SV=1 - [DRG1_HUMAN]	1.05E-03	6.45E-03	down	-1.2131886
Q13283	Ras GTPase-activating protein-binding protein 1 OS=Homo sapiens GN=G3BP1 PE=1 SV=1 - [G3BP1_HUMAN]	1.06E-02	2.93E-02	down	-1.1862748
Q6P2Q9	Pre-mRNA-processing-splicing factor 8 OS=Homo sapiens GN=PRPF8 PE=1 SV=2 - [PRP8_HUMAN]	1.13E-03	6.82E-03	down	-1.2123522
Q00325	Phosphate carrier protein, mitochondrial OS=Homo sapiens GN=SLC25A3 PE=1 SV=2 - [MPCP_HUMAN]	1.14E-02	3.12E-02	up	1.1472305

Q15393	Splicing factor 3B subunit 3 OS=Homo sapiens GN=SF3B3 PE=1 SV=4 - [SF3B3_HUMAN]	1.72E-02	4.13E-02	down	-1.1477252
Q9Y320	Thioredoxin-related transmembrane protein 2 OS=Homo sapiens GN=TMX2 PE=1 SV=1 - [TMX2_HUMAN]	6.58E-04	5.10E-03	up	1.483777
P28062	Proteasome subunit beta type-8 OS=Homo sapiens GN=PSMB8 PE=1 SV=3 - [PSB8_HUMAN]	1.18E-04	2.15E-03	up	1.6555768
P09622	Dihydrolipoyl dehydrogenase, mitochondrial OS=Homo sapiens GN=DLD PE=1 SV=2 - [DLDH_HUMAN]	1.64E-05	1.36E-03	up	1.3070242
Q9NZM1	Myoferlin OS=Homo sapiens GN=MYOF PE=1 SV=1 - [MYOF_HUMAN]	6.07E-05	1.78E-03	down	-1.2412878
P49321	Nuclear autoantigenic sperm protein OS=Homo sapiens GN=NASP PE=1 SV=2 - [NASP_HUMAN]	3.48E-03	1.41E-02	down	-1.6916473
P48147	Prolyl endopeptidase OS=Homo sapiens GN=PREP PE=1 SV=2 - [PPCE_HUMAN]	6.69E-03	2.15E-02	up	1.1596283
P00966	Argininosuccinate synthase OS=Homo sapiens GN=ASS1 PE=1 SV=2 - [ASSY_HUMAN]	4.41E-05	1.78E-03	up	2.498901
O15067	Phosphoribosylformylglycinamidine synthase OS=Homo sapiens GN=PFAS PE=1 SV=4 - [PUR4_HUMAN]	3.37E-03	1.39E-02	down	-1.4083506
Q15257	Serine/threonine-protein phosphatase 2A activator OS=Homo sapiens GN=PTPA PE=1 SV=3 - [PTPA_HUMAN]	1.51E-02	3.78E-02	up	1.2398322
P42224	Signal transducer and activator of transcription 1-alpha/beta OS=Homo sapiens GN=STAT1 PE=1 SV=2 - [STAT1_HUMAN]	1.33E-02	3.46E-02	down	-1.1049707
P50416	Carnitine O-palmitoyltransferase 1, liver isoform OS=Homo sapiens GN=CPT1A PE=1 SV=2 - [CPT1A_HUMAN]	4.26E-03	1.61E-02	up	1.353562
P41252	Isoleucine--tRNA ligase, cytoplasmic OS=Homo sapiens GN=IARS PE=1 SV=2 - [SYIC_HUMAN]	2.52E-03	1.14E-02	up	1.4402844
Q9P035	Very-long-chain (3R)-3-hydroxyacyl-CoA dehydratase 3 OS=Homo sapiens GN=HACD3 PE=1 SV=2 - [HACD3_HUMAN]	3.75E-03	1.47E-02	up	1.4539803
Q9BX40	Protein LSM14 homolog B OS=Homo sapiens GN=LSM14B PE=1 SV=1 - [LS14B_HUMAN]	1.30E-02	3.41E-02	up	1.4748756
P50897	Palmitoyl-protein thioesterase 1 OS=Homo sapiens GN=PPT1 PE=1 SV=1 - [PPT1_HUMAN]	1.35E-02	3.49E-02	down	-1.321179
Q7Z2K6	Endoplasmic reticulum metallopeptidase 1 OS=Homo sapiens GN=ERMP1 PE=1 SV=2 - [ERMP1_HUMAN]	8.34E-03	2.50E-02	up	1.1831948
P18031	Tyrosine-protein phosphatase non-receptor type 1 OS=Homo sapiens GN=PTPN1 PE=1 SV=1 - [PTN1_HUMAN]	5.80E-03	1.98E-02	up	1.2737365
P47755	F-actin-capping protein subunit alpha-2 OS=Homo sapiens GN=CAPZA2 PE=1 SV=3 - [CAZA2_HUMAN]	1.50E-04	2.44E-03	up	1.701005
Q99439	Calponin-2 OS=Homo sapiens GN=CNN2 PE=1 SV=4 - [CNN2_HUMAN]	1.62E-04	2.44E-03	down	-2.8696566
O75964	ATP synthase subunit g, mitochondrial OS=Homo sapiens GN=ATP5L PE=1 SV=3 - [ATP5L_HUMAN]	1.22E-02	3.26E-02	down	-1.3232837
O43291	Kunitz-type protease inhibitor 2 OS=Homo sapiens GN=SPINT2 PE=1 SV=2 - [SPIT2_HUMAN]	4.22E-03	1.60E-02	up	1.1236874
O75475	PC4 and SFRS1-interacting protein OS=Homo sapiens GN=PSIP1 PE=1 SV=1 - [PSIP1_HUMAN]	7.64E-05	1.91E-03	down	-2.1883593
P30443	HLA class I histocompatibility antigen, A-1 alpha chain OS=Homo sapiens GN=HLA-A PE=1 SV=1 - [1A01_HUMAN]	3.73E-04	3.61E-03	up	1.4614594
Q9BUL8	Programmed cell death protein 10 OS=Homo sapiens GN=PDCD10 PE=1 SV=1 - [PDC10_HUMAN]	8.06E-03	2.44E-02	up	1.1784003
P09525	Annexin A4 OS=Homo sapiens GN=ANXA4 PE=1 SV=4 - [ANXA4_HUMAN]	2.15E-04	2.81E-03	up	1.8130257
Q56VL3	OCIA domain-containing protein 2 OS=Homo sapiens GN=OCIAD2 PE=1 SV=1 - [OCAD2_HUMAN]	1.53E-02	3.81E-02	up	1.549294
Q8N884	Cyclic GMP-AMP synthase OS=Homo sapiens GN=MB21D1 PE=1 SV=2 - [CGAS_HUMAN]	5.43E-04	4.57E-03	down	-1.2495068
P48507	Glutamate--cysteine ligase regulatory subunit OS=Homo sapiens GN=GCLM PE=1 SV=1 - [GSH0_HUMAN]	1.37E-02	3.53E-02	down	-1.1450833
O94826	Mitochondrial import receptor subunit TOM70 OS=Homo sapiens GN=TOMM70 PE=1 SV=1 - [TOM70_HUMAN]	7.40E-03	2.27E-02	up	1.1188831
Q8WWM7	Ataxin-2-like protein OS=Homo sapiens GN=ATXN2L PE=1 SV=2 - [ATX2L_HUMAN]	1.19E-02	3.21E-02	down	-1.1336836
Q96TA1	Niban-like protein 1 OS=Homo sapiens GN=FAM129B PE=1 SV=3 - [NIBL1_HUMAN]	2.18E-04	2.81E-03	up	2.2896135
Q9Y394	Dehydrogenase/reductase SDR family member 7 OS=Homo sapiens GN=DHRS7 PE=1 SV=1 - [DHRS7_HUMAN]	9.43E-04	6.11E-03	up	1.1681348
P53621	Coatomer subunit alpha OS=Homo sapiens GN=COPA PE=1 SV=2 - [COPA_HUMAN]	1.29E-02	3.39E-02	up	1.1597196

Q9UKZ1	CCR4-NOT transcription complex subunit 11 OS=Homo sapiens GN=CNOT11 PE=1 SV=1 - [CNO11_HUMAN]	2.13E-02	4.85E-02	up	1.2121085
Q15050	Ribosome biogenesis regulatory protein homolog OS=Homo sapiens GN=RRS1 PE=1 SV=2 - [RRS1_HUMAN]	4.57E-03	1.66E-02	up	1.3037481
P21283	V-type proton ATPase subunit C 1 OS=Homo sapiens GN=ATP6V1C1 PE=1 SV=4 - [VATC1_HUMAN]	1.71E-03	8.55E-03	up	1.450572
Q14498	RNA-binding protein 39 OS=Homo sapiens GN=RBM39 PE=1 SV=2 - [RBM39_HUMAN]	8.25E-04	5.71E-03	down	-1.2205482
P23921	Ribonucleoside-diphosphate reductase large subunit OS=Homo sapiens GN=RRM1 PE=1 SV=1 - [RIR1_HUMAN]	1.53E-02	3.81E-02	down	-1.4626693
Q16222	UDP-N-acetylhexosamine pyrophosphorylase OS=Homo sapiens GN=UAP1 PE=1 SV=3 - [UAP1_HUMAN]	2.91E-03	1.27E-02	down	-1.4034836
Q9BV19	Uncharacterized protein C1orf50 OS=Homo sapiens GN=C1orf50 PE=1 SV=2 - [CA050_HUMAN]	1.30E-03	7.31E-03	down	-1.3427149
Q13724	Mannosyl-oligosaccharide glucosidase OS=Homo sapiens GN=MOGS PE=1 SV=5 - [MOGS_HUMAN]	9.50E-04	6.11E-03	up	1.4345765
P14550	Alcohol dehydrogenase [NADP(+)] OS=Homo sapiens GN=AKR1A1 PE=1 SV=3 - [AK1A1_HUMAN]	2.21E-03	1.04E-02	down	-1.3957207
Q13547	Histone deacetylase 1 OS=Homo sapiens GN=HDAC1 PE=1 SV=1 - [HDAC1_HUMAN]	1.43E-03	7.73E-03	down	-1.342396
Q15645	Pachytene checkpoint protein 2 homolog OS=Homo sapiens GN=TRIP13 PE=1 SV=2 - [PCH2_HUMAN]	1.56E-04	2.44E-03	down	-1.3168436
O60716	Catenin delta-1 OS=Homo sapiens GN=CTNND1 PE=1 SV=1 - [CTND1_HUMAN]	1.10E-02	3.03E-02	up	1.1260769
Q86TX2	Acyl-coenzyme A thioesterase 1 OS=Homo sapiens GN=ACOT1 PE=1 SV=1 - [ACOT1_HUMAN]	7.14E-03	2.23E-02	up	1.2569525
Q3LXA3	Triokinase/FMN cyclase OS=Homo sapiens GN=TKFC PE=1 SV=2 - [TKFC_HUMAN]	8.49E-05	1.93E-03	up	1.2506938
P21291	Cysteine and glycine-rich protein 1 OS=Homo sapiens GN=CSRP1 PE=1 SV=3 - [CSRP1_HUMAN]	6.90E-04	5.21E-03	up	1.6530378
P51809	Vesicle-associated membrane protein 7 OS=Homo sapiens GN=VAMP7 PE=1 SV=3 - [VAMP7_HUMAN]	4.54E-03	1.66E-02	down	-1.2780358
P18463	HLA class I histocompatibility antigen, B-37 alpha chain OS=Homo sapiens GN=HLA-B PE=1 SV=1 - [1B37_HUMAN]	5.91E-05	1.78E-03	up	2.1537585
O00159	Unconventional myosin-Ic OS=Homo sapiens GN=MYO1C PE=1 SV=4 - [MYO1C_HUMAN]	5.05E-03	1.78E-02	down	-1.3109862
P46108	Adapter molecule crk OS=Homo sapiens GN=CRK PE=1 SV=2 - [CRK_HUMAN]	1.25E-02	3.32E-02	down	-1.2291358
P23919	Thymidylate kinase OS=Homo sapiens GN=DTYMK PE=1 SV=4 - [KTHY_HUMAN]	4.63E-05	1.78E-03	down	-1.6315895
Q93009	Ubiquitin carboxyl-terminal hydrolase 7 OS=Homo sapiens GN=USP7 PE=1 SV=2 - [UBP7_HUMAN]	2.47E-03	1.13E-02	up	1.3057977
P50995	Annexin A11 OS=Homo sapiens GN=ANXA11 PE=1 SV=1 - [ANX11_HUMAN]	1.10E-03	6.70E-03	up	1.2172781
Q96A33	Coiled-coil domain-containing protein 47 OS=Homo sapiens GN=CCDC47 PE=1 SV=1 - [CCD47_HUMAN]	1.17E-04	2.15E-03	up	1.4912884
Q9BS26	Endoplasmic reticulum resident protein 44 OS=Homo sapiens GN=ERP44 PE=1 SV=1 - [ERP44_HUMAN]	2.65E-03	1.19E-02	up	1.3178169
O00203	AP-3 complex subunit beta-1 OS=Homo sapiens GN=AP3B1 PE=1 SV=3 - [AP3B1_HUMAN]	6.88E-03	2.19E-02	up	1.2140434
Q14914	Prostaglandin reductase 1 OS=Homo sapiens GN=PTGR1 PE=1 SV=2 - [PTGR1_HUMAN]	9.36E-06	1.36E-03	down	-1.5355291
Q9BQ52	Zinc phosphodiesterase ELAC protein 2 OS=Homo sapiens GN=ELAC2 PE=1 SV=2 - [RNZ2_HUMAN]	5.20E-03	1.83E-02	down	-1.6913863
O75494	Serine/arginine-rich splicing factor 10 OS=Homo sapiens GN=SRSF10 PE=1 SV=1 - [SRS10_HUMAN]	5.83E-03	1.98E-02	down	-1.3410664
Q9UHB6	LIM domain and actin-binding protein 1 OS=Homo sapiens GN=LIMA1 PE=1 SV=1 - [LIMA1_HUMAN]	1.44E-02	3.66E-02	down	-1.5125822
P09874	Poly [ADP-ribose] polymerase 1 OS=Homo sapiens GN=PARP1 PE=1 SV=4 - [PARP1_HUMAN]	1.04E-02	2.90E-02	up	1.3567923
P06746	DNA polymerase beta OS=Homo sapiens GN=POLB PE=1 SV=3 - [DPOLB_HUMAN]	1.53E-03	8.06E-03	down	-1.1838535
P00533	Epidermal growth factor receptor OS=Homo sapiens GN=EGFR PE=1 SV=2 - [EGFR_HUMAN]	1.64E-04	2.44E-03	down	-2.0065012
P58107	Epiplakin OS=Homo sapiens GN=EPPK1 PE=1 SV=2 - [EPIPL_HUMAN]	8.65E-05	1.93E-03	down	-1.2566304
P55884	Eukaryotic translation initiation factor 3 subunit B OS=Homo sapiens GN=EIF3B PE=1 SV=3 - [EIF3B_HUMAN]	5.64E-04	4.64E-03	down	-1.3158051

P09417	Dihydropteridine reductase OS=Homo sapiens GN=QDPR PE=1 SV=2 - [DHPR_HUMAN]	1.74E-04	2.50E-03	up	1.6191547
Q8WUA7	TBC1 domain family member 22A OS=Homo sapiens GN=TBC1D22A PE=1 SV=2 - [TB22A_HUMAN]	1.53E-02	3.81E-02	up	1.2787445
P17812	CTP synthase 1 OS=Homo sapiens GN=CTPS1 PE=1 SV=2 - [PYRG1_HUMAN]	6.05E-04	4.84E-03	down	-1.5289824
Q8TBX8	Phosphatidylinositol 5-phosphate 4-kinase type-2 gamma OS=Homo sapiens GN=PIP4K2C PE=1 SV=3 - [PI42C_HUMAN]	1.13E-02	3.09E-02	up	1.1981641
P07954	Fumarate hydratase, mitochondrial OS=Homo sapiens GN=FH PE=1 SV=3 - [FUMH_HUMAN]	2.61E-03	1.17E-02	up	1.586866
Q9H788	SH2 domain-containing protein 4A OS=Homo sapiens GN=SH2D4A PE=1 SV=1 - [SH24A_HUMAN]	2.03E-02	4.67E-02	up	1.3383296
Q9P2E9	Ribosome-binding protein 1 OS=Homo sapiens GN=RRBP1 PE=1 SV=4 - [RRBP1_HUMAN]	9.20E-03	2.66E-02	up	1.3453128
Q9NPH2	Inositol-3-phosphate synthase 1 OS=Homo sapiens GN=ISYNA1 PE=1 SV=1 - [INO1_HUMAN]	3.67E-03	1.45E-02	down	-1.439747
Q00341	Vigilin OS=Homo sapiens GN=HDLBP PE=1 SV=2 - [VIGLN_HUMAN]	8.02E-05	1.93E-03	down	-1.256816
Q9NR45	Sialic acid synthase OS=Homo sapiens GN=NANS PE=1 SV=2 - [SIAS_HUMAN]	1.60E-03	8.30E-03	up	2.017355
P51116	Fragile X mental retardation syndrome-related protein 2 OS=Homo sapiens GN=FXR2 PE=1 SV=2 - [FXR2_HUMAN]	9.66E-03	2.76E-02	down	-1.4320009
P07686	Beta-hexosaminidase subunit beta OS=Homo sapiens GN=HEXB PE=1 SV=3 - [HEXB_HUMAN]	5.18E-06	1.13E-03	up	2.9749308
P61163	Alpha-centractin OS=Homo sapiens GN=ACTR1A PE=1 SV=1 - [ACTZ_HUMAN]	7.69E-03	2.34E-02	down	-1.3391565
Q00403	Transcription initiation factor IIB OS=Homo sapiens GN=GTF2B PE=1 SV=1 - [TF2B_HUMAN]	9.04E-05	1.93E-03	down	-1.6268498
Q6IBS0	Twinfilin-2 OS=Homo sapiens GN=TWF2 PE=1 SV=2 - [TWF2_HUMAN]	1.29E-03	7.28E-03	down	-1.3557509
O60437	Periplakin OS=Homo sapiens GN=PPL PE=1 SV=4 - [PEPL_HUMAN]	1.86E-03	9.03E-03	up	1.3550858
P45974	Ubiquitin carboxyl-terminal hydrolase 5 OS=Homo sapiens GN=USP5 PE=1 SV=2 - [UBP5_HUMAN]	6.92E-03	2.20E-02	up	1.3926362
O75746	Calcium-binding mitochondrial carrier protein Aralar1 OS=Homo sapiens GN=SLC25A12 PE=1 SV=2 - [CMC1_HUMAN]	5.58E-03	1.93E-02	up	1.1514953
Q9H488	GDP-fucose protein O-fucosyltransferase 1 OS=Homo sapiens GN=POFUT1 PE=1 SV=1 - [OFUT1_HUMAN]	9.77E-04	6.18E-03	down	-1.2536441
Q9GZL7	Ribosome biogenesis protein WDR12 OS=Homo sapiens GN=WDR12 PE=1 SV=2 - [WDR12_HUMAN]	8.79E-03	2.59E-02	down	-1.407055
Q96GA7	Serine dehydratase-like OS=Homo sapiens GN=SDSL PE=1 SV=1 - [SDSL_HUMAN]	2.61E-04	3.11E-03	up	1.6641517
Q8N1G4	Leucine-rich repeat-containing protein 47 OS=Homo sapiens GN=LRRC47 PE=1 SV=1 - [LRC47_HUMAN]	5.98E-03	2.01E-02	down	-1.2038138
Q9UBU8	Mortality factor 4-like protein 1 OS=Homo sapiens GN=MORF4L1 PE=1 SV=2 - [MO4L1_HUMAN]	1.44E-02	3.66E-02	down	-1.2230835
Q9Y5Z4	Heme-binding protein 2 OS=Homo sapiens GN=HEBP2 PE=1 SV=1 - [HEBP2_HUMAN]	3.13E-04	3.39E-03	up	1.6592985
Q9BQG0	Myb-binding protein 1A OS=Homo sapiens GN=MYBBP1A PE=1 SV=2 - [MBB1A_HUMAN]	1.48E-03	7.93E-03	down	-1.2400677
P63279	SUMO-conjugating enzyme UBC9 OS=Homo sapiens GN=UBE2I PE=1 SV=1 - [UBC9_HUMAN]	3.04E-04	3.37E-03	up	1.4283125
O00154	Cytosolic acyl coenzyme A thioester hydrolase OS=Homo sapiens GN=ACOT7 PE=1 SV=3 - [BACH_HUMAN]	3.49E-03	1.41E-02	down	-1.4204521
Q9Y2Z0	Protein SGT1 homolog OS=Homo sapiens GN=SUGT1 PE=1 SV=3 - [SGT1_HUMAN]	4.44E-03	1.65E-02	down	-1.4518747
Q969L2	Protein MAL2 OS=Homo sapiens GN=MAL2 PE=1 SV=1 - [MAL2_HUMAN]	3.08E-03	1.30E-02	up	1.3011281
Q14232	Translation initiation factor eIF-2B subunit alpha OS=Homo sapiens GN=EIF2B1 PE=1 SV=1 - [EI2BA_HUMAN]	4.68E-03	1.68E-02	down	-1.2916558
Q9Y6N5	Sulfide:quinone oxidoreductase, mitochondrial OS=Homo sapiens GN=SQRDL PE=1 SV=1 - [SQRD_HUMAN]	3.78E-03	1.48E-02	up	1.2973895
Q96T76	MMS19 nucleotide excision repair protein homolog OS=Homo sapiens GN=MMS19 PE=1 SV=2 - [MMS19_HUMAN]	5.72E-03	1.96E-02	down	-1.409594
P25205	DNA replication licensing factor MCM3 OS=Homo sapiens GN=MCM3 PE=1 SV=3 - [MCM3_HUMAN]	2.47E-03	1.13E-02	down	-1.3640145
P36543	V-type proton ATPase subunit E 1 OS=Homo sapiens GN=ATP6V1E1 PE=1 SV=1 - [VATE1_HUMAN]	1.72E-03	8.57E-03	up	1.5892648

Q9P0V9	Septin-10 OS=Homo sapiens GN=SEPT10 PE=1 SV=2 - [SEP10_HUMAN]	2.29E-04	2.87E-03	down	-3.1691968
P36776	Lon protease homolog, mitochondrial OS=Homo sapiens GN=LONP1 PE=1 SV=2 - [LONM_HUMAN]	3.47E-04	3.55E-03	up	1.681468
Q9H6F5	Coiled-coil domain-containing protein 86 OS=Homo sapiens GN=CCDC86 PE=1 SV=1 - [CCD86_HUMAN]	1.12E-04	2.11E-03	down	-1.461012
P52815	39S ribosomal protein L12, mitochondrial OS=Homo sapiens GN=MRPL12 PE=1 SV=2 - [RM12_HUMAN]	2.06E-02	4.73E-02	up	1.1835872
Q9BSH4	Translational activator of cytochrome c oxidase 1 OS=Homo sapiens GN=TACO1 PE=1 SV=1 - [TACO1_HUMAN]	4.45E-04	4.02E-03	up	1.3613174
Q9Y266	Nuclear migration protein nudC OS=Homo sapiens GN=NUDC PE=1 SV=1 - [NUDC_HUMAN]	3.32E-05	1.71E-03	down	-1.9088488
P10114	Ras-related protein Rap-2a OS=Homo sapiens GN=RAP2A PE=1 SV=1 - [RAP2A_HUMAN]	6.37E-04	4.98E-03	up	1.537162
Q9BZE4	Nucleolar GTP-binding protein 1 OS=Homo sapiens GN=GTPBP4 PE=1 SV=3 - [NOG1_HUMAN]	1.66E-03	8.45E-03	down	-1.2020156
P19623	Spermidine synthase OS=Homo sapiens GN=SRM PE=1 SV=1 - [SPEE_HUMAN]	3.50E-06	8.76E-04	down	-2.0793793
Q9Y478	5'-AMP-activated protein kinase subunit beta-1 OS=Homo sapiens GN=PRKAB1 PE=1 SV=4 - [AAKB1_HUMAN]	4.13E-03	1.57E-02	up	1.2668242
Q53H12	Acylglycerol kinase, mitochondrial OS=Homo sapiens GN=AGK PE=1 SV=2 - [AGK_HUMAN]	8.42E-04	5.78E-03	up	1.1963671
O94979	Protein transport protein Sec31A OS=Homo sapiens GN=SEC31A PE=1 SV=3 - [SC31A_HUMAN]	1.90E-02	4.45E-02	up	1.2902408
Q8NF37	Lysophosphatidylcholine acyltransferase 1 OS=Homo sapiens GN=LPCAT1 PE=1 SV=2 - [PCAT1_HUMAN]	4.63E-03	1.68E-02	down	-1.2672658
P00749	Urokinase-type plasminogen activator OS=Homo sapiens GN=PLAU PE=1 SV=2 - [UROK_HUMAN]	7.36E-03	2.27E-02	up	1.5188869
Q9BSD7	Cancer-related nucleoside-triphosphatase OS=Homo sapiens GN=NTPCR PE=1 SV=1 - [NTPCR_HUMAN]	2.74E-04	3.20E-03	up	2.0379148
Q96GX9	Methylthioribulose-1-phosphate dehydratase OS=Homo sapiens GN=APIP PE=1 SV=1 - [MTNB_HUMAN]	1.61E-03	8.31E-03	up	1.1866856
Q9NZ08	Endoplasmic reticulum aminopeptidase 1 OS=Homo sapiens GN=ERAP1 PE=1 SV=3 - [ERAP1_HUMAN]	1.10E-03	6.69E-03	up	1.6819026
O60841	Eukaryotic translation initiation factor 5B OS=Homo sapiens GN=EIF5B PE=1 SV=4 - [IF2P_HUMAN]	1.50E-02	3.77E-02	down	-1.174676
P50895	Basal cell adhesion molecule OS=Homo sapiens GN=BCAM PE=1 SV=2 - [BCAM_HUMAN]	1.95E-03	9.38E-03	down	-1.5468674
Q96RP9	Elongation factor G, mitochondrial OS=Homo sapiens GN=GFM1 PE=1 SV=2 - [EFGM_HUMAN]	6.30E-03	2.08E-02	up	1.2896384
P56537	Eukaryotic translation initiation factor 6 OS=Homo sapiens GN=EIF6 PE=1 SV=1 - [IF6_HUMAN]	1.31E-04	2.30E-03	down	-1.5281272
Q9Y570	Protein phosphatase methylesterase 1 OS=Homo sapiens GN=PPME1 PE=1 SV=3 - [PPME1_HUMAN]	6.32E-03	2.08E-02	up	1.1573726
Q9Y5X3	Sorting nexin-5 OS=Homo sapiens GN=SNX5 PE=1 SV=1 - [SNX5_HUMAN]	4.21E-03	1.60E-02	up	1.2299447
P51812	Ribosomal protein S6 kinase alpha-3 OS=Homo sapiens GN=RPS6KA3 PE=1 SV=1 - [KS6A3_HUMAN]	1.07E-02	2.96E-02	up	1.1284436
Q8NFJ5	Retinoic acid-induced protein 3 OS=Homo sapiens GN=GPRC5A PE=1 SV=2 - [RAI3_HUMAN]	6.05E-05	1.78E-03	up	2.446458
Q12788	Transducin beta-like protein 3 OS=Homo sapiens GN=TBL3 PE=1 SV=2 - [TBL3_HUMAN]	8.94E-03	2.62E-02	down	-1.3035139
Q14344	Guanine nucleotide-binding protein subunit alpha-13 OS=Homo sapiens GN=GNA13 PE=1 SV=2 - [GNA13_HUMAN]	1.24E-04	2.21E-03	up	1.4162366
Q92530	Proteasome inhibitor PI31 subunit OS=Homo sapiens GN=PSMF1 PE=1 SV=2 - [PSMF1_HUMAN]	2.28E-03	1.07E-02	down	-1.3225527
O43813	LanC-like protein 1 OS=Homo sapiens GN=LANCL1 PE=1 SV=1 - [LANC1_HUMAN]	2.18E-04	2.81E-03	up	2.9125082
P04040	Catalase OS=Homo sapiens GN=CAT PE=1 SV=3 - [CATA_HUMAN]	2.54E-05	1.44E-03	up	2.2587867
Q99541	Perilipin-2 OS=Homo sapiens GN=PLIN2 PE=1 SV=2 - [PLIN2_HUMAN]	2.80E-03	1.24E-02	up	1.4395255
Q16666	Gamma-interferon-inducible protein 16 OS=Homo sapiens GN=IFI16 PE=1 SV=3 - [IF16_HUMAN]	1.26E-05	1.36E-03	up	1.8990318
P55265	Double-stranded RNA-specific adenosine deaminase OS=Homo sapiens GN=ADAR PE=1 SV=4 - [DSRAD_HUMAN]	1.00E-04	2.01E-03	up	1.4333954
P61421	V-type proton ATPase subunit d 1 OS=Homo sapiens GN=ATP6V0D1 PE=1 SV=1 - [VA0D1_HUMAN]	4.58E-04	4.06E-03	up	1.5366842

Q6UXH1	Cysteine-rich with EGF-like domain protein 2 OS=Homo sapiens GN=CRELD2 PE=1 SV=1 - [CREL2_HUMAN]	6.74E-04	5.11E-03	up	2.1996548
Q15021	Condensin complex subunit 1 OS=Homo sapiens GN=NCAPD2 PE=1 SV=3 - [CND1_HUMAN]	7.50E-04	5.41E-03	down	-1.3730582
Q9BWS9	Chitinase domain-containing protein 1 OS=Homo sapiens GN=CHID1 PE=1 SV=1 - [CHID1_HUMAN]	5.47E-04	4.58E-03	up	1.3908793
Q9NZT2	Opioid growth factor receptor OS=Homo sapiens GN=OGFR PE=1 SV=3 - [OGFR_HUMAN]	9.10E-03	2.65E-02	up	1.1740304
O60610	Protein diaphanous homolog 1 OS=Homo sapiens GN=DIAPH1 PE=1 SV=2 - [DIAP1_HUMAN]	1.96E-02	4.56E-02	down	-1.1406935
O14684	Prostaglandin E synthase OS=Homo sapiens GN=PTGES PE=1 SV=2 - [PTGES_HUMAN]	3.80E-03	1.48E-02	up	3.5154588
P27701	CD82 antigen OS=Homo sapiens GN=CD82 PE=1 SV=1 - [CD82_HUMAN]	1.61E-03	8.31E-03	up	1.598229
O76027	Annexin A9 OS=Homo sapiens GN=ANXA9 PE=1 SV=3 - [ANXA9_HUMAN]	7.57E-05	1.91E-03	up	2.784713
Q92879	CUGBP Elav-like family member 1 OS=Homo sapiens GN=CELF1 PE=1 SV=2 - [CELF1_HUMAN]	9.71E-04	6.16E-03	down	-1.2149919
P06865	Beta-hexosaminidase subunit alpha OS=Homo sapiens GN=HEXA PE=1 SV=2 - [HEXA_HUMAN]	1.99E-05	1.36E-03	up	2.9524713
Q14134	Tripartite motif-containing protein 29 OS=Homo sapiens GN=TRIM29 PE=1 SV=2 - [TRI29_HUMAN]	1.13E-02	3.10E-02	down	-1.1202435
Q9UBB4	Ataxin-10 OS=Homo sapiens GN=ATXN10 PE=1 SV=1 - [ATX10_HUMAN]	1.86E-03	9.03E-03	up	1.5241088
Q9BQ39	ATP-dependent RNA helicase DDX50 OS=Homo sapiens GN=DDX50 PE=1 SV=1 - [DDX50_HUMAN]	6.10E-03	2.03E-02	down	-1.8190496
O60749	Sorting nexin-2 OS=Homo sapiens GN=SNX2 PE=1 SV=2 - [SNX2_HUMAN]	3.62E-04	3.61E-03	up	1.3565215
Q9BW04	Specifically androgen-regulated gene protein OS=Homo sapiens GN=SARG PE=1 SV=2 - [SARG_HUMAN]	2.92E-03	1.27E-02	down	-1.5747366
P00390	Glutathione reductase, mitochondrial OS=Homo sapiens GN=GSR PE=1 SV=2 - [GSHR_HUMAN]	6.31E-05	1.78E-03	up	2.4924774
P45983	Mitogen-activated protein kinase 8 OS=Homo sapiens GN=MAPK8 PE=1 SV=2 - [MK08_HUMAN]	2.55E-04	3.08E-03	down	-2.2800934
Q99848	Probable rRNA-processing protein EBP2 OS=Homo sapiens GN=EBNA1BP2 PE=1 SV=2 - [EBP2_HUMAN]	7.70E-04	5.51E-03	down	-1.6622479
P51659	Peroxisomal multifunctional enzyme type 2 OS=Homo sapiens GN=HSD17B4 PE=1 SV=3 - [DHB4_HUMAN]	5.55E-05	1.78E-03	up	1.5258179
Q14978	Nucleolar and coiled-body phosphoprotein 1 OS=Homo sapiens GN=NOLC1 PE=1 SV=2 - [NOLC1_HUMAN]	8.47E-05	1.93E-03	down	-1.6888846
Q9UHY1	Nuclear receptor-binding protein OS=Homo sapiens GN=NRBP1 PE=1 SV=1 - [NRBP_HUMAN]	9.43E-03	2.71E-02	up	1.3206558
P15104	Glutamine synthetase OS=Homo sapiens GN=GLUL PE=1 SV=4 - [GLNA_HUMAN]	4.48E-03	1.65E-02	up	1.3073772
Q8IUR0	Trafficking protein particle complex subunit 5 OS=Homo sapiens GN=TRAPPC5 PE=1 SV=1 - [TPPC5_HUMAN]	6.42E-03	2.09E-02	up	1.3734064
P13798	Acylamino-acid-releasing enzyme OS=Homo sapiens GN=APEH PE=1 SV=4 - [ACPH_HUMAN]	1.64E-02	4.02E-02	down	-1.1407412
Q9UI12	V-type proton ATPase subunit H OS=Homo sapiens GN=ATP6V1H PE=1 SV=1 - [VATH_HUMAN]	1.57E-03	8.21E-03	up	1.4507337
P51648	Fatty aldehyde dehydrogenase OS=Homo sapiens GN=ALDH3A2 PE=1 SV=1 - [AL3A2_HUMAN]	1.94E-04	2.70E-03	down	-1.3439752
P50402	Emerin OS=Homo sapiens GN=EMD PE=1 SV=1 - [EMD_HUMAN]	7.09E-03	2.22E-02	down	-1.5698254
P49189	4-trimethylaminobutyraldehyde dehydrogenase OS=Homo sapiens GN=ALDH9A1 PE=1 SV=3 - [AL9A1_HUMAN]	1.59E-05	1.36E-03	up	1.4587952
Q9Y3A4	Ribosomal RNA-processing protein 7 homolog A OS=Homo sapiens GN=RRP7A PE=1 SV=2 - [RRP7A_HUMAN]	9.10E-03	2.65E-02	down	-1.2266407
P52306	Rap1 GTPase-GDP dissociation stimulator 1 OS=Homo sapiens GN=RAP1GDS1 PE=1 SV=3 - [GDS1_HUMAN]	1.56E-02	3.85E-02	down	-1.121545
Q08AF3	Schlafen family member 5 OS=Homo sapiens GN=SLFN5 PE=1 SV=1 - [SLFN5_HUMAN]	7.92E-04	5.60E-03	up	1.9826893
O75643	U5 small nuclear ribonucleoprotein 200 kDa helicase OS=Homo sapiens GN=SNRNP200 PE=1 SV=2 - [U520_HUMAN]	9.52E-03	2.73E-02	down	-1.1944007
Q9H4A5	Golgi phosphoprotein 3-like OS=Homo sapiens GN=GOLPH3L PE=1 SV=1 - [GLP3L_HUMAN]	7.45E-03	2.27E-02	up	1.9586179
Q9BPX3	Condensin complex subunit 3 OS=Homo sapiens GN=NCAPG PE=1 SV=1 - [CND3_HUMAN]	9.49E-04	6.11E-03	down	-1.3318311

Q7L1Q6	Basic leucine zipper and W2 domain-containing protein 1 OS=Homo sapiens GN=BZW1 PE=1 SV=1 - [BZW1_HUMAN]	1.14E-03	6.82E-03	down	-1.6815206
Q9Y276	Mitochondrial chaperone BCS1 OS=Homo sapiens GN=BCS1L PE=1 SV=1 - [BCS1_HUMAN]	1.65E-03	8.39E-03	down	-1.7372831
P51159	Ras-related protein Rab-27A OS=Homo sapiens GN=RAB27A PE=1 SV=3 - [RB27A_HUMAN]	5.87E-03	1.98E-02	up	1.4642688
P49642	DNA primase small subunit OS=Homo sapiens GN=PRIM1 PE=1 SV=1 - [PRI1_HUMAN]	8.14E-04	5.71E-03	down	-1.7366599
P48449	Lanosterol synthase OS=Homo sapiens GN=LSS PE=1 SV=1 - [ERG7_HUMAN]	1.62E-03	8.34E-03	up	1.4240606
Q8NFH3	Nucleoporin Nup43 OS=Homo sapiens GN=NUP43 PE=1 SV=1 - [NUP43_HUMAN]	8.42E-03	2.51E-02	down	-1.2197393
O15533	Tapasin OS=Homo sapiens GN=TAPBP PE=1 SV=1 - [TPSN_HUMAN]	8.24E-04	5.71E-03	up	2.064553
Q6NUK1	Calcium-binding mitochondrial carrier protein SCaMC-1 OS=Homo sapiens GN=SLC25A24 PE=1 SV=2 - [SCMC1_HUMAN]	9.74E-05	2.01E-03	down	-1.4782157
Q86U38	Nucleolar protein 9 OS=Homo sapiens GN=NOP9 PE=1 SV=1 - [NOP9_HUMAN]	4.46E-03	1.65E-02	up	1.517769
P49959	Double-strand break repair protein MRE11 OS=Homo sapiens GN=MRE11 PE=1 SV=3 - [MRE11_HUMAN]	5.43E-03	1.88E-02	up	1.2570578
Q9UDY2	Tight junction protein ZO-2 OS=Homo sapiens GN=TJP2 PE=1 SV=2 - [ZO2_HUMAN]	2.01E-04	2.73E-03	up	1.7104182
P11182	Lipoamide acyltransferase component of branched-chain alpha-keto acid dehydrogenase complex, mitochondrial OS=Homo sapiens GN=DBT PE=1 SV=3 - [ODB2_HUMAN]	1.92E-02	4.50E-02	down	-1.4291689
O95747	Serine/threonine-protein kinase OSR1 OS=Homo sapiens GN=OXSR1 PE=1 SV=1 - [OXSR1_HUMAN]	1.02E-03	6.38E-03	up	1.2839477
P57768	Sorting nexin-16 OS=Homo sapiens GN=SNX16 PE=1 SV=2 - [SNX16_HUMAN]	1.95E-02	4.56E-02	up	1.4950898
P46459	Vesicle-fusing ATPase OS=Homo sapiens GN=NSF PE=1 SV=3 - [NSF_HUMAN]	7.70E-06	1.35E-03	up	1.8119555
O14744	Protein arginine N-methyltransferase 5 OS=Homo sapiens GN=PRMT5 PE=1 SV=4 - [ANM5_HUMAN]	1.65E-02	4.03E-02	down	-1.2156925
O60762	Dolichol-phosphate mannosyltransferase subunit 1 OS=Homo sapiens GN=DPM1 PE=1 SV=1 - [DPM1_HUMAN]	7.37E-03	2.27E-02	up	1.2151911
O95478	Ribosome biogenesis protein NSA2 homolog OS=Homo sapiens GN=NSA2 PE=1 SV=1 - [NSA2_HUMAN]	8.41E-03	2.51E-02	down	-1.2447759
Q13308	Inactive tyrosine-protein kinase 7 OS=Homo sapiens GN=PTK7 PE=1 SV=2 - [PTK7_HUMAN]	1.23E-03	7.10E-03	down	-1.2785714
P35580	Myosin-10 OS=Homo sapiens GN=MYH10 PE=1 SV=3 - [MYH10_HUMAN]	2.49E-04	3.05E-03	down	-1.4624026
Q99747	Gamma-soluble NSF attachment protein OS=Homo sapiens GN=NAPG PE=1 SV=1 - [SNAG_HUMAN]	1.99E-06	8.34E-04	up	1.301351
P31937	3-hydroxyisobutyrate dehydrogenase, mitochondrial OS=Homo sapiens GN=HIBADH PE=1 SV=2 - [3HIDH_HUMAN]	1.25E-03	7.12E-03	up	1.3404347
Q9Y285	Phenylalanine--tRNA ligase alpha subunit OS=Homo sapiens GN=FARSA PE=1 SV=3 - [SYFA_HUMAN]	2.13E-02	4.85E-02	down	-1.2721947
P25685	DnaJ homolog subfamily B member 1 OS=Homo sapiens GN=DNAJB1 PE=1 SV=4 - [DNJB1_HUMAN]	2.55E-05	1.44E-03	up	1.2354383
Q9NRJ5	Poly(A) polymerase beta OS=Homo sapiens GN=PAPOLB PE=2 SV=1 - [PAPOB_HUMAN]	4.30E-05	1.78E-03	down	-1.7611123
Q9HCC0	Methylcrotonoyl-CoA carboxylase beta chain, mitochondrial OS=Homo sapiens GN=MCCC2 PE=1 SV=1 - [MCCB_HUMAN]	1.55E-02	3.84E-02	up	1.4034
O60906	Sphingomyelin phosphodiesterase 2 OS=Homo sapiens GN=SMPD2 PE=1 SV=2 - [NSMA_HUMAN]	2.00E-03	9.60E-03	up	1.2967778
O95834	Echinoderm microtubule-associated protein-like 2 OS=Homo sapiens GN=EML2 PE=1 SV=1 - [EMAL2_HUMAN]	1.24E-03	7.10E-03	up	1.4021201
O14787	Transportin-2 OS=Homo sapiens GN=TNPO2 PE=1 SV=3 - [TNPO2_HUMAN]	1.04E-03	6.44E-03	up	1.4744554
P48735	Isocitrate dehydrogenase [NADP], mitochondrial OS=Homo sapiens GN=IDH2 PE=1 SV=2 - [IDHP_HUMAN]	2.26E-04	2.86E-03	up	1.4327364
Q9UIA9	Exportin-7 OS=Homo sapiens GN=XPO7 PE=1 SV=3 - [XPO7_HUMAN]	1.15E-03	6.83E-03	up	1.2675384
Q9NSE4	Isoleucine--tRNA ligase, mitochondrial OS=Homo sapiens GN=IARS2 PE=1 SV=2 - [SYIM_HUMAN]	4.46E-03	1.65E-02	up	1.7426889
Q16186	Proteasomal ubiquitin receptor ADRM1 OS=Homo sapiens GN=ADRM1 PE=1 SV=2 - [ADRM1_HUMAN]	2.97E-03	1.28E-02	down	-1.2287655
Q2TAY7	WD40 repeat-containing protein SMU1 OS=Homo sapiens GN=SMU1 PE=1 SV=2 - [SMU1_HUMAN]	1.40E-02	3.58E-02	down	-1.2146875

Q16831	Uridine phosphorylase 1 OS=Homo sapiens GN=UPP1 PE=1 SV=1 - [UPP1_HUMAN]	1.17E-03	6.92E-03	up	1.583172
Q9Y6K5	2'-5'-oligoadenylate synthase 3 OS=Homo sapiens GN=OAS3 PE=1 SV=3 - [OAS3_HUMAN]	9.42E-05	1.99E-03	up	1.3342782
Q9Y6M1	Insulin-like growth factor 2 mRNA-binding protein 2 OS=Homo sapiens GN=IGF2BP2 PE=1 SV=2 - [IF2B2_HUMAN]	9.42E-03	2.71E-02	down	-3.6895058
Q5C9Z4	Nucleolar MIF4G domain-containing protein 1 OS=Homo sapiens GN=NOM1 PE=1 SV=1 - [NOM1_HUMAN]	1.08E-03	6.59E-03	up	1.6000432
Q9H9A6	Leucine-rich repeat-containing protein 40 OS=Homo sapiens GN=LRRC40 PE=1 SV=1 - [LRC40_HUMAN]	0.00E+00	0.00E+00	down	-16
Q9UJ70	N-acetyl-D-glucosamine kinase OS=Homo sapiens GN=NAGK PE=1 SV=4 - [NAGK_HUMAN]	3.27E-03	1.36E-02	up	1.7269212
Q9H6W3	Ribosomal oxygenase 1 OS=Homo sapiens GN=RIOX1 PE=1 SV=2 - [RIOX1_HUMAN]	6.41E-03	2.09E-02	down	-1.1208888
Q86V21	Acetoacetyl-CoA synthetase OS=Homo sapiens GN=AACS PE=1 SV=1 - [AACS_HUMAN]	1.64E-05	1.36E-03	down	-2.0512118
Q6PI48	Aspartate--tRNA ligase, mitochondrial OS=Homo sapiens GN=DARS2 PE=1 SV=1 - [SYDM_HUMAN]	9.37E-04	6.11E-03	up	1.5726361
Q9Y3Z3	Deoxynucleoside triphosphate triphosphohydrolase SAMHD1 OS=Homo sapiens GN=SAMHD1 PE=1 SV=2 - [SAMH1_HUMAN]	3.71E-04	3.61E-03	up	1.4277416
O75400	Pre-mRNA-processing factor 40 homolog A OS=Homo sapiens GN=PRPF40A PE=1 SV=2 - [PR40A_HUMAN]	2.01E-02	4.65E-02	down	-1.1814973
O15127	Secretory carrier-associated membrane protein 2 OS=Homo sapiens GN=SCAMP2 PE=1 SV=2 - [SCAM2_HUMAN]	3.00E-03	1.28E-02	up	1.3049076
Q9ULX3	RNA-binding protein NOB1 OS=Homo sapiens GN=NOB1 PE=1 SV=1 - [NOB1_HUMAN]	2.22E-04	2.84E-03	down	-1.5035427
O14896	Interferon regulatory factor 6 OS=Homo sapiens GN=IRF6 PE=1 SV=1 - [IRF6_HUMAN]	2.95E-03	1.28E-02	up	1.4686584
O60264	SWI/SNF-related matrix-associated actin-dependent regulator of chromatin subfamily A member 5 OS=Homo sapiens GN=SMARCA5 PE=1 SV=1 - [SMCA5_HUMAN]	1.43E-02	3.65E-02	down	-1.2105724
O94925	Glutaminase kidney isoform, mitochondrial OS=Homo sapiens GN=GLS PE=1 SV=1 - [GLSK_HUMAN]	4.17E-04	3.84E-03	down	-1.2727522
Q96S44	TP53-regulating kinase OS=Homo sapiens GN=TP53RK PE=1 SV=2 - [PRPK_HUMAN]	3.50E-03	1.41E-02	up	1.2900254
P52566	Rho GDP-dissociation inhibitor 2 OS=Homo sapiens GN=ARHGDIB PE=1 SV=3 - [GDIR2_HUMAN]	1.86E-05	1.36E-03	up	2.3920696
P22307	Non-specific lipid-transfer protein OS=Homo sapiens GN=SCP2 PE=1 SV=2 - [NLTP_HUMAN]	9.14E-03	2.65E-02	down	-1.2692349
Q9P2R7	Succinate--CoA ligase [ADP-forming] subunit beta, mitochondrial OS=Homo sapiens GN=SUCLA2 PE=1 SV=3 - [SUCB1_HUMAN]	9.00E-05	1.93E-03	up	1.4459355
Q93050	V-type proton ATPase 116 kDa subunit a isoform 1 OS=Homo sapiens GN=ATP6V0A1 PE=1 SV=3 - [VPP1_HUMAN]	1.23E-04	2.21E-03	up	1.9227544
Q08722	Leukocyte surface antigen CD47 OS=Homo sapiens GN=CD47 PE=1 SV=1 - [CD47_HUMAN]	2.18E-04	2.81E-03	up	1.6584599
Q9Y5A7	NEDD8 ultimate buster 1 OS=Homo sapiens GN=NUB1 PE=1 SV=2 - [NUB1_HUMAN]	1.24E-02	3.29E-02	up	1.7533697
O43663	Protein regulator of cytokinesis 1 OS=Homo sapiens GN=PRC1 PE=1 SV=2 - [PRC1_HUMAN]	6.62E-04	5.11E-03	down	-1.7139791
Q13045	Protein flightless-1 homolog OS=Homo sapiens GN=FLII PE=1 SV=2 - [FLII_HUMAN]	4.69E-03	1.68E-02	down	-1.2113197
Q96N66	Lysophospholipid acyltransferase 7 OS=Homo sapiens GN=MBOAT7 PE=1 SV=2 - [MBOA7_HUMAN]	1.21E-02	3.25E-02	down	-1.274538
Q15003	Condensin complex subunit 2 OS=Homo sapiens GN=NCAPH PE=1 SV=3 - [CND2_HUMAN]	1.01E-02	2.85E-02	down	-1.472106
P46087	Probable 28S rRNA (cytosine(4447)-C(5))-methyltransferase OS=Homo sapiens GN=NOP2 PE=1 SV=2 - [NOP2_HUMAN]	8.93E-03	2.62E-02	down	-1.1640453
Q9Y6M7	Sodium bicarbonate cotransporter 3 OS=Homo sapiens GN=SLC4A7 PE=1 SV=2 - [S4A7_HUMAN]	2.01E-02	4.65E-02	down	-1.1880937
Q9GZR7	ATP-dependent RNA helicase DDX24 OS=Homo sapiens GN=DDX24 PE=1 SV=1 - [DDX24_HUMAN]	2.95E-03	1.28E-02	down	-1.3916662
Q16775	Hydroxyacylglutathione hydrolase, mitochondrial OS=Homo sapiens GN=HAGH PE=1 SV=2 - [GLO2_HUMAN]	1.33E-03	7.37E-03	up	1.845707
P27694	Replication protein A 70 kDa DNA-binding subunit OS=Homo sapiens GN=RPA1 PE=1 SV=2 - [RFA1_HUMAN]	3.38E-03	1.39E-02	down	-1.3268305
Q15020	Squamous cell carcinoma antigen recognized by T-cells 3 OS=Homo sapiens GN=SART3 PE=1 SV=1 - [SART3_HUMAN]	1.51E-03	8.02E-03	up	1.1961639
Q9UNQ2	Probable dimethyladenosine transferase OS=Homo sapiens GN=DIMT1 PE=1 SV=1 - [DIM1_HUMAN]	1.78E-02	4.25E-02	down	-1.1958653

Q9BSJ8	Extended synaptotagmin-1 OS=Homo sapiens GN=ESYT1 PE=1 SV=1 - [ESYT1_HUMAN]	9.08E-03	2.65E-02	down	-1.1641192
Q96SQ9	Cytochrome P450 2S1 OS=Homo sapiens GN=CYP2S1 PE=1 SV=2 - [CP2S1_HUMAN]	4.65E-03	1.68E-02	down	-1.2561288
Q14956	Transmembrane glycoprotein NMB OS=Homo sapiens GN=GPNMB PE=1 SV=2 - [GPNMB_HUMAN]	3.20E-03	1.34E-02	up	3.8122861
Q13416	Origin recognition complex subunit 2 OS=Homo sapiens GN=ORC2 PE=1 SV=2 - [ORC2_HUMAN]	1.15E-02	3.14E-02	down	-1.5569828
O95347	Structural maintenance of chromosomes protein 2 OS=Homo sapiens GN=SMC2 PE=1 SV=2 - [SMC2_HUMAN]	9.79E-03	2.77E-02	down	-1.2051346
Q9BRR6	ADP-dependent glucokinase OS=Homo sapiens GN=ADPGK PE=1 SV=1 - [ADPGK_HUMAN]	7.05E-03	2.22E-02	up	1.2796909
P16070	CD44 antigen OS=Homo sapiens GN=CD44 PE=1 SV=3 - [CD44_HUMAN]	7.18E-03	2.24E-02	down	-1.4455993
P54687	Branched-chain-amino-acid aminotransferase, cytosolic OS=Homo sapiens GN=BCAT1 PE=1 SV=3 - [BCAT1_HUMAN]	1.80E-03	8.88E-03	up	1.7024701
P16278	Beta-galactosidase OS=Homo sapiens GN=GLB1 PE=1 SV=2 - [BGAL_HUMAN]	1.68E-05	1.36E-03	up	2.674876
Q9UJU6	Drebrin-like protein OS=Homo sapiens GN=DBNL PE=1 SV=1 - [DBNL_HUMAN]	6.18E-03	2.05E-02	down	-1.4999853
P27708	CAD protein OS=Homo sapiens GN=CAD PE=1 SV=3 - [PYR1_HUMAN]	5.77E-03	1.97E-02	down	-1.4113257
Q9H4I3	TraB domain-containing protein OS=Homo sapiens GN=TRABD PE=1 SV=1 - [TRABD_HUMAN]	1.04E-02	2.90E-02	up	1.2781523
O43865	S-adenosylhomocysteine hydrolase-like protein 1 OS=Homo sapiens GN=AHCYL1 PE=1 SV=2 - [SAHH2_HUMAN]	6.29E-04	4.96E-03	down	-1.2098899
Q02880	DNA topoisomerase 2-beta OS=Homo sapiens GN=TOP2B PE=1 SV=3 - [TOP2B_HUMAN]	1.22E-02	3.26E-02	up	1.2403648
Q13033	Striatin-3 OS=Homo sapiens GN=STRN3 PE=1 SV=3 - [STRN3_HUMAN]	9.75E-03	2.77E-02	down	-1.2795827
Q8NBM4	Ubiquitin-associated domain-containing protein 2 OS=Homo sapiens GN=UBAC2 PE=1 SV=1 - [UBAC2_HUMAN]	1.22E-02	3.26E-02	up	1.1888158
P0DMP2	SLIT-ROBO Rho GTPase-activating protein 2B OS=Homo sapiens GN=SRGAP2B PE=3 SV=1 - [SRG2B_HUMAN]	9.83E-03	2.78E-02	up	1.8545426
Q14534	Squalene monooxygenase OS=Homo sapiens GN=SQLE PE=1 SV=3 - [ERG1_HUMAN]	5.62E-04	4.64E-03	up	1.6891527
Q5H9R7	Serine/threonine-protein phosphatase 6 regulatory subunit 3 OS=Homo sapiens GN=PPP6R3 PE=1 SV=2 - [PP6R3_HUMAN]	3.03E-05	1.63E-03	down	-1.119556
Q8NI36	WD repeat-containing protein 36 OS=Homo sapiens GN=WDR36 PE=1 SV=1 - [WDR36_HUMAN]	8.51E-03	2.53E-02	down	-1.5635501
Q5K651	Sterile alpha motif domain-containing protein 9 OS=Homo sapiens GN=SAMD9 PE=1 SV=1 - [SAMD9_HUMAN]	1.84E-02	4.35E-02	down	-1.5779779
P78347	General transcription factor II-I OS=Homo sapiens GN=GTF2I PE=1 SV=2 - [GTF2I_HUMAN]	1.34E-03	7.37E-03	down	-1.1755334
Q13823	Nucleolar GTP-binding protein 2 OS=Homo sapiens GN=GNL2 PE=1 SV=1 - [NOG2_HUMAN]	6.56E-04	5.10E-03	down	-1.3253435
Q9UHL4	Dipeptidyl peptidase 2 OS=Homo sapiens GN=DPP7 PE=1 SV=3 - [DPP2_HUMAN]	5.16E-04	4.41E-03	up	1.5832589
Q13188	Serine/threonine-protein kinase 3 OS=Homo sapiens GN=STK3 PE=1 SV=2 - [STK3_HUMAN]	4.66E-03	1.68E-02	up	1.9148835
Q9Y2T3	Guanine deaminase OS=Homo sapiens GN=GDA PE=1 SV=1 - [GUAD_HUMAN]	2.31E-03	1.07E-02	up	2.248735
P53985	Monocarboxylate transporter 1 OS=Homo sapiens GN=SLC16A1 PE=1 SV=3 - [MOT1_HUMAN]	1.49E-02	3.76E-02	down	-1.4588302
O95400	CD2 antigen cytoplasmic tail-binding protein 2 OS=Homo sapiens GN=CD2BP2 PE=1 SV=1 - [CD2B2_HUMAN]	4.61E-03	1.67E-02	down	-1.4955467
Q02040	A-kinase anchor protein 17A OS=Homo sapiens GN=AKAP17A PE=1 SV=2 - [AK17A_HUMAN]	5.98E-04	4.82E-03	up	1.7645462
Q96JB2	Conserved oligomeric Golgi complex subunit 3 OS=Homo sapiens GN=COG3 PE=1 SV=3 - [COG3_HUMAN]	1.46E-03	7.84E-03	up	1.3927218
O94832	Unconventional myosin-Id OS=Homo sapiens GN=MYO1D PE=1 SV=2 - [MYO1D_HUMAN]	8.98E-03	2.63E-02	up	1.6863748
Q15643	Thyroid receptor-interacting protein 11 OS=Homo sapiens GN=TRIP11 PE=1 SV=3 - [TRIPB_HUMAN]	6.09E-03	2.03E-02	down	-1.2959384
Q92609	TBC1 domain family member 5 OS=Homo sapiens GN=TBC1D5 PE=1 SV=1 - [TBCD5_HUMAN]	1.02E-04	2.02E-03	up	1.5077848
Q8N442	Translation factor GUF1, mitochondrial OS=Homo sapiens GN=GUF1 PE=1 SV=1 - [GUF1_HUMAN]	1.34E-03	7.37E-03	up	1.5227385

P12955	Xaa-Pro dipeptidase OS=Homo sapiens GN=PEPD PE=1 SV=3 - [PEPD_HUMAN]	1.19E-02	3.21E-02	up	1.461973
P80303	Nucleobindin-2 OS=Homo sapiens GN=NUCB2 PE=1 SV=2 - [NUCB2_HUMAN]	1.87E-03	9.03E-03	up	1.867417
Q9NTJ3	Structural maintenance of chromosomes protein 4 OS=Homo sapiens GN=SMC4 PE=1 SV=2 - [SMC4_HUMAN]	9.91E-04	6.22E-03	down	-1.351252
Q92538	Golgi-specific brefeldin A-resistance guanine nucleotide exchange factor 1 OS=Homo sapiens GN=GBF1 PE=1 SV=2 - [GBF1_HUMAN]	1.72E-03	8.57E-03	down	-1.4695753
Q5T160	Probable arginine--tRNA ligase, mitochondrial OS=Homo sapiens GN=RARS2 PE=1 SV=1 - [SYRM_HUMAN]	6.44E-03	2.09E-02	up	1.3845218
O75815	Breast cancer anti-estrogen resistance protein 3 OS=Homo sapiens GN=BCAR3 PE=1 SV=1 - [BCAR3_HUMAN]	2.90E-03	1.27E-02	down	-1.8781439
Q96AY3	Peptidyl-prolyl cis-trans isomerase FKBP10 OS=Homo sapiens GN=FKBP10 PE=1 SV=1 - [FKB10_HUMAN]	4.07E-04	3.77E-03	down	-1.9798253
O15131	Importin subunit alpha-6 OS=Homo sapiens GN=KPNA5 PE=1 SV=2 - [IMA6_HUMAN]	1.24E-03	7.10E-03	down	-1.4166344
Q7Z406	Myosin-14 OS=Homo sapiens GN=MYH14 PE=1 SV=2 - [MYH14_HUMAN]	8.23E-04	5.71E-03	up	1.4848014
Q01650	Large neutral amino acids transporter small subunit 1 OS=Homo sapiens GN=SLC7A5 PE=1 SV=2 - [LAT1_HUMAN]	2.12E-02	4.84E-02	down	-1.2028178
P11388	DNA topoisomerase 2-alpha OS=Homo sapiens GN=TOP2A PE=1 SV=3 - [TOP2A_HUMAN]	3.62E-03	1.44E-02	down	-1.4372797
P18583	Protein SON OS=Homo sapiens GN=SON PE=1 SV=4 - [SON_HUMAN]	3.83E-03	1.49E-02	down	-1.358802
Q02241	Kinesin-like protein KIF23 OS=Homo sapiens GN=KIF23 PE=1 SV=3 - [KIF23_HUMAN]	1.83E-02	4.34E-02	down	-1.435417
P51649	Succinate-semialdehyde dehydrogenase, mitochondrial OS=Homo sapiens GN=ALDH5A1 PE=1 SV=2 - [SSDH_HUMAN]	6.95E-04	5.21E-03	up	2.3108363
O75153	Clustered mitochondria protein homolog OS=Homo sapiens GN=CLUH PE=1 SV=2 - [CLU_HUMAN]	2.65E-03	1.19E-02	down	-1.6971428
Q63HN8	E3 ubiquitin-protein ligase RNF213 OS=Homo sapiens GN=RNF213 PE=1 SV=3 - [RN213_HUMAN]	3.18E-04	3.42E-03	up	2.2583714
Q12768	WASH complex subunit 5 OS=Homo sapiens GN=WASHC5 PE=1 SV=1 - [WASC5_HUMAN]	4.64E-03	1.68E-02	up	1.2072117
Q9H6T3	RNA polymerase II-associated protein 3 OS=Homo sapiens GN=RPAP3 PE=1 SV=2 - [RPAP3_HUMAN]	1.73E-02	4.14E-02	down	-1.3079689
Q9H9E3	Conserved oligomeric Golgi complex subunit 4 OS=Homo sapiens GN=COG4 PE=1 SV=3 - [COG4_HUMAN]	2.23E-03	1.05E-02	up	1.1368095
O60341	Lysine-specific histone demethylase 1A OS=Homo sapiens GN=KDM1A PE=1 SV=2 - [KDM1A_HUMAN]	5.99E-04	4.82E-03	up	1.5121465
A0MZ66	Shootin-1 OS=Homo sapiens GN=SHTN1 PE=1 SV=4 - [SHOT1_HUMAN]	2.05E-03	9.81E-03	down	-1.4179077
O14617	AP-3 complex subunit delta-1 OS=Homo sapiens GN=AP3D1 PE=1 SV=1 - [AP3D1_HUMAN]	1.82E-02	4.32E-02	up	1.3282548
Q6IA69	Glutamine-dependent NAD(+) synthetase OS=Homo sapiens GN=NADSYN1 PE=1 SV=3 - [NADE_HUMAN]	8.40E-03	2.51E-02	up	1.7894685
Q15436	Protein transport protein Sec23A OS=Homo sapiens GN=SEC23A PE=1 SV=2 - [SC23A_HUMAN]	3.21E-03	1.34E-02	down	-1.1793545
O14964	Hepatocyte growth factor-regulated tyrosine kinase substrate OS=Homo sapiens GN=HGS PE=1 SV=1 - [HGS_HUMAN]	2.16E-02	4.92E-02	up	1.2667841
P26006	Integrin alpha-3 OS=Homo sapiens GN=ITGA3 PE=1 SV=5 - [ITA3_HUMAN]	7.39E-03	2.27E-02	down	-1.2150097
Q8IZL8	Proline-, glutamic acid- and leucine-rich protein 1 OS=Homo sapiens GN=PELP1 PE=1 SV=2 - [PELP1_HUMAN]	2.70E-03	1.20E-02	up	1.2021981
Q13409	Cytoplasmic dynein 1 intermediate chain 2 OS=Homo sapiens GN=DYNC1I2 PE=1 SV=3 - [DC1I2_HUMAN]	2.48E-03	1.13E-02	up	1.6033486
Q9Y5K6	CD2-associated protein OS=Homo sapiens GN=CD2AP PE=1 SV=1 - [CD2AP_HUMAN]	1.33E-03	7.37E-03	up	1.1512592
Q92922	SWI/SNF complex subunit SMARCC1 OS=Homo sapiens GN=SMARCC1 PE=1 SV=3 - [SMRC1_HUMAN]	4.36E-03	1.63E-02	down	-1.512285
P26358	DNA (cytosine-5)-methyltransferase 1 OS=Homo sapiens GN=DNMT1 PE=1 SV=2 - [DNMT1_HUMAN]	2.08E-04	2.79E-03	down	-1.7789246
P06396	Gelsolin OS=Homo sapiens GN=GSN PE=1 SV=1 - [GELS_HUMAN]	1.79E-02	4.27E-02	up	1.2887648
Q32P28	Prolyl 3-hydroxylase 1 OS=Homo sapiens GN=P3H1 PE=1 SV=2 - [P3H1_HUMAN]	2.98E-03	1.28E-02	down	-2.4484682
Q5UIP0	Telomere-associated protein RIF1 OS=Homo sapiens GN=RIF1 PE=1 SV=2 - [RIF1_HUMAN]	8.06E-05	1.93E-03	down	-2.498625

Q9NW13	RNA-binding protein 28 OS=Homo sapiens GN=RBM28 PE=1 SV=3 - [RBM28_HUMAN]	2.03E-04	2.73E-03	down	-1.2218133
Q03519	Antigen peptide transporter 2 OS=Homo sapiens GN=TAP2 PE=1 SV=1 - [TAP2_HUMAN]	5.73E-04	4.65E-03	up	2.892575
Q93034	Cullin-5 OS=Homo sapiens GN=CUL5 PE=1 SV=4 - [CUL5_HUMAN]	1.02E-02	2.87E-02	up	1.3159928
Q5T0N5	Formin-binding protein 1-like OS=Homo sapiens GN=FNBP1L PE=1 SV=3 - [FBP1L_HUMAN]	2.36E-04	2.93E-03	down	-1.4343518
Q9NVI1	Fanconi anemia group I protein OS=Homo sapiens GN=FANCI PE=1 SV=4 - [FANCI_HUMAN]	1.67E-02	4.04E-02	down	-1.2644907
Q8IYB3	Serine/arginine repetitive matrix protein 1 OS=Homo sapiens GN=SRRM1 PE=1 SV=2 - [SRRM1_HUMAN]	3.91E-03	1.51E-02	down	-1.440327
O95202	LETM1 and EF-hand domain-containing protein 1, mitochondrial OS=Homo sapiens GN=LETM1 PE=1 SV=1 - [LETM1_HUMAN]	1.46E-02	3.68E-02	up	1.1514128
O95104	Splicing factor, arginine/serine-rich 15 OS=Homo sapiens GN=SCAF4 PE=1 SV=3 - [SFR15_HUMAN]	1.23E-02	3.27E-02	up	1.2844826
Q9Y4W6	AFG3-like protein 2 OS=Homo sapiens GN=AFG3L2 PE=1 SV=2 - [AFG32_HUMAN]	6.36E-03	2.08E-02	up	1.4096694
P78344	Eukaryotic translation initiation factor 4 gamma 2 OS=Homo sapiens GN=EIF4G2 PE=1 SV=1 - [IF4G2_HUMAN]	1.41E-03	7.65E-03	down	-1.3584572
Q14690	Protein RRP5 homolog OS=Homo sapiens GN=PDCD11 PE=1 SV=3 - [RRP5_HUMAN]	1.16E-03	6.86E-03	down	-1.2546092
Q08378	Golgin subfamily A member 3 OS=Homo sapiens GN=GOLGA3 PE=1 SV=2 - [GOGA3_HUMAN]	1.18E-03	6.92E-03	down	-1.2846271
Q9Y6D6	Brefeldin A-inhibited guanine nucleotide-exchange protein 1 OS=Homo sapiens GN=ARFGEF1 PE=1 SV=2 - [BIG1_HUMAN]	1.22E-02	3.26E-02	up	1.5689297
Q9Y3R5	Protein dopey-2 OS=Homo sapiens GN=DOPEY2 PE=1 SV=5 - [DOP2_HUMAN]	7.37E-03	2.27E-02	up	1.7305596
Q9UDT6	CAP-Gly domain-containing linker protein 2 OS=Homo sapiens GN=CLIP2 PE=1 SV=1 - [CLIP2_HUMAN]	1.03E-02	2.88E-02	down	-1.2741673
P09884	DNA polymerase alpha catalytic subunit OS=Homo sapiens GN=POLA1 PE=1 SV=2 - [DPOLA_HUMAN]	7.88E-03	2.39E-02	down	-2.207869
Q9Y4B6	DDB1- and CUL4-associated factor 1 OS=Homo sapiens GN=DCAF1 PE=1 SV=3 - [DCAF1_HUMAN]	6.24E-03	2.06E-02	up	1.6140684
Q14689	Disco-interacting protein 2 homolog A OS=Homo sapiens GN=DIP2A PE=1 SV=2 - [DIP2A_HUMAN]	1.79E-03	8.88E-03	down	-1.2471621
Q9Y5S2	Serine/threonine-protein kinase MRCK beta OS=Homo sapiens GN=CDC42BPB PE=1 SV=2 - [MRCKB_HUMAN]	3.70E-03	1.46E-02	up	1.5261877
Q9ULL4	Plexin-B3 OS=Homo sapiens GN=PLXNB3 PE=1 SV=2 - [PLXB3_HUMAN]	1.60E-02	3.92E-02	up	1.1816063
